# TRIM29 hypermethylation drives esophageal cancer progression via suppression of ZNF750

**DOI:** 10.1038/s41420-023-01491-1

**Published:** 2023-06-26

**Authors:** Qiyi Yi, Yujia Zhao, Ran Xia, Qinqin Wei, Fengmei Chao, Rui Zhang, Po Bian, Lei Lv

**Affiliations:** 1 School of Basic Medical Sciences, Anhui Medical University, 230032 Hefei, Anhui China; 2 Department of education training, The First People’s Hospital of Changzhou, The Third Affiliated Hospital of Soochow University, Changzhou, Jiangsu China; 3 Department of Cancer Epigenetics Program, Anhui Cancer Hospital, The First Affiliated Hospital of USTC, Division of Life Sciences and Medicine, University of Science and Technology of China, 230031 Hefei, Anhui China; 4 Department of Oncology, The First Affiliated Hospital of Anhui University of Chinese Medicine, 230031 Hefei, Anhui China

**Keywords:** Oesophageal cancer, Oncogenesis, Prognostic markers, Metastasis

## Abstract

Esophageal cancer (ESCA) is the seventh most frequent and deadly neoplasm. Due to the lack of early diagnosis and high invasion/metastasis, the prognosis of ESCA remains very poor. Herein, we identify skin-related signatures as the most deficient signatures in invasive ESCA, which are regulated by the transcription factor ZNF750. Of note, we find that TRIM29 level strongly correlated with the expression of many genes in the skin-related signatures, including ZNF750. TRIM29 is significantly down-regulated due to hypermethylation of its promoter in both ESCA and precancerous lesions compared to normal tissues. Low TRIM29 expression and high methylation levels of its promoter are associated with malignant progression and poor clinical outcomes in ESCA patients. Functionally, TRIM29 overexpression markedly hinders proliferation, migration, invasion, and epithelial–mesenchymal transition of esophageal cancer cells, whereas opposing results are observed when TRIM29 is silenced in vitro. In addition, TRIM29 inhibits metastasis in vivo. Mechanistically, TRIM29 downregulation suppresses the expression of the tumor suppressor ZNF750 by activating the STAT3 signaling pathway. Overall, our study demonstrates that TRIM29 expression and its promoter methylation status could be potential early diagnostic and prognostic markers. It highlights the role of the TRIM29-ZNF750 signaling axis in modulating tumorigenesis and metastasis of esophageal cancer.

## Introduction

ESCA is a frequently diagnosed malignancy of gastrointestinal cancer, with an estimated more than 600,000 new cases and 540,000 deaths worldwide in 2020 [1]. In China, about 246,000 new cases were diagnosed as ESCA, and about 188,000 people died of the disease in 2015 [2]. ESCA is a type of epithelial cancer and originates from lesions of esophageal mucosa [3], which is composed of squamous cells similar to those of the skin. It has two main subtypes: ~10% are esophageal adenocarcinoma (EAC) and ~90% are esophageal squamous cell carcinoma (ESCC) [4]. In most cases, ESCC patients are diagnosed in advanced stages due to the absence of early clinical symptoms. Moreover, advanced ESCC often accompanies with invasion and distant metastasis, leading to a high mortality rate of ESCC patients, with a 5-year survival rate of 12% [5–7]. Through large-scale sequencing of ESCC, many genomic and epigenomic abnormalities have been identified, such as abnormal expression and mutations of oncogenes and tumor suppressors, and changes in DNA methylation [4, 8–10]. However, further research is still required to pinpoint the mechanisms behind ESCC tumorigenesis and progression, especially for metastasis. It would then be possible to identify novel biomarkers for diagnostic/prognostic purposes, as well as effective therapeutic targets, which could be beneficial for treating esophageal cancer.

ZNF750 (Zinc Finger Protein 750) is a novel zinc-finger transcription factor and regulates skin homeostasis by finely driving epidermal differentiation [11–13]. *ZNF750* was often mutated and underexpressed in ESCA [9, 14, 15], which might give selective advantages to ESCA cells and promote the progress of ESCA [16]. Moreover, it was also reported as a potential predictor of survival of patients with ESCA [17, 18]. ZNF750 deficiency significantly correlated with lymph node metastasis in ESCC [17], and ZNF750 could hinder invasion through binding and inhibiting SNAI1, a factor crucial for epithelial-mesenchymal transition, in ESCA patients [19]. However, the underlying mechanism of ZNF750 deficiency in ESCA remains unclear.

TRIM29 mediates diverse physiological and pathological processes, including cell differentiation, immunity, and cancer [20–24]. However, its role in different cancers is elusive. TRIM29 has been shown to have oncogenic effects in pancreatic, lung, endometrial, bladder, gastric, and colorectal cancers [25–32]. For example, it promoted the proliferation of lung cancer cells [26] and the invasion of pancreatic ductal adenocarcinoma cells, colorectal cancer, and bladder cancer [27, 30–32]. In contrast, TRIM29 could suppress tumorigenesis in other cancer types. For example, its expression was lower in prostate cancers than in normal tissues [33], and it was often silenced and inhibited the invasive behavior in breast cancers and squamous cell carcinoma [34, 35]. Thus, TRIM29 has a complex role depending on the type of cancer and the microenvironment, and its role in ESCA remains unclear.

This study examined the expression, clinical significance, and biological functions of TRIM29 in esophageal cancer. We found that TRIM29 expression was frequently down-regulated, induced by promoter hypermethylation in esophageal cancer and precancerous lesions. In addition, TRIM29 deficiency correlated with aggressive phenotype and poor prognosis in esophageal cancer. TRIM29 hindered the proliferation, migration, invasion, metastasis, and EMT of esophageal cancer. Furthermore, we elaborated on the possible mechanism that TRIM29 induces the expression of ZNF750 via modulating the activation of STAT3. These findings uncover a novel mechanism of progression and metastasis of ESCC mediated by the TRIM29-STAT3-ZNF750-Snail axis, and also suggest that TRIM29 expression and the methylation status of its promoter may serve as early diagnostic and prognostic biomarkers for ESCC.

## Results

### TRIM29 expression highly correlates with ZNF750 expression and the invasive phenotype of esophageal cancer

**Fig. 1:**
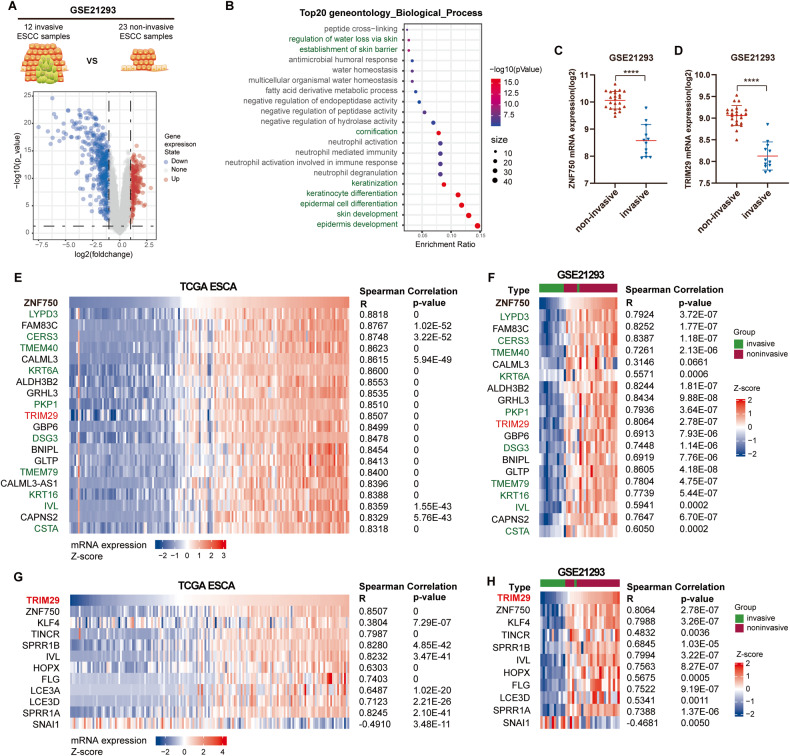
TRIM29 expression highly correlated with ZNF750 expression and invasive phenotype of esophageal cancer. **A** Volcano plot for DEGs (differentially expressed genes) identified in invasive ESCC samples from GSE21293 dataset (red plots: upregulated DEGs; blue plots: downregulated DEGs). **B** Gene ontology analysis of the downregulated DEGs in invasive samples (green characters: skin-related signatures). **C**, **D** Comparison of ZNF750 and TRIM29 levels between noninvasive and invasive ESCC samples in GSE21293, respectively. **E** The correlation heatmap represented the top 20 genes whose expression most correlated with ZNF750 expression in TCGA ESCA. **F** The correlation between ZNF750 and analyzed genes of **E** in GSE21293 was represented by a correlation heatmap. **G**, **H** The heatmaps show the expression correlation between TRIM29 and ZNF750-related genes in TCGA ESCA (**G**) and GSE21293 (**H**) datasets. The samples were ordered left-to-right by increasing ZNF750 level in **E**, **F** and TRIM29 level in (G&H), respectively. The relative ratios of expression level were indicated by color.

In order to explore the critical molecular changes associated with the invasive transition of esophageal cancer, GSE21293, an ESCC dataset containing 23 noninvasive and 12 invasive ESCC samples, was used for comprehensive analysis. Through the gene differential expression analysis of invasive vs. noninvasive samples, 286 significantly upregulated genes and 417 significantly down-regulated genes were identified in invasive tumors (Fig. 1A). Gene Ontology enrichment analysis revealed that the down-regulated genes were mostly enriched in skin-related signatures, including epidermis development, skin development, epidermal cell differentiation, keratinocyte differentiation, and keratinization (Fig. 1B and Table S1). Deficiency in these genes often leads to invasion and metastasis [36]. Among these genes, ZNF750 deficiency in invasive samples was particularly noticeable (Fig. 1C). ZNF750 is a zinc-finger transcription factor essential for epidermal differentiation and could regulate the expression of a variety of keratin proteins [11–13]. Moreover, ZNF750 functions as a tumor suppressor through inhibiting the proliferation, invasion, and metastasis of ESCC. Its mutation and downregulation are one of the most frequent molecular abnormalities in esophageal cancer [9, 14, 19, 37]. These results suggested that ZNF750 plays key roles in inhibiting invasion and metastasis of esophageal cancer.

To identify genes that might regulate ZNF750 expression in esophageal cancer, Spearman’s correlation analysis between *ZNF750* expression and all other genes was conducted in TCGA ESCA (Table S2). Of the top 20 genes whose expression most correlated with *ZNF750* expression in esophageal cancer, 10 were epidermal differentiation and keratinization-related genes, such as *LYPD3*, *KRT6A*, *KRT16*, and *IVL* (Fig. 1E and Table S2). Of note, *TRIM29*, whose expression was also significantly reduced in invasive ESCC (Fig. 1D), also had a strong positive correlation with *ZNF750* expression (*R* = 0.8507, *p* = 0, Fig. 1E). Moreover, *TRIM29* expression positively correlated with expression of *KLF4*, *TINCR*, *SPRR1B*, *IVL*, *HOPX*, *FLG*, *LCE3A*, *LCE3D*, and *SPRR1A* (Fig. 1G), which have previously been reported to be positively regulated by ZNF750 [11, 37, 38]. And, *TRIM29* expression negatively correlated with *SNAI1* expression (Fig. 1G), which has previously been reported to be negatively regulated by ZNF750 [19]. These results were also validated in GSE21293 (Fig. 1F, H) (*CALML3-AS1* and *LCE3A* were unavailable in GSE21293). Therefore, we hypothesized that TRIM29 might positively regulate the expression of ZNF750 and mediate its inhibitory function in invasion and metastasis of esophageal cancer.

### TRIM29 expression is frequently downregulated in esophageal cancer

**Fig. 2:**
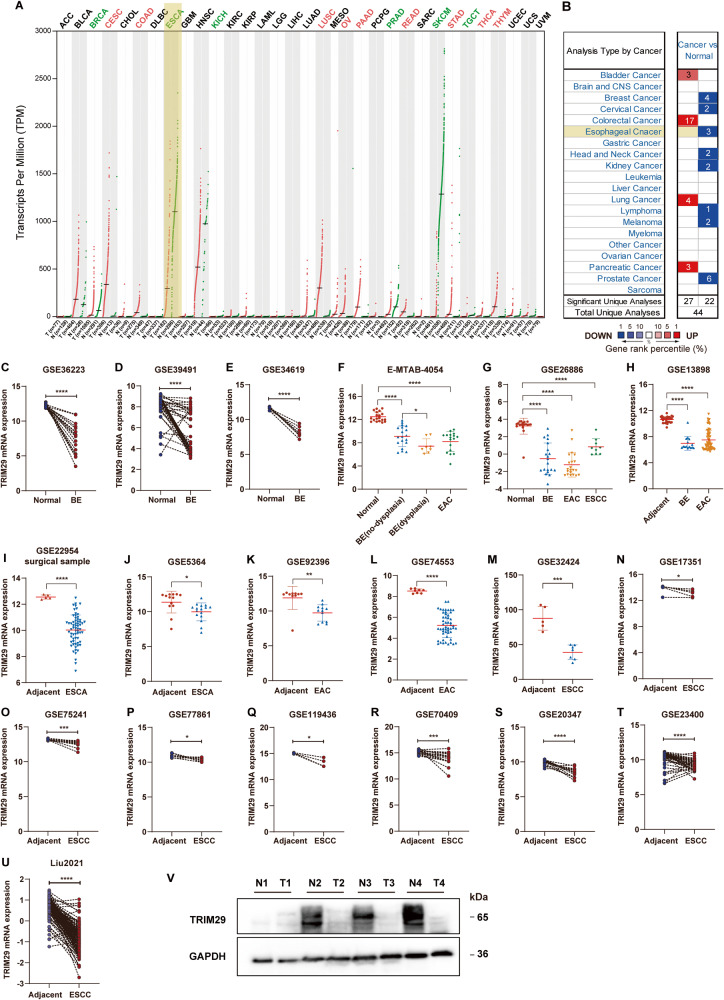
TRIM29 is lowly expressed in ESCA and its precursor lesions. **A** Expression profiles of TRIM29 across TCGA cancer types were analyzed using the GEPIA (red dots: tumor specimens; green dots: normal specimens; red characters: expression increased in tumors, green characters: expression decreased in tumors). **B** Expression profiles of TRIM29 across multiple cancer types were analyzed in Oncomine (number: the number of datasets; red: expression increased in tumors; blue: expression decreased in tumors). **C**–**T** Comparison of TRIM29 mRNA levels among adjacent non-cancerous tissues, BE, ESCA, ESCC, and EAC in 18 datasets from GEO and Arrayexpress. **U** Comparison of TRIM29 protein levels between adjacent non-cancerous tissues and ESCC samples in “Liu2021” datasets. **V** The protein levels of TRIM29 in paired paracancerous and ESCC tissues were detected by western blot. *p < 0.05, **p < 0.01, ***p < 0.001, ****p < 0.0001.

Analysis through the GTEx portal showed that both TRIM29 and ZNF750 were expressed at a much higher level in skin, esophagus mucosa, and vagina than in other human tissues (Fig. S1A, B). In addition, TRIM29 and ZNF750 expression positively correlated in normal skin and esophagus mucosa tissues (Fig. S1C–E). The results indicated that TRIM29 plays key roles in the development of esophagus mucosa. Therefore, abnormalities in TRIM29 are likely to contribute to ESCA progression. Comparisons of TRIM29 mRNA levels between tumor and normal tissues in TCGA showed that TRIM29 expression was significantly higher in multiple tumor types compared to the adjacent non-tumor tissues, including CESC, COAD, LUSC, OV, PAAD, READ, STAD, THCA, and THYM, while significantly lower in some other types of tumors, including ESCA, BRCA, KICH, PRAD, SKCM, and TGCT (Fig. 2A). Furthermore, the TRIM29 mRNA levels in multiple cancer types were also analyzed through the Oncomine platform. Among the 20 assigned cancer types compared with normal tissues, under the criteria of a fold‑change of 1.5 and *p*-value of 0.05, TRIM29 mRNA levels were downregulated in 22 datasets with eight different types of cancer, including three datasets of esophageal cancer (Fig. 2B).

We then examined the TRIM29 mRNA expression in 18 ESCA datasets from GEO and Arrayexpress. The results showed that TRIM29 expression in precancerous lesions of ESCA, including Barrett’s esophagus (BE) and dysplasia, was significantly lower than in matched adjacent/normal esophageal epithelial tissues (Fig. 2C–H). Concordant with the results of TCGA and Oncomine, TRIM29 mRNA expression was also much lower in the ESCA tissues, including EAC and ESCC, compared with adjacent/normal tissues in all these datasets (Fig. 2F–T). Consistently, TRIM29 protein levels in ESCC samples were significantly lower than in paired normal esophageal tissues, according to the analysis of a protein dataset “Liu2021” (Fig. 2U). Through western blotting analysis of 4 pairs of ESCC and matched adjacent tissues, we also confirmed that TRIM29 levels were downregulated in cancerous tissues (Fig. 2V). In addition, no TRIM29 mutation was identified in ESCA after analyzing whole-exome sequencing data of TCGA ESCA through the cBioPortal webtool (Fig. S2).

Taken together, these results demonstrate that TRIM29 is downregulated in ESCA and its precursor lesions, highlighting that TRIM29 deficiency is a marker of early-stage esophageal cancer and may contribute to the tumorigenesis of ESCA.

### TRIM29 deficiency is associated with worse clinicopathological features and prognosis in ESCA

**Fig. 3:**
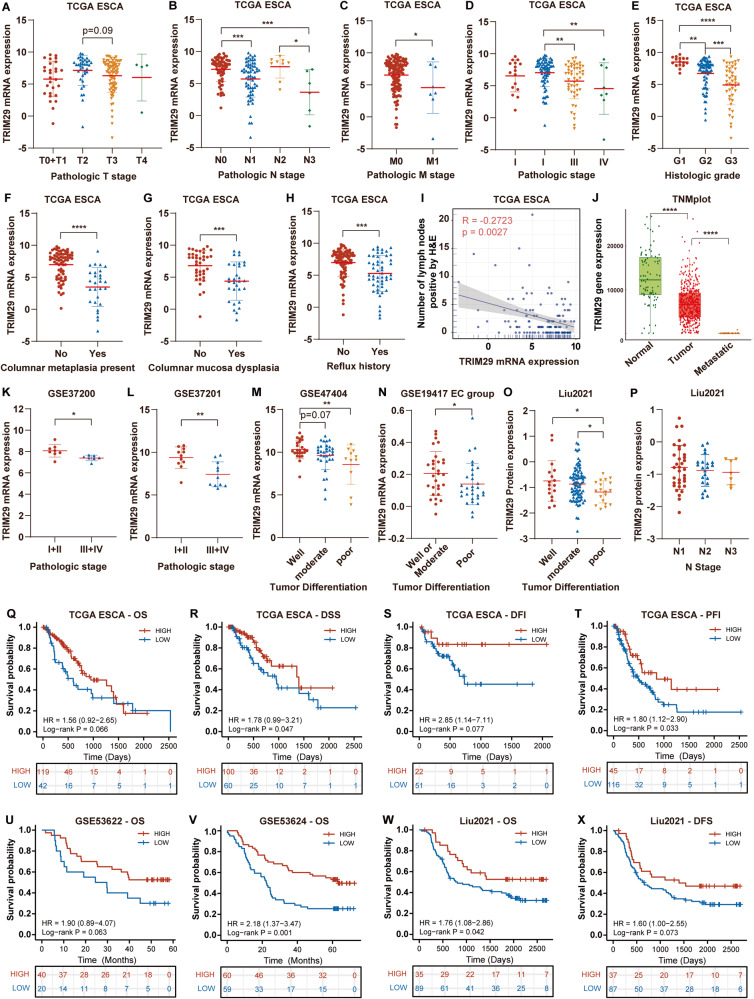
TRIM29 expression decreases with ESCA progression. **A**–**H** TRIM29 mRNA levels in different T stages (**A**), N stages (**B**), M stages (**C**), pathologic stages (**D**), histologic grade (**E**), columnar metaplasia present status (**F**), columnar mucosa dysplasia status (**G**), and reflux history (**H**) of ESCA samples in TCGA ESCA. **I** The correlation between TRIM29 mRNA level and the number of lymph nodes in TCGA ESCA. **J** TRIM29 mRNA level in different esophageal tissues was analyzed using TNMplot webtool. **K**, **L** TRIM29 mRNA levels in different pathologic stages of ESCA samples in GSE3700 and GSE37201 datasets. **M**, **N** TRIM29 mRNA levels in different tumor differentiation statuses of ESCA samples in GSE47404 and GSE19417 datasets. **O**, **P** TRIM29 protein levels in different tumor differentiation status and N stage of ESCA samples in Liu2021 dataset. **Q**–**T** Kaplan–Meier analysis of OS (Overall survival), PFS (Progression-free survival), RFS (Relapse-free survival), and DSS (Disease-specific survival) according to TRIM29 mRNA levels in TCGA ESCA. **U**, **V** Kaplan–Meier analysis of OS according to TRIM29 mRNA levels in GSE53622 and GSE53624 datasets. **W**, **X** Kaplan–Meier analysis of OS and DFS (disease-free survival) according to TRIM29 protein levels in Liu2021 dataset.

We then analyzed the relationship between TRIM29 expression levels and clinicopathological characteristics in multiple ESCA datasets, including five mRNA datasets (TCGA ESCA, GSE37200, GSE37201, GSE47404, and GSE19417) and a protein dataset (Liu2021). Low TRIM29 mRNA levels were associated with advanced T stage, N stage, M stage, pathologic stage, and histologic grade in TCGA ESCA (Fig. 3A-E), which was confirmed in GSE37200 and GSE37201 datasets (Fig. 3K, L). Analysis through TNMplot (https://tnmplot.com/analysis/) showed that TRIM29 mRNA level was significantly lower in metastatic ESCA samples than in primary tumors (Fig. 3J). And TRIM29 protein levels decreased in advanced N stage ESCA in Liu2021 dataset (Fig. 3P). TRIM29 mRNA level was significantly lower in ESCA with columnar metaplasia, columnar mucosa, and reflux history compared to their respective controls (Fig. 3F–H). Furthermore, it negatively correlated with the number of lymph nodes in TCGA ESCA (Fig. 3I). In addition, we found that both mRNA and protein levels of TRIM29 were impaired in poorly differentiated ESCA samples through analysis from GSE19417, GSE47404, and Liu2021 datasets (Fig. 3M–O).

Then, the prognostic value of TRIM29 expression in ESCA was also evaluated. As shown in plots using the Kaplan–Meier Plotter analysis, TRIM29 deficiency was associated with reduced OS (overall survival) (*p* = 0.066), DSS (Disease-specific survival) (*p* = 0.047), DFI (Disease-free interval) (*p* = 0.077), and PFI (progression-free interval) (*p* = 0.033) in TCGA ESCA (Fig. 3Q–T). Consistently, analysis from GSE53622 and GSE53624 also indicated a close association of lower TRIM29 mRNA levels with reduced OS in ESCC patients (*p* = 0.063, *p* = 0.001, respectively; Fig. 3U, V). Moreover, a trend towards decreased OS and DFS (disease-free survival) in patients expressing low protein levels of TRIM29 was also observed in Liu2021 dataset (*p* = 0.042; *p* = 0.073, respectively; Fig. 3W, X).

These results suggest that TRIM29 expression may serve as a prognostic and predictive marker for patients with ESCA.

### TRIM29 deficiency is due to its promoter hypermethylation in ESCA

**Fig. 4:**
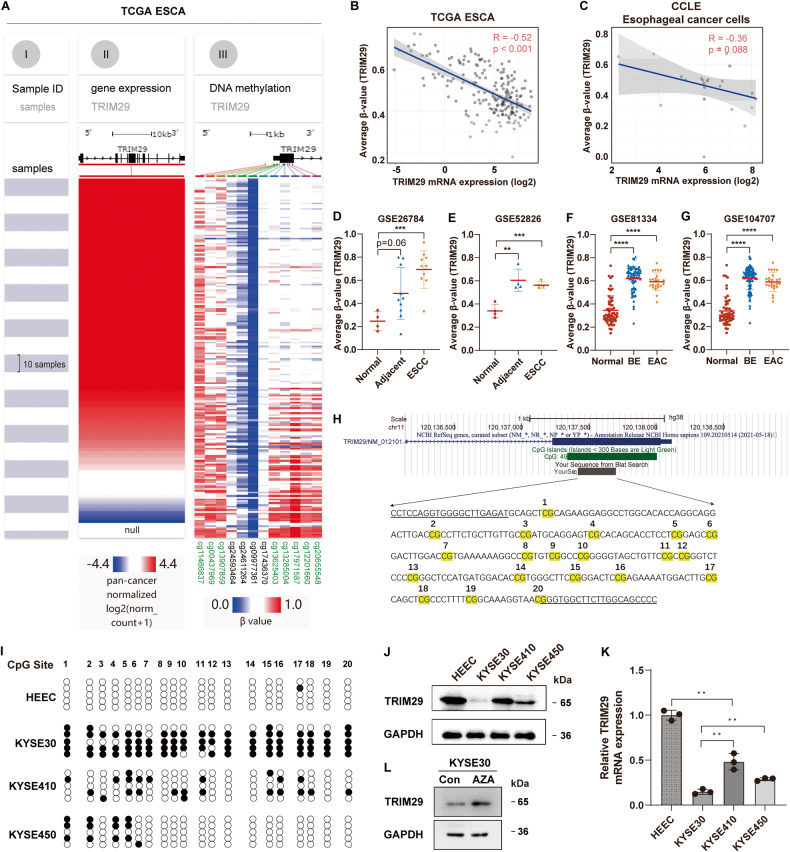
Hypermethylation of TRIM29 promoter leads to its deficiency in ESCA. **A** Heatmap of TRIM29 mRNA levels and β-value (methylation level) of 12 CpG sites in TRIM29 promoter. Samples were ordered from top to bottom by the TRIM29 mRNA levels. Each row indicates one sample. Blue: low level; Red: high level. **B**, **C** Scatterplot showing the positive correlation between TRIM29 mRNA expression and average β-value of CpG sites in TRIM29 promoter in TCGA ESCA and CCLE esophageal cancer cells. **D**–**G** Comparison of the average methylation level of TRIM29 promoter among different types of specimens in GSE26784, GSE52826, GSE81334, and GSE104707 datasets. **H** UCSC genome browser shows the location of TRIM29 gene and its upstream region. Twenty CpG sites within the CpG island of TRIM29 promotor region analyzed by BSP (bisulfite sequencing PCR) are indicated. **I** The methylation status of CpG sites in **H** were measured by BSP in HEEC, KYSE30, KYSE410, and KYSE450 cells. The methylated CpG sites were represented with filled circles, and the un-methylated CpG sites were represented with hollow circles. **J**, **K** TRIM29 protein and mRNA levels in HEEC, KYSE30, KYSE410, and KYSE450 were analyzed by WB and qRT–PCR. **L** Effect of 5-aza treatment on the TRIM29 levels in KYSE30.

Subsequently, we investigated the possible causes and mechanisms that lead to TRIM29 deficiency in ESCA. Gene expression is often negatively regulated by DNA methylation of its promoter, which is defined as −2 kb to +2 kb relative to TSS (transcription start site). Methylation alterations of 12 CpG loci in TRIM29 promoter, including cg11466837, cg00437969, cg13907859, cg24593464, cg24611264, cg09977361, cg17436370, cg13625403, cg13285004, cg17971587, cg12201660, and cg20655548, were analyzed in DNA methylation array of TCGA ESCA. Among these CpG loci, the methylation levels of 8 loci, including cg11466837, cg00437969, cg13907859, cg13625403, cg13285004, cg17971587, cg12201660, and cg20655548, negatively correlated with TRIM29 mRNA expression (Figs. 4A and S3), and the average methylation levels of all 12 CpG sites in TRIM29 promoter region were also negatively correlated with TRIM29 mRNA levels significantly in TCGA ESCA (*R* = −0.52, *p* < 0.001, Fig. 4B). In addition, the average methylation levels of CpG loci in TRIM29 promoter also negatively correlated with its mRNA expression in esophageal cancer cells of CCLE (Cancer Cell Line Encyclopedia; *R* = −0.36, *p* = 0.088, Fig. 4C).

The average methylation levels of all 12 CpG sites in TRIM29 promoter region were also analyzed in four DNA methylation datasets of ESCA, including GSE26784, GSE52826, GSE81334, and GSE104707. It showed that the methylation levels of TRIM29 promoter in esophageal cancer, including ESCC and EAC, were significantly higher than in normal tissues (Fig. 4D–G). In addition, its methylation levels in cancer-adjacent tissues and Barrett’s esophagus were also significantly higher than in normal tissues (Fig. 4D–G). These results indicated that TRIM29 promoter was hypermethylated in the early stage of ESCA, leading to TRIM29 deficiency.

To validate our findings in bioinformatic analysis, the expression and methylation levels of TRIM29 promoter in four cell lines (one normal esophageal cell line HEEC, and three ESCC cell lines KYSE30, KYSE410, and KYSE450) were analyzed. The genomic analysis of TRIM29 through the UCSC genome browser shows that there is a CpG island located in the first exon of TRIM29 (Fig. 4H). Bisulfite sequencing was applied to examine the methylation levels of selected CpG sites in this region. The results showed that hypermethylated KYSE30 cells had a low, while hypomethylated HEEC, KYSE410, and KYSE450 cells had a high endogenous protein and mRNA level of TRIM29 (Fig. 4I–K). It is noteworthy that the methylation level of TRIM29 promoter was negatively associated with TRIM29 expression levels (Fig. 4I–K). Treatment with 5-aza, a strong DNA demethylation inducer, upregulated the protein level of TRIM29 in KYSE30 cells significantly, confirming the negative regulation of TRIM29 expression by DNA methylation (Fig. 4L).

Furthermore, the high methylation level of several sites in TRIM29 promoter was associated with poor prognosis in TCGA ESCA (Fig. S4). Especially, high methylation levels of cg00437969 and cg20655548 were associated with shorter OS (Fig. S4A&B, *p* = 0.024 and *p* = 0.038, respectively) and DSS (Fig. S4C, D, *p* = 0.041 and *p* = 0.041, respectively) of patients with ESCA.

These results suggest hypermethylation of TRIM29 promoter contributes to TRIM29 deficiency in ESCA and precancerous lesions, suggesting it might be an early marker in the pathogenesis of ESCA.

### TRIM29 deficiency promotes ESCC cell proliferation

As more than 90% of ESCA cases are ESCC, we next explore the function of TRIM29 in ESCC. Firstly, HALLMARKs associated with TRIM29 expression were investigated by Gene Set Enrichment Analysis (GSEA) in two datasets, including GSE21293 and TCGA ESCC (ESCC samples of TCGA ESCA). The common HALLMARKs significantly associated with TRIM29 expression in both datasets were used for further analysis (FDR *q*-value < 0.25). Only one HALLMARK, “Peroxisome”, was positively associated with TRIM29 expression, while fifteen HALLMARKs, such as “Epithelial-mesenchymal transition”, “Angiogenesis”, “IL6-Jak-STAT3 signaling”, “G2-M checkpoint”, and “Mitotic spindle”, were negatively associated with TRIM29 expression (Table S3).

**Fig. 5:**
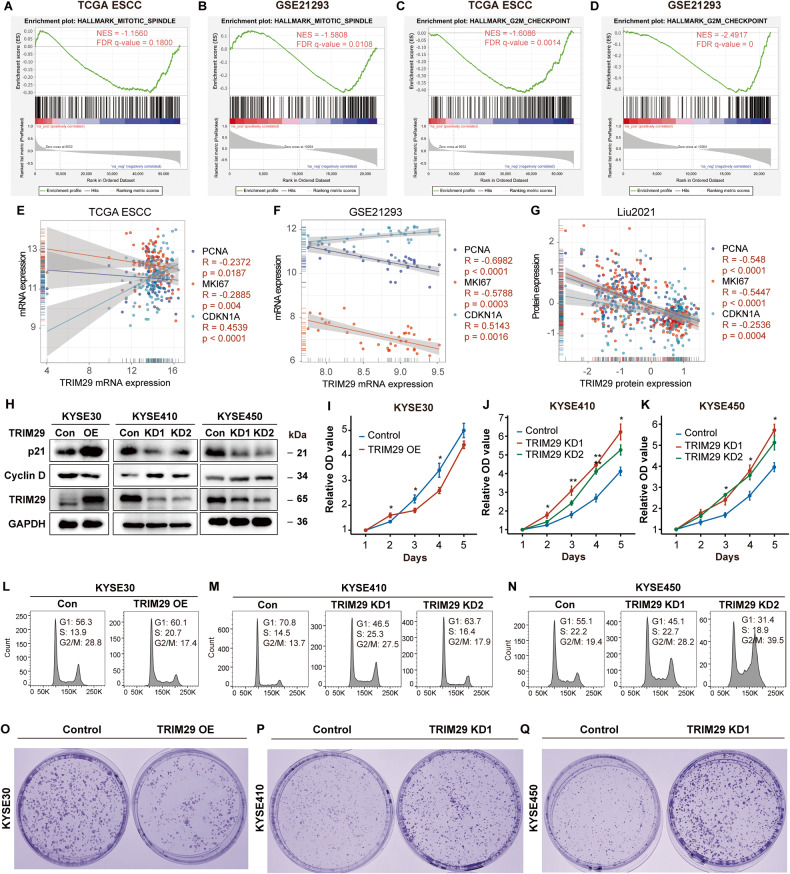
TRIM29 inhibits proliferation of ESCC cells. **A**, **B** GSEA analysis shows a negative correlation between TRIM29 mRNA levels and “HALLMARK MITOTIC SPINDLE” signature in TCGA ESCC and GSE21293. **C**, **D** GSEA analysis shows a negative correlation between TRIM29 mRNA levels and “HALLMARK G2M CHECKPOINT” signature in TCGA ESCC and GSE21293. **E**, **F** Scatterplot of mRNA expression correlation between TRIM29 and PCNA/MKI67/CDKN1A in TCGA ESCC and GSE21293. **G** Scatterplot of protein expression correlation between TRIM29 and PCNA/MKI67/CDKN1A in Liu2021 protein dataset. **H** Western blot analysis of p21 and Cyclin D protein levels after TRIM29 overexpression in KYSE30, and TRIM29 knockdown in KYSE410/KYSE450 cells. **I**–**K** The effect of TRIM29 expression on cell proliferation of KYSE30, KYSE410, and KYSE450 was evaluated by CCK8 assay. **L**–**N** Flow cytometric analysis of the effect of TRIM29 expression on cell cycle distribution in KYSE30, KYSE410, and KYSE450 cells. **O**–**Q** Representative images of colony formation assays in KYSE30 vs. TRIM29-OE KYSE30 cells (**O**), KYSE410 vs. TRIM29-KD KYSE410 cells (**P**), and KYSE450 vs. TRIM29-KD KYSE450 cells (**Q**), respectively. Con control, OE overexpression, KD knockdown.

Among the HALLMARKs negatively correlated with TRIM29 expression, two cellular signaling pathways were those implicated in cell cycle and proliferation, including “G2-M checkpoint” and “Mitotic spindle” (Fig. 5A–D). “G2-M checkpoint” and “Mitotic spindle” are both important events in mitosis, many genes included in these two HALLMARKs are highly expressed during mitosis. Through GSEA analysis, we found that in esophageal cancer tissues with low TRIM29 expression, many genes of these two HALLMARKs were highly expressed, suggesting that these tissues proliferated more vigorously. Furthermore, the expression of PCNA and MKI67 (Ki-67), two markers of mitosis, negatively correlated with TRIM29 at both mRNA and protein levels in TCGA ESCC, GSE21293, and Liu2021 datasets, while the mRNA level of *CDKN1A*, a cell cycle inhibitory gene, positively correlated with TRIM29 mRNA level in TCGA ESCC and GSE21293 (Fig. 5E–G). All these results indicated high proliferation rates in TRIM29-deficient ESCC.

To further validate these observations, KYSE30 was stably transfected with TRIM29-overexpression lentivirus, while KYSE410 and KYSE450 were transfected with TRIM29-shRNA lentivirus (Fig. 5H). TRIM29 overexpression increased the expression of p21 (*CDKN1A*), while decreased the level of cyclin D1, which drives cell-cycle progression, in KYSE30. Conversely, TRIM29 knockdown led to the opposite effect in KYSE410 and KYSE450 cells (Fig. 5H).

Functionally, the CCK-8 assays showed that TRIM29 overexpression impeded the proliferation of KYSE30 cells, while TRIM29 knockdown markedly promoted the proliferation of KYSE410 and KYSE450 cells (Fig. 5I–K). Furthermore, cell cycle analysis demonstrated that compared to wildtype KYSE410 and KYSE450, the TRIM29 knockdown cells displayed an accumulation in the G2/M phase, while TRIM29 overexpression in KYSE30 decreased the percentage of G2/M phase cells (Fig. 5L–N). In addition, soft agar colony formation efficiency was significantly inhibited by the overexpression of TRIM29 in KYSE30, while improved considerably by the knockdown of TRIM29 in KYSE410 and KYSE450 (Fig. 5O–Q).

Taken together, these data indicate that TRIM29 acts as a tumor suppressor to inhibit the proliferation of ESCC.

### TRIM29 deficiency promotes epithelial-mesenchymal transition, invasion, and metastasis of ESCC

**Fig. 6:**
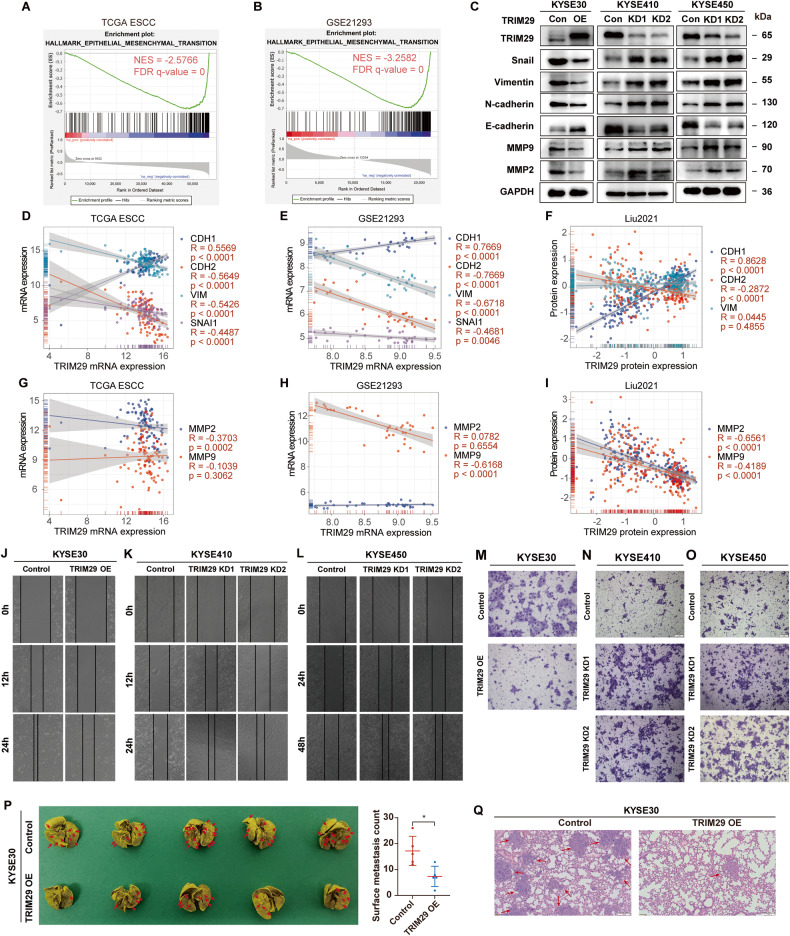
TRIM29 inhibits EMT, migration, invasion, and metastasis of ESCC cells. **A**, **B** GSEA analysis shows a negative correlation between TRIM29 mRNA levels and “HALLMARK EPITHELIAL MESENCHYMAL TRANSITION” signature in TCGA ESCC and GSE21293. **C** Western blot analysis of Snail, Vimentin, N-cadherin, E-cadherin, MMP9, and MMP2 protein levels after TRIM29 overexpression in KYSE30, and TRIM29 knockdown in KYSE410/KYSE450 cells. **D**, **E** Scatterplot of mRNA expression correlation between TRIM29 and CDH1/CDH2/VIM/SNAI1 in TCGA ESCC and GSE21293. **F** Scatterplot of protein expression correlation between TRIM29 and CDH1/CDH2/VIM in Liu2021 dataset. **G**, **H** Scatterplot of mRNA expression correlation between TRIM29 and MMP2/MMP9 in TCGA ESCC and GSE21293. **I** Scatterplot of protein expression correlation between TRIM29 and MMP2/MMP9 in Liu2021 dataset. **J**–**L** The effect of TRIM29 expression on cell migration of KYSE30, KYSE410, and KYSE450 was evaluated by wound-healing assay. **M**–**O** The effect of TRIM29 expression on cell invasion of KYSE30, KYSE410, and KYSE450 was evaluated by matrigel-transwell assay. **P** KYSE30 or TRIM29-overexpressing KYSE30 cells were injected into the tail vein of SCID/Beige mice (5 mice in each group). Left: the lungs were harvested 12 weeks post-injection, and the surface metastatic nodules were counted (red arrows: metastatic nodules on lung surface). Right: quantitative analysis of metastatic nodes on lung surface. *p < 0.05. **Q** Representative images of lung sections stained with H&E. OE overexpression, KD knockdown.

Low TRIM29 expression was associated with advanced N/M stages and lymph node metastasis (Fig. 3B, C, I, J, and P). Moreover, GSEA analysis showed that “Epithelial-mesenchymal transition” was the most negatively associated signature with TRIM29 levels in both TCGA ESCC and GSE21293 (Fig. 6A, B and Table S3). Thus, we hypothesized that TRIM29 loss could promote invasion and metastasis. To further assess the association of TRIM29 expression with ESCC metastasis, we first tested whether altered expression of TRIM29 was sufficient to change the EMT transcriptional program in ESCC cell lines. There was an increase in the level of E-cadherin, an epithelial marker, but a decrease in mesenchymal markers, specifically vimentin and N-cadherin, in TRIM29-overexpressing KYSE30 compared to control (Fig. 6C). Also, TRIM29 markedly suppressed Snail expression, which is an EMT-inducing transcription factor. Besides, TRIM29 inhibited the expression of MMP9 and MMP2, key proteins involved in stimulating invasion and metastasis. As expected, opposite protein expression profiles were observed in TRIM29-silencing KYSE410 and KYSE450 cells (Fig. 6C). In addition, correlation analysis in TCGA ESCC, GSE21293, and Liu2021 showed that both mRNA and protein levels of TRIM29 positively correlated with *CDH1* (E-cadherin), while negatively correlated with *CDH2* (N-cadherin), *VIM* (vimentin), *SNAI1* (Snail), MMP2 and MMP9 (Fig. 6D–I; *SNAI1* was not available in Liu2021 dataset). These results demonstrated that TRIM29 could inhibit EMT of ESCC cells in vitro, and that its expression negatively correlated with EMT phenotype of ESCC in vivo.

EMT often correlates with increased invasiveness and metastasis in tumors. Wound healing and transwell assays demonstrated that migration and invasion capacity were significantly inhibited after TRIM29 overexpression in KYSE30 cells (Fig. 6J, M), while enhanced after TRIM29 silencing in KYSE410 and KYSE450 (Fig. 6K, L, N, O). To assess the effect of TRIM29 on metastasis of ESCC cells in vivo, the number of metastatic nodules on lung surfaces was counted in mice injected with tumor cells through tail vein. TRIM29 overexpression dramatically impaired the potential of KYSE30 cells to metastasize to lungs (Fig. 6P). Moreover, the hematoxylin and eosin (H&E) staining examination confirmed the reduced lung metastases after TRIM29 overexpression (Fig. 6Q).

Collectively, these results indicate TRIM29 can inhibit EMT, invasion, and metastasis of ESCC both in vitro and in vivo.

### TRIM29 induces ZNF750 expression in ESCC

**Fig. 7:**
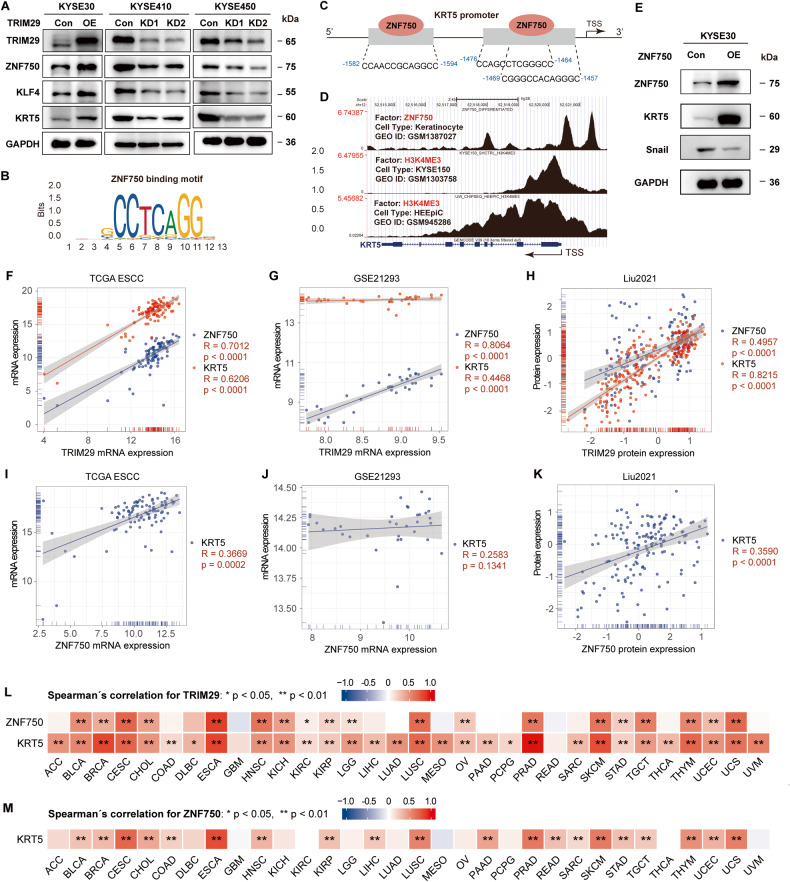
TRIM29 regulates the expression of ZNF750 and KRT5. **A** Western blot analysis of ZNF750, KLF4, and KRT5 protein levels after TRIM29 overexpression in KYSE30, and TRIM29 knockdown in KYSE410/KYSE450 cells. **B** Predictive motif of ZNF750 binding to gene promoter from JASPAR. **C** The schematic diagram of ZNF750 binding site in KRT5 promoter predicted by JASPAR. **D** Re-analysis of ZNF750 ChIP-seq data shows that ZNF750 binds to the promoter region of KRT5. H3K4me3 enrichment on KRT5 promoter of esophageal cells (KYSE150 and HEEpiC) was determined through analysis of two H3K4me3 ChIP-seq data. **E** Western blot analysis of ZNF750, KRT5, and Snail protein levels after ZNF750 overexpression in KYSE30. **F**, **G** Scatterplot of mRNA expression correlation between TRIM29 and ZNF750/KRT5 in TCGA ESCC and GSE21293. **H** Scatterplot of protein expression correlation between TRIM29 and ZNF750/KRT5 in Liu2021 dataset. **I**, **J** Scatterplot of mRNA expression correlation between ZNF750 and KRT5 in TCGA ESCC and GSE21293. **K** Scatterplot of protein expression correlation between ZNF750 and KRT5 in Liu2021 dataset. **L** The Spearman correlations of TRIM29 expression and ZNF750/KRT5 expression across 33 cancer types in TCGA. **M** The Spearman correlations of ZNF750 expression and KRT5 expression across 33 cancer types in TCGA.

Next, we aimed to identify the underlying molecular mechanism through which TRIM29 loss promotes ESCC progression. According to the analysis in Fig. 1, we hypothesized that TRIM29 might positively regulate the expression of tumor suppressor ZNF750, which inhibits cell proliferation, invasion, and metastasis of esophageal cancer [14, 16, 17, 19]. We then performed western blotting assays to detect changes in protein level of ZNF750 and its related genes following alterations in TRIM29 expression. It showed that TRIM29 exogenous overexpression stimulated protein expression of ZNF750 in KYSE30 cells, whereas TRIM29 silencing reduced ZNF750 expression in KYSE410 and KYSE450 cells (Fig. 7A). In addition, the expression of KLF4, a downstream protein of ZNF750 [11], showed very similar trends as that of ZNF750 (Fig. 7A). Moreover, we showed that both TRIM29 and ZNF750 inhibited the expression of Snail (Figs. 6C and 7E, respectively), which has been reported to be repressed by ZNF750 in a recent study [19].

ZNF750 is also a pivotal transcription factor for regulating the expression of multiple keratin proteins. Dysregulation of KRT5, a basal keratin marker, has been reported to promote cancer invasion and metastasis [35, 39, 40]. Analysis through JASPAR predicted three binding sites of ZNF750 on the KRT5 promoter (two sites have substantial overlap) (Fig. 7B, C). Analysis from two H2K4ME3 ChIP-sequencing datasets showed that the promoter of KRT5 in ESCC cell lines was active, and analysis from a ZNF750 ChIP-sequencing dataset indicated strong binding of ZNF750 in the promoter region of KRT5 (Fig. 7D). Besides, correlation analysis showed the positive relationship between TRIM29 and ZNF750, TRIM29 and KRT5, ZNF750 and KRT5 at both mRNA and protein levels in TCGA ESCC, GSE21293, and Liu2021 datasets (Fig. 7F–K). In addition, KRT5 expression could be remarkably induced by overexpression of TRIM29 or ZF750 in KYSE30 cells (Fig. 7A, E). Furthermore, pancancer analysis of 33 cancer types in TCGA revealed that TRIM29 expression positively correlated with ZNF750 and KRT5 levels in most cancer types (Fig. 7L). And, ZNF750 expression positively correlated with the KRT5 level in most cancer types (Fig. 7M).

Thus, these results suggest that TRIM29 deficiency leads to downregulation of ZNF750, which then modulates the expression of EMT-related proteins and epidermal differentiation/keratinization-related proteins (such as KRT5), thus promoting invasion and metastasis in ESCC.

### TRIM29 regulates tumor progression through inducing ZNF750 expression via inactivating STAT3 signaling

**Fig. 8:**
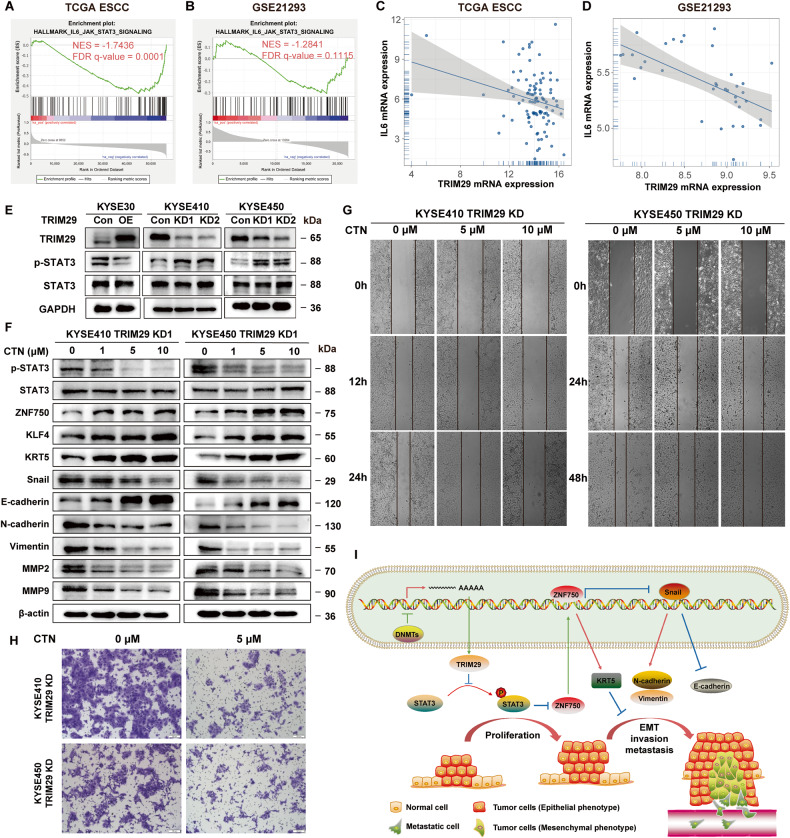
TRIM29 deficiency regulates ZNF750 expression and ESCC progression through activation of the IL6-STAT3 signaling pathway. **A**, **B** GSEA analysis shows a negative correlation between TRIM29 mRNA levels and “HALLMARK IL6 JAK STAT3 SIGNALING” signature in TCGA ESCC and GSE21293. **C**, **D** Scatterplot of mRNA expression correlation between TRIM29 and IL6 in TCGA ESCC and GSE21293. **E** Western blot analysis of STAT3 and p-STAT3(Tyr705) protein levels after TRIM29 overexpression in KYSE30, and TRIM29 knockdown in KYSE410/KYSE450 cells. **F** Western blot analysis of protein levels in STAT3, p-STAT3(Tyr705), ZNF750, KLF4, KRT5, Snail, E-cadherin, N-cadherin, Vimentin, MMP2, and MMP9 in TRIM29-knockdown KYSE410/KYSE450 cells treated with increasing concentrations of STAT3 inhibitor cryptotanshinone (CTN) for 24 h. **G**, **H** The effect of cryptotanshinone on cell migration and invasion of TRIM29-knockdown KYSE410/KYSE450 cells was evaluated by wound-healing assay and matrigel-transwell assay, respectively. **I** Schematic representation of the study.

We then sought to gain mechanistic insight into the regulation of ZNF750 by TRIM29. GSEA analysis in both TCGA ESCC and GSE21293 datasets showed that TRIM29 expression negatively correlated with the signature of “HALLMARK IL6-JAK-STAT3 SIGNALING” (Table S3 and Fig. 8A, B), which is often hyperactive in ESCC [41]. TRIM29 has been reported to inhibit cytokine production, such as IL6, and TRIM29 knockdown markedly enhanced the production of these cytokines [20, 21, 42, 43], which could activate STAT3 in multiple tumors, including esophageal cancer [44–47]. Consistent with this, correlation analysis in esophageal cancer samples showed that TRIM29 expression was negatively correlated with IL6 expression in TCGA ESCC and GSE21293 datasets (Fig. 8C, D). Indeed, STAT3 phosphorylation was dramatically inhibited by TRIM29 overexpression in KYSE30 cells, whereas it increased in TRIM29-silencing KYSE410 and KYSE450 cells (Fig. 8E).

To investigate whether the expression of ZNF750 is regulated by TRIM29 through IL6-STAT3 signaling pathway, cryptotanshinone (a specific p-STAT3 inhibitor) was applied to TRIM29-knockdown KYSE410 and KYSE450 cells. Western blot analysis showed that cryptotanshinone substantially suppressed phosphorylation level of STAT3, and enhanced the expression of ZNF750 in a dose-dependent manner (Fig. 8F). It also significantly upregulated the expression of KRT5 and KLF4, which are the downstream proteins of ZNF750. Furthermore, it inhibited the EMT and expression of MMP2/MMP9 in a dose-dependent manner (Fig. 8F). Functionally, cryptotanshinone reversed the pro-motile and pro-invasive effects of TRIM29-knockdown in KYSE410 and KYSE450 cells (Fig. 8G, H). These results indicated that the regulation of ZNF750 expression by TRIM29 in ESCC was mediated through IL6-STAT3 pathway.

Collectively, all these results suggest a model for how TRIM29 acts as a tumor suppressor via modulating ZNF750 expression in ESCC (Fig. 8I).

## Discussion

In this study, we assessed the transcriptional regulation of TRIM29 and its role in tumorigenesis and progression of ESCA through extensive bioinformatics analysis and in vitro/vivo experiments. TRIM29 was downregulated in ESCA relative to normal specimens, and the TRIM29 deficiency was associated with malignant clinicopathological characteristics in esophageal cancer, including advanced T/N/M stage, histologic grade, metastasis, and poor survival. Notably, this TRIM29 deficiency was caused by the hypermethylation of its promoter in esophageal cancer. Functionally, its deficiency promoted cell proliferation, EMT, migration, invasion of ESCC cell lines in vitro, and metastasis of esophageal cancer in vivo. Mechanistically, TRIM29 functioned as a positive regulator of tumor suppressor gene ZNF750 via modulating IL6/STAT3 signaling pathway. These results suggest that hypermethylation-induced TRIM29 deficiency is one of the critical factors regulating ESCC tumorigenesis and progression.

Metastasis is one of the top causes of cancer mortality. The 5-year survival rate ranges from 43.6% in ESCC patients without lymph node metastases to 29.3% in patients with it, and the survival rate declined further with larger extent, number, and size of metastatic lymph nodes in ESCC patients [48]. Targeting those cancer cells with high invasion/metastasis potential from the primary site may be a promising field in anti-metastasis treatment. Through an analysis of invasive vs. noninvasive ESCC samples, we explored a number of significantly differentially expressed genes, most of which were related to skin homeostasis. Similar to the skin, esophageal mucosa is composed of squamous cells that are the cellular origin of ESCC. Noteworthy, the expression of these genes is reported to be regulated by ZNF750. In addition, both TRIM29 and ZNF750 are lowly expressed in invasive samples.

ZNF750 could orchestrate the expression of numerous genes as a zinc-finger transcription factor. It plays an essential role in both normal cell biology and tumorigenesis. For example, ZNF750 is a key factor for regulating skin homeostasis by finely driving epidermal differentiation [11–13]. Esophageal mucosa is composed of squamous cells similar to those of the skin. We demonstrated that TRIM29 could regulate ZNF750 expression, and both ZNF750 and TRIM29 expression is significantly higher in esophageal mucosa and skin than in other tissues. In addition, we also showed that TRIM29 could regulate the expression of KLF4, which is also an important transcription factor required for normal function of the skin [49]. Moreover, we showed that TRIM29 could regulate the expression of KRT5, which is a keratin protein and plays an important role in protecting epithelial cells. Thus, our study indicated that TRIM29 might be an important regulator for skin development and homeostasis as an upstream regulator of ZNF750.

Many studies have revealed that the aberration of ZNF750 promoted the progression of multiple kinds of tumors, such as ESCC. Missense, truncating mutations, and genomic deletions of ZNF750 were observed in ESCC [9, 14, 16], and ZNF750 deficiency promoted cell proliferation and was associated with impaired differentiation in ESCC [14], which originates from the lesions of esophageal mucosa [3]. Low ZNF750 levels were associated with lymph node metastasis in ESCC [17], and downregulation of ZNF750 promotes angiogenesis, EMT, and invasion in ESCC [19, 50]. In addition, ZNF750 is associated with better survival and functions as a tumor suppressor in melanoma, nasopharyngeal carcinoma, oral squamous cell carcinoma, and squamous cell carcinoma [37, 51–55]. Mechanistically, ZNF750 has been reported to mediate tumor-suppressive roles by regulating the expression of various downstream genes, such as *TINCR*, *LAMC2*, *DANCR*, *FGF14*, and *SNAI1* [19, 37, 50, 51]. In this study, we found that TRIM29 could regulate the expression of snail, and EMT makers through promoting ZNF750 expression in ESCC. In addition, we also found that ZNF750 could directly bind the promoter and promote the expression of KRT5, dysregulation of which could promote cancer invasion and metastasis [35, 39, 40]. Furthermore, it is most likely that TRIM29 could regulate expression of ZNF750 and KRT5 in other tumors, as TRIM29 expression positively correlated with the expression of ZNF750 and KRT5 significantly in 18 tumor types, such as BLCA, CESC, HNSC, LUSC, PRAD, and SKCM. Thus, these results could explain why TRIM29 inhibits cell proliferation, invasion, and metastasis in ESCC and probably in other tumors.

Aberrant STAT3 signaling was observed in nearly 70% of cancers [56]. For example, one study reported detected nuclear phospho-STAT3 positivity in 71% of ESCC specimens [41]. STAT3 hyperactivation could promote tumor cell proliferation, survival, angiogenesis, invasiveness, and metastasis [56] and is often associated with poor patient outcomes [57, 58]. Inhibitors of IL-6/STAT3 signaling pathway have received FDA approval for various malignancies, or are currently in clinical and/or preclinical development [47]. Inhibition of STAT3 could effectively impede cell proliferation and migration of ESCA cells [41]. Similar findings were observed in our study, STAT3 inhibition could effectively reverse the cancer-promoting function of TRIM29 deficiency in ESCC. STAT3 is often activated by inflammatory factors, such as IL-6, IFN-β, and TNF-α. TRIM29 could target STING for ubiquitination and degradation as a ubiquitin E3 ligase, thus TRIM29 knockdown led to a remarkably elevated level of IL-6, IFN-β, and TNF-α [42]. Consistent with these results, we also found that TRIM29 expression negatively correlated with IL-6 expression in ESCC. Furthermore, IL6/STAT3 signaling pathway is involved in TRIM29 deficiency-mediated ZNF750 downregulation, which potentiates ESCC metastasis. Thus, our finding revealed a TRIM29-STAT3-ZNF750 signaling axis in the ESCC progression, which is summarized in Fig. 8I.

In summary, this study explores the oncosuppressive function of TRIM29 in ESCC and reveals that TRIM29 downregulation due to hypermethylation of its promoter, positively regulates tumor suppresser gene ZNF750 via modulating IL6/STAT3 signaling pathway. The aberrant methylation status and expression of TRIM29 in precancerous lesions indicate its possible diagnostic and predictive value.

## Materials and methods

### Datasets from TCGA ESCA

RNA sequencing and clinical data in TCGA ESCA were obtained from TCGA (The Cancer Genome Atlas). TPM (Transcript per million) was calculated, and log_2_(X + 1) transformed. In addition, GEPIA (http://gepia2.cancer-pku.cn) was used to explore the differential expression of TRIM29 between tumor and normal tissues across 33 TCGA cancer types.

The methylation levels (β value) of 12 CpG sites in the promoter region of TRIM29, including cg11466837, cg00437969, cg13907859, cg24593464, cg24611264, cg09977361, cg17436370, cg13625403, cg13285004, cg17971587, cg12201660, and cg20655548, were extracted from TCGA DNA methylation dataset (HumanMethylation450K), which was taken from UCSC XENA (https://xenabrowser.net/). The methylation level of TRIM29 promoter was calculated as the mean β value of these 12 CpG sites.

### Datasets from other platforms

Twenty-six esophageal cancer datasets from GEO (Gene Expression Omnibus) were used in this study, including GSE21293 [59], GSE36223 [60], GSE39491 [61], GSE34619 [62], GSE26886 [63], GSE13898 [64], GSE22954 [65], GSE5364 [66], GSE92396 [67], GSE74553 [68], GSE32424 [69], GSE17351 [70], GSE75241 [71], GSE77861 [72], GSE119436 [73], GSE70409 [74], GSE20347 [75], GSE23400 [76], GSE37200 [77], GSE37201 [77], GSE47404 [78], GSE19417 [79], GSE26784 [80], GSE52826 [81], GSE81334 [82], and GSE104707 [83]. The mRNA sequencing data, methylation data, and clinicopathological information of these datasets were directly downloaded from GEO platform. The mRNA sequencing data and clinicopathological information of E-MTAB-4054 dataset were obtained from Arrayexpress [84]. The Liu2021 protein dataset was obtained from the supplementary material of a recently published paper [85]. The clinicopathological characteristics of the datasets used were summarized in Table S4.

### Gene set enrichment analysis

Firstly, we calculated the correlation coefficients of TRIM29 level with all other genes by Spearman’s correlation analyses and obtained a pre-ranked list. Then, the pre-ranked list was imported into GSEA software (version 4.1.0) for analysis with “h.all.v7.3.symbols.gmt”.

### Survival analysis

Survival analyses, including overall survival (OS), disease-specific survival (DSS), disease-free interval (DFI), progression-free interval (PFI), and disease-free survival (DFS), were ascertained using Kaplan–Meier methodology and analyzed by log-rank test with R packages “survminer” and “survival”. The patients were dichotomized into high and low groups based on an optimal cutoff value, which was determined with “survminer”.

### Cell culture and lentivirus transfection

Three ESCC cell lines (KYSE30, KYSE410, and KYSE450) were gifted by Professor Zhan QiMin (Peking University Cancer Hospital & Institute). All cell lines were genetically authenticated using STR profiling by Genesky Biotechnologies, Inc. The cells were cultured in RPMI1640 + 10% FBS (GIBCO).

The empty lentivector expressing GFP alone and recombinant lentivirus expressing TRIM29 were purchased from HanBio (Shanghai, China). The lentivirus encoding shRNA targeting TRIM29 was purchased from GenePharma (Shanghai, China). The shRNA sequences used were as follows: 5′- GAAGAGCTCCATCGTCTTGCCAcgaaTGGCAAGACGATGGAGCTCTTC -3′ and 5′-GCGACCCATCATCCAGTTTGTcgaaACAAACTGGATGATGGGTCGC -3′.

Full-length ZNF750 cDNA was amplified by PCR and cloned into pSIN-EF2-3×Flag for lentivirus production. HEK293T cells were transfected with pSIN-3×Flag-ZNF750 constructs, together with psPAX.2 and pMD2.G, using Attractene Transfection Reagent (Qiagen). The lentivirus-containing supernatant was collected and filtered 48 hours after transfection. To establish stable transfecting cell lines, cells were infected with lentivirus suspension (with 8 μg/ml polybrene) and then incubated with 2 μg/ml puromycin for 2 weeks.

### Patients and samples

Tumor samples and paired paracancerous tissues were obtained from four ESCC patients, which were confirmed by pathology. The surgeries of these patients were done at Taixing People’s Hospital. The research was approved by Human Research Ethics Committee of Anhui Medical University (permission number: 20210521).

### Western blot

The assay was performed as previously described [86]. The primary antibodies: TRIM29 (CST #50292), ZNF750 (ProteinTech #21752-1-AP), STAT3 (Abclonal #A16975), Phospho-Stat3 (Tyr705) (CST #9145), p21 (ProteinTech #10355-1-AP), KLF4 (ProteinTech #11880-1-AP), KRT5 (ProteinTech #66727-1-Ig), MMP2 (ProteinTech #10373-2-AP), MMP9 (ProteinTech #10375-2-AP), E-cadherin (ProteinTech #20874-1-AP), N-cadherin (ProteinTech #66219-1-Ig), Vimentin (ProteinTech #10366-1-AP), Snail (CST #3895), Cyclin D (ProteinTech #60186-1-Ig), β-catenin (CST #8480), and GAPDH (ProteinTech #60004-1-Ig). The secondary antibodies: anti‑rabbit IgG (ProteinTech #SA00001‑2) and anti‑mouse IgG (ProteinTech #SA00001‑1). Primary antibodies were diluted at a ratio of 1:1000 and incubated overnight at 4 °C. The secondary antibody was diluted at a ratio of 1:5000 and incubated for 1.5 hours at 4 °C at room temperature. The blots were visualized using the SuperSignal West Pico PLUS (Thermo Scientific) on the Tanon4600 Automatic chemiluminescence image analysis system (Tanon, Shanghai, China). All assays were performed in triplicate.

### Detection of DNA methylation level by bisulfite sequencing PCR

bisulfite sequencing PCR (BSP) analysis was performed to detect the differential methylation level of TRIM29 promoter in ESCC cell lines. The total genomic DNA of ESCC cell lines, including HEEC, KYSE30, KYSE410, and KYSE450, was extracted by DNAzol kit (Invitrogen, Carlsbad, CA, USA), then qualified and quantified by a NanoPhotometer (IMPLEN). Next, the genomic DNA was bisulfite converted using the EZ DNA Methylation–Gold Kit (ZYMO Research, D5006). The upstream CpG island of TRIM29 was amplified using the primers as follows: forward, 5′-TTTTTAGGTGGGGTTTGAGAT-3′ and reverse, 5′-AAAACTACCAAAAAACCACCC-3′. The amplified products were then cloned into T-vector and sequenced. Five different clones of each cell line were sequenced.

### qRT–PCR

The assays were performed as previously described [86]. The sequences of the primers: TRIM29 forward, GACGACCTGCTCAATGTATGC, and reverse, GTTGTTCACATAGCGATGGTCA; GAPDH forward, GGAGCGAGATCCCTCCAAAAT, and reverse, GGCTGTTGTCATACTTCTCATGG. All the experiments were performed in triplicate.

### Demethylating treatment

To investigate the effects of methylation on TRIM29 expression, KYSE30 cells were treated with 50 μM 5-aza-dC (Sigma-Aldrich, A3656) for three days with a daily exchange of culture medium. Cells were then collected and subjected to western blotting.

### JASPAR analysis

The ZNF750-binding sites in the KRT5 promoter region were predicted using JASPAR database. The sequence of KRT5 promoter region, 2000bp upstream and 100 bp downstream of KRT5 TSS (transcription start site), was obtained from NCBI, and then imported to JASPAR for ZNF750 motif binding analysis (Relative profile score threshold = 80%).

### ChIP-sequencing analysis

We used published ChIP-sequencing data from GEO for H3K4ME3 (GSE28332:GSM1303758 and GSE35583:GSM945286) [87, 88] and ZNF750 (GSE57702:GSM1387027) [12]. These ChIP-sequencing data were analyzed using Cistrome [89] and visualized with UCSC Genome Browser on human (GRCh38/hg38).

### Cell proliferation and colony formation assay

For the cell proliferation assays, the cells were cultured with 200ul medium/well in 96-well plates for four days. At the indicated intervals (1, 2, 3, 4, and 5 days), relative cell number was measured with a microplate reader after incubating with medium containing 10 μl of CCK8 (APExBIO #K1018) for 2 h. All assays were repeated at least three times.

For the colony formation assays, equal numbers of cells in the same groups (KYESE30 control vs. KYSE30 TRIM29-OE; KYSE410 control vs. KYSE410 TRIM29-shRNA; KYSE450 control vs. KYSE450 TRIM29-shRNA) were seeded in six-well plates and grown for 7–10 days. Then, the cell colonies were fixed with methanol, stained with crystal violet solution (0.5%), and counted.

### Flow cytometry analysis of cell cycle

Cells were trypsinized, washed twice with PBS, fixed in 70% ethanol, and stored at –20 °C overnight. Then, the cells were washed twice with PBS and incubated with a staining solution containing 0.1% RNaseA (100 µg/ml, Beyotime), 0.2% TritonX-100, and 1% propidium iodide staining solution (50 µg/ml) on ice for 30 min in the dark. The samples were analyzed using a Fortessa flow cytometer (BD Biosciences, NJ, USA), and the percentages of G1, S, and G2/M phase cells were calculated with FlowJo software. All experiments were performed in triplicate.

### Wound healing and invasion assays

The ability of cell migration was examined via scratch wound healing as previously described [86]. Briefly, the wound healing was made when the cells were grown to 90% confluence and then detected at the time point as mentioned in the respective figures. The ability of cell invasion was determined using a Boyden chamber assay using transwell chambers (8 μm pores, Corning) pre-coated with Matrigel (BD Biosciences). After being resuspended in serum-free medium, cells were plated in the upper compartment of 24-well Transwell plates (5 × 10^4^ cells/well) and incubated for 48 h, while the lower compartment was filled with growth medium. The inserts were fixed in 4% PFA for 15 min after removing cells from their top sides, and then stained with crystal violet solution. Cells on the lower sides of the membrane were counted. The assays were repeated three times.

### In vivo lung metastasis assay

The tail vein-lung metastasis mouse model was used to determine the ESCC cell’s ability to metastasize in vivo. Equal amount of KYSE30-control and KYSE30 TRIM29-overexpression cells (2 × 10^6^ cells/mouse) suspended in 250 μl normal saline were injected intravenously through the tail vein of the SCID/Beige mice, respectively. Mice were randomly grouped. Each group included five mice. Twelve weeks later, mice were killed by cervical dislocation, and their lungs were dissected and fixed in Bouin’s solution (Sigma-Aldrich, #HT10132) for 24 h at room temperature (RT). Then, the lungs were washed with PBS, metastatic colonies on the lung surface were counted, and the tumor lesions within the lungs were confirmed by H&E staining. The experiments were approved by the Ethics Committees of Anhui Medical University (permission number: LLSC20212790).

### Hematoxylin and eosin staining

After being fixed in Bouin’s solution for 24 h at RT, the lung specimens of mice were then embedded in paraffin, sectioned at 3 μm, sequentially rehydrated, and stained with H&E.

### Statistical analysis

Data are presented as mean ± standard deviation. Comparisons between the two groups were conducted using two-sided Student’s *t*-tests for normally distributed data or Mann–Whitney test for non-normally distributed data. Spearman’s correlation was used for all correlation analyses. The independent experiments were repeated a minimum of three times. The statistical significance level was set at *p* < 0.05. The analyses were conducted in GraphPad (v9.3.0) or R (v4.1.1). ∗ (*p* < 0.05), ∗∗ (*p* < 0.01), ∗∗∗ (*p* < 0.001), ∗∗∗∗ (*p* < 0.0001).

## Supplementary information

Supplementary figure legends

Figure S1

Figure S2

Figure S3

Figure S4

Table S1

Table S2

Table S3

Table S4

Original Data File

## Data Availability

The data used and/or analyzed during the current study are available from the corresponding author on reasonable request.
