# Supplementary material for: TRIM29 hypermethylation drives esophageal cancer progression via suppression of ZNF750
Source: Cell Death Discov. 2023 Jun 26;9:191. doi: 10.1038/s41420-023-01491-1 (PMC10293201; doi:10.1038/s41420-023-01491-1)
Supplement: Supplementary file 1 — Supplementary figure legends [file 41420_2023_1491_MOESM1_ESM.docx]

**Figure S1. ZNF750 and TRIM29 expression in GTEx.** (A&B) Violins show the expression of ZNF750 and TRIM29 across 54 types of normal tissues in GTEx Portal. (C-E) Scatterplot showing the positive correlation between ZNF750 and TRIM29 in normal tissues of skin (sun exposed), skin (not sun exposed), and esophagus mucosa.

**Figure S2. TRIM29 mutation analysis in TCGA ESCA.**

**Figure S3.** Scatterplot showing correlation between TRIM29 mRNA expression and β-value of 12 CpG sites, including cg11466837, cg00437969, cg13907859, cg24593464, cg24611264, cg09977361, cg17436370, cg13625403, cg13285004, cg17971587, cg12201660, and cg20655548, in TRIM29 promoter in TCGA ESCA.

**Figure S4. High methylation levels of CpG sites in TRIM29 promoter predict poor prognosis of ESCA patients.** (A) Kaplan-Meier analysis of OS according to β-value of cg00437969 in TCGA ESCA. (B) Kaplan-Meier analysis of OS according to β-value of cg20655548 in TCGA ESCA. (C) Kaplan-Meier analysis of DSS according to β-value of cg00437969 in TCGA ESCA. (D) Kaplan-Meier analysis of DSS according to β-value of cg20655548 in TCGA ESCA.
