# Supplementary figures and images for: TRIM29 hypermethylation drives esophageal cancer progression via suppression of ZNF750
Source: Cell Death Discov. 2023 Jun 26;9:191. doi: 10.1038/s41420-023-01491-1 (PMC10293201; doi:10.1038/s41420-023-01491-1)

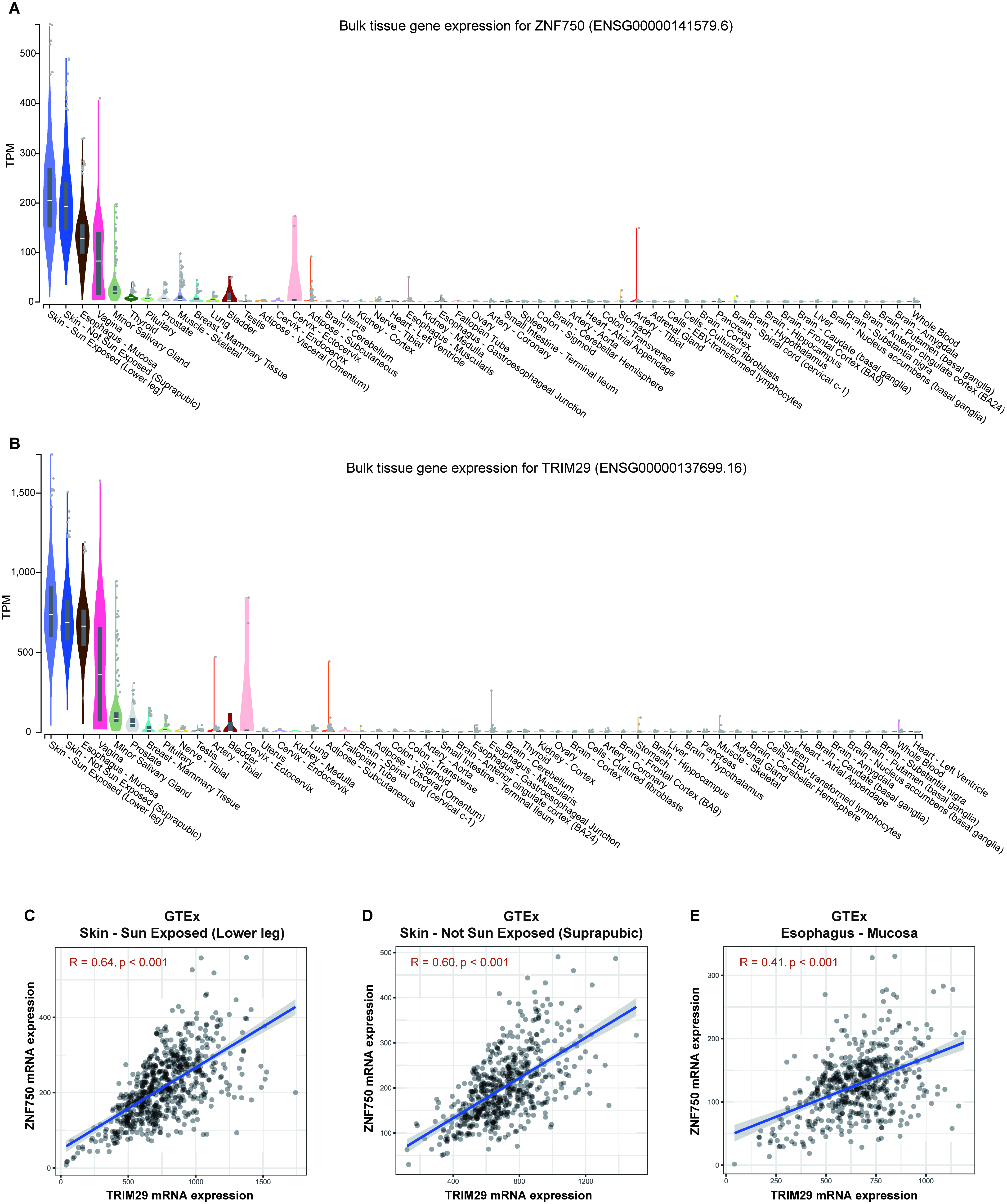

Supplement: Supplementary file 2 — Figure S1 [file 41420_2023_1491_MOESM2_ESM.tif]

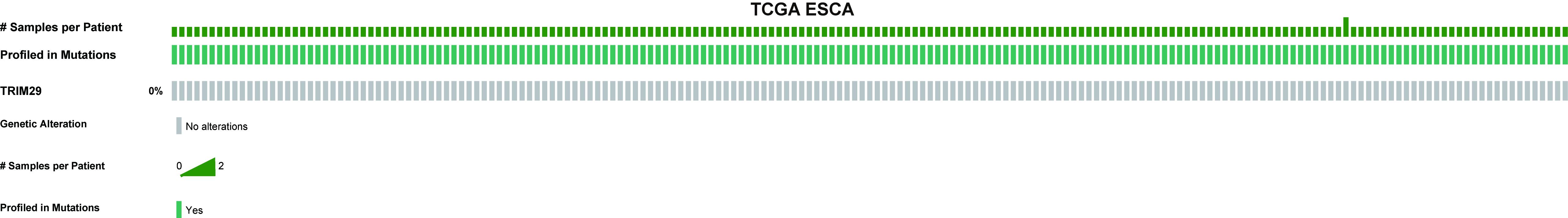

Supplement: Supplementary file 3 — Figure S2 [file 41420_2023_1491_MOESM3_ESM.tif]

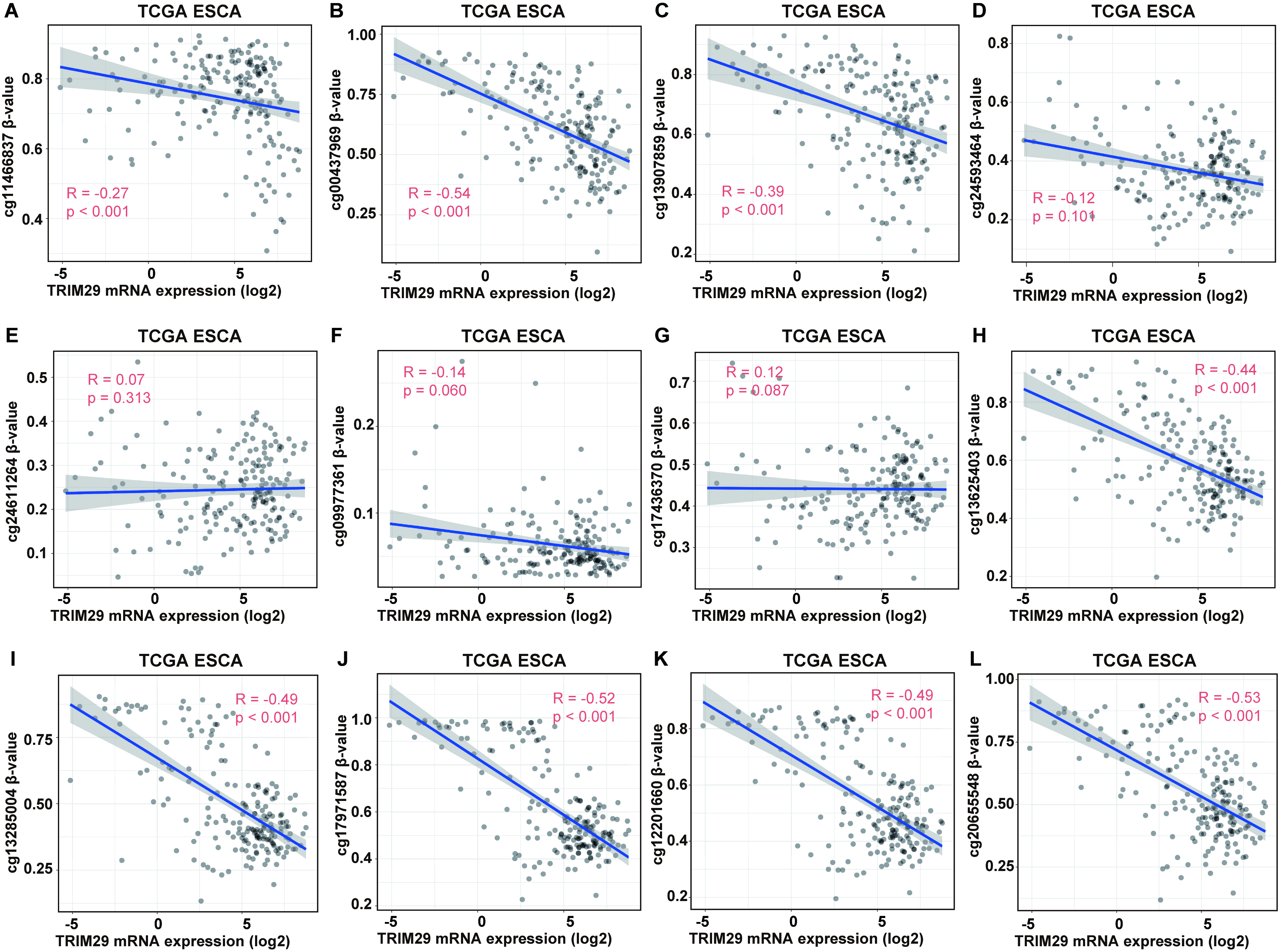

Supplement: Supplementary file 4 — Figure S3 [file 41420_2023_1491_MOESM4_ESM.tif]

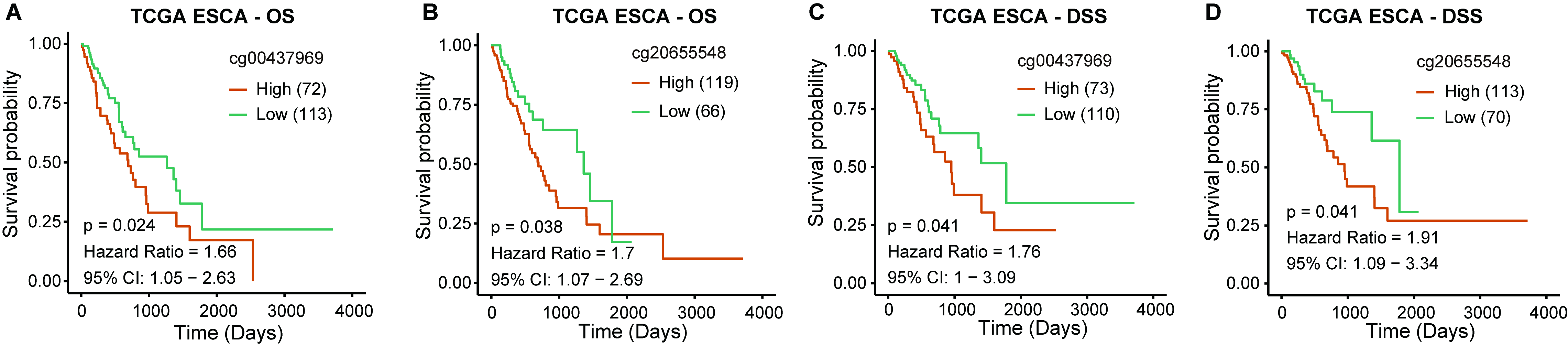

Supplement: Supplementary file 5 — Figure S4 [file 41420_2023_1491_MOESM5_ESM.tif]
