# Supplementary material for: TRIM29 hypermethylation drives esophageal cancer progression via suppression of ZNF750
Source: Cell Death Discov. 2023 Jun 26;9:191. doi: 10.1038/s41420-023-01491-1 (PMC10293201; doi:10.1038/s41420-023-01491-1)
Supplement: Supplementary file 6 — Table S1 [file 41420_2023_1491_MOESM6_ESM.docx]

**Table S1. Gene Ontology enrichment analysis of the down-regulated genes in invasive ESCC samples of GSE21293 dataset.**

| ID | Description | GeneRatio | pvalue | p.adjust |
| --- | --- | --- | --- | --- |
| GO:0008544 | epidermis development | 48/326 | 2.73E-23 | 8.48E-20 |
| GO:0070268 | cornification | 26/326 | 1.05E-21 | 1.63E-18 |
| GO:0043588 | skin development | 43/326 | 1.25E-20 | 1.30E-17 |
| GO:0030216 | keratinocyte differentiation | 37/326 | 2.00E-20 | 1.56E-17 |
| GO:0009913 | epidermal cell differentiation | 39/326 | 5.77E-20 | 3.59E-17 |
| GO:0031424 | keratinization | 29/326 | 1.02E-16 | 5.26E-14 |
| GO:0061436 | establishment of skin barrier | 9/326 | 1.28E-10 | 5.69E-08 |
| GO:0033561 | regulation of water loss via skin | 9/326 | 4.67E-10 | 1.82E-07 |
| GO:0050891 | multicellular organismal water homeostasis | 11/326 | 3.73E-08 | 1.29E-05 |
| GO:0030104 | water homeostasis | 11/326 | 9.23E-08 | 2.87E-05 |
| GO:0018149 | peptide cross-linking | 8/326 | 1.33E-07 | 3.76E-05 |
| GO:0010466 | negative regulation of peptidase activity | 18/326 | 1.48E-07 | 3.84E-05 |
| GO:0043312 | neutrophil degranulation | 27/326 | 2.84E-07 | 6.79E-05 |
| GO:0002283 | neutrophil activation involved in immune response | 27/326 | 3.21E-07 | 7.12E-05 |
| GO:0002446 | neutrophil mediated immunity | 27/326 | 4.97E-07 | 9.65E-05 |
| GO:0042119 | neutrophil activation | 27/326 | 4.97E-07 | 9.65E-05 |
| GO:0051346 | negative regulation of hydrolase activity | 23/326 | 2.92E-06 | 0.000535197 |
| GO:0019730 | antimicrobial humoral response | 11/326 | 3.66E-06 | 0.000632219 |
| GO:1901568 | fatty acid derivative metabolic process | 13/326 | 4.96E-06 | 0.000811412 |
| GO:0010951 | negative regulation of endopeptidase activity | 15/326 | 7.37E-06 | 0.001147138 |
| GO:0045861 | negative regulation of proteolysis | 19/326 | 8.25E-06 | 0.001222257 |
| GO:0098542 | defense response to other organism | 24/326 | 8.67E-06 | 0.001226101 |
| GO:0033559 | unsaturated fatty acid metabolic process | 10/326 | 1.18E-05 | 0.001595431 |
| GO:0045682 | regulation of epidermis development | 9/326 | 1.62E-05 | 0.00209371 |
| GO:0006690 | icosanoid metabolic process | 10/326 | 1.68E-05 | 0.00209371 |
| GO:0048871 | multicellular organismal homeostasis | 23/326 | 1.95E-05 | 0.002333577 |
| GO:0042742 | defense response to bacterium | 15/326 | 4.53E-05 | 0.005221062 |
| GO:0061844 | antimicrobial humoral immune response mediated by antimicrobial peptide | 7/326 | 5.95E-05 | 0.006605633 |
| GO:0016266 | O-glycan processing | 7/326 | 0.000104692 | 0.011230957 |
| GO:0052547 | regulation of peptidase activity | 19/326 | 0.000135067 | 0.014006474 |
| GO:0006959 | humoral immune response | 15/326 | 0.000171665 | 0.017227414 |
| GO:0006805 | xenobiotic metabolic process | 9/326 | 0.000181854 | 0.017679609 |
| GO:0001676 | long-chain fatty acid metabolic process | 8/326 | 0.000316193 | 0.029808333 |
| GO:0045684 | positive regulation of epidermis development | 5/326 | 0.000523163 | 0.047869433 |
