# Supplementary material for: TRIM29 hypermethylation drives esophageal cancer progression via suppression of ZNF750
Source: Cell Death Discov. 2023 Jun 26;9:191. doi: 10.1038/s41420-023-01491-1 (PMC10293201; doi:10.1038/s41420-023-01491-1)
Supplement: Supplementary file 8 — Table S3 [file 41420_2023_1491_MOESM8_ESM.docx]

**Table S3. List of common HALLMARKs significantly correlated with TRIM29 expression in TCGA ESCC and GSE21293**


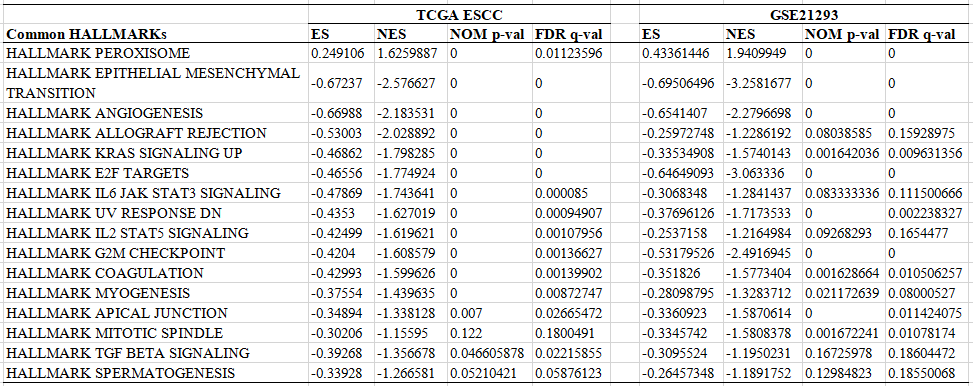


**ES:** Enrichment Score

**NES:** Normalized Enrichment Score

**NOM p-val:** normalized p-value

**FDR:** False Discovery Rate
