# Supplementary material for: TRIM29 hypermethylation drives esophageal cancer progression via suppression of ZNF750
Source: Cell Death Discov. 2023 Jun 26;9:191. doi: 10.1038/s41420-023-01491-1 (PMC10293201; doi:10.1038/s41420-023-01491-1)
Supplement: Supplementary file 9 — Table S4 [file 41420_2023_1491_MOESM9_ESM.docx]

**Table S4. Clinicopathological features of used datasets**

**TCGA ESCA**

| **sampleID** | **number of lymphnodes positive by he** | **grade** | **M stage** | **N stage** | **T stage** | **Stage** |
| --- | --- | --- | --- | --- | --- | --- |
| TCGA-2H-A9GF-01 | 7 | G3 | M0 | N1 | T3 | Stage III |
| TCGA-2H-A9GG-01 | 4 | G2 | M0 | N1 | T3 | Stage III |
| TCGA-2H-A9GH-01 | 1 | G2 | M0 | N1 | T1 | Stage IIB |
| TCGA-2H-A9GI-01 | 4 | G2 | M0 | N1 | T3 | Stage III |
| TCGA-2H-A9GJ-01 | 0 | G2 | M0 | N0 | T1 | Stage I |
| TCGA-2H-A9GK-01 | 3 | G3 | M0 | N1 | T3 | Stage III |
| TCGA-2H-A9GL-01 | 2 | G3 | M0 | N1 | T3 | Stage III |
| TCGA-2H-A9GM-01 | 1 | G2 | M0 | N1 | T1 | Stage IIB |
| TCGA-2H-A9GN-01 | 5 | G3 | M0 | N1 | T3 | Stage III |
| TCGA-2H-A9GO-01 | 9 | G3 | M1a | N1 | T3 | Stage IVA |
| TCGA-2H-A9GQ-01 | 2 | G3 | M0 | N1 | T3 | Stage III |
| TCGA-2H-A9GR-01 | 0 | G2 | M0 | N0 | T3 | Stage IIA |
| TCGA-IC-A6RE-01 | 2 | G2 | M0 | N1 | T1 | Stage IIB |
| TCGA-IC-A6RE-11 | 2 | G2 | M0 | N1 | T1 | Stage IIB |
| TCGA-IC-A6RF-01 | 0 | GX | M0 | N0 | T1 | Stage IA |
| TCGA-IC-A6RF-11 | 0 | GX | M0 | N0 | T1 | Stage IA |
| TCGA-IG-A3I8-01 | 0 | G2 | M0 | N0 | T3 | Stage IIA |
| TCGA-IG-A3I8-11 | 0 | G2 | M0 | N0 | T3 | Stage IIA |
| TCGA-IG-A3QL-01 | 0 | G2 | M0 | N0 | T2 | Stage IIA |
| TCGA-IG-A3Y9-01 | 0 | G2 | M0 | N0 | T4 | Stage IIIA |
| TCGA-IG-A3YA-01 | 0 | G2 | M0 | N0 | T4 | Stage IIIA |
| TCGA-IG-A3YB-01 | 1 | G2 | M0 | N1 | T3 | Stage IIIA |
| TCGA-IG-A3YC-01 | 1 | G2 | M0 | N1 | T3 | Stage IIIA |
| TCGA-IG-A4P3-01 | 2 | G2 | M0 | N1 | T2 | Stage IIB |
| TCGA-IG-A4QS-01 | 6 | G3 | M0 | N2 | T3 | Stage IIIB |
| TCGA-IG-A4QT-01 | 0 | G2 | M0 | N0 | T3 | Stage IIA |
| TCGA-IG-A50L-01 | 1 | G2 | M0 | N1 | T3 | Stage IIIA |
| TCGA-IG-A51D-01 | 1 | G2 | M0 | N1 | T1 | Stage IIB |
| TCGA-IG-A5B8-01 | 0 | G1 | M0 | N0 | T3 | Stage IB |
| TCGA-IG-A5S3-01 | 0 | G2 | M0 | N0 | T3 | Stage IIB |
| TCGA-IG-A625-01 | 5 | G2 | M0 | N2 | T3 | Stage IIIB |
| TCGA-IG-A6QS-01 | 2 | G1 | M0 | N1 | T2 | Stage IIB |
| TCGA-IG-A7DP-01 |  | G2 | MX | NX | T4a | Stage IIIA |
| TCGA-IG-A8O2-01 | 6 | G3 | M0 | N2 | T3 | Stage IIIB |
| TCGA-IG-A97H-01 |  | G3 | M0 | NX | T3 | Stage IIA |
| TCGA-IG-A97I-01 | 0 | G2 | M0 | N0 | T2 | Stage IIA |
| TCGA-JY-A6F8-01 | 0 | G1 | M0 | N0 | T1 | Stage I |
| TCGA-JY-A6FA-01 | 4 | G2 | M0 | N1 | T2 | Stage IIB |
| TCGA-JY-A6FB-01 | 0 | G2 | M0 | N0 | T1 | Stage I |
| TCGA-JY-A6FD-01 | 0 | G1 | M0 | N0 | T3 | Stage IIA |
| TCGA-JY-A6FE-01 | 8 | G3 | M0 | N1 | T3 | Stage III |
| TCGA-JY-A6FG-01 | 5 | G2 | M0 | N1 | T3 | Stage III |
| TCGA-JY-A6FH-01 | 2 | G2 | M0 | N1 | T2 | Stage IIB |
| TCGA-JY-A938-01 | 0 | G3 | M0 | N0 | T3 | Stage IIB |
| TCGA-JY-A939-01 | 1 | G3 | M0 | N1 | T1 | Stage IIB |
| TCGA-JY-A93C-01 | 3 | G3 | M0 | N2 | T3 | Stage IIIB |
| TCGA-JY-A93D-01 | 14 | G3 | M0 | N3 | T2 | Stage IIIC |
| TCGA-JY-A93E-01 | 1 | G3 | M0 | N1 | T3 | Stage IIIA |
| TCGA-JY-A93F-01 | 0 | G1 | M0 | N0 | T2 | Stage IB |
| TCGA-KH-A6WC-01 |  | GX | M0 | N0 | T1 | Stage IA |
| TCGA-L5-A43C-01 | 12 | GX |  |  |  |  |
| TCGA-L5-A43C-11 | 12 | GX |  |  |  |  |
| TCGA-L5-A43E-01 | 0 | GX | M0 | N0 | T1 | Stage I |
| TCGA-L5-A43H-01 | 2 | GX | M0 | N1 | T3 | Stage III |
| TCGA-L5-A43I-01 | 2 | GX | MX | N1 | T3 | Stage IIIA |
| TCGA-L5-A43J-01 | 0 | G3 | MX | N0 | T3 | Stage IIB |
| TCGA-L5-A43M-01 | 4 | GX | MX | N2 | T3 |  |
| TCGA-L5-A4OE-01 | 3 | G2 |  | N2 | T3 | Stage IIIB |
| TCGA-L5-A4OE-11 | 3 | G2 |  | N2 | T3 | Stage IIIB |
| TCGA-L5-A4OF-01 | 1 | G3 |  | N1 | T1 | Stage IIB |
| TCGA-L5-A4OF-11 | 1 | G3 |  | N1 | T1 | Stage IIB |
| TCGA-L5-A4OG-01 | 0 | G1 | M0 | N0 | T1 | Stage I |
| TCGA-L5-A4OG-11 | 0 | G1 | M0 | N0 | T1 | Stage I |
| TCGA-L5-A4OH-01 | 0 | GX | M0 | N0 | T1 | Stage I |
| TCGA-L5-A4OH-11 | 0 | GX | M0 | N0 | T1 | Stage I |
| TCGA-L5-A4OI-01 | 8 | G2 | MX | N3 | T3 | Stage IIIC |
| TCGA-L5-A4OI-11 | 8 | G2 | MX | N3 | T3 | Stage IIIC |
| TCGA-L5-A4OJ-01 | 0 | G3 | M0 | N0 | T1 | Stage I |
| TCGA-L5-A4OJ-11 | 0 | G3 | M0 | N0 | T1 | Stage I |
| TCGA-L5-A4OM-01 | 0 | GX |  | N0 | T1 | Stage IA |
| TCGA-L5-A4OM-11 | 0 | GX |  | N0 | T1 | Stage IA |
| TCGA-L5-A4ON-01 | 1 | GX |  | N1 | T1 | Stage IIB |
| TCGA-L5-A4ON-11 | 1 | GX |  | N1 | T1 | Stage IIB |
| TCGA-L5-A4OO-01 | 21 | G2 | M0 | N3 | T3 | Stage IIIC |
| TCGA-L5-A4OO-11 | 21 | G2 | M0 | N3 | T3 | Stage IIIC |
| TCGA-L5-A4OP-01 | 0 | G2 | MX | N0 | T1 | Stage IA |
| TCGA-L5-A4OP-11 | 0 | G2 | MX | N0 | T1 | Stage IA |
| TCGA-L5-A4OQ-01 | 6 | GX | MX | N2 | T2 | Stage IIIA |
| TCGA-L5-A4OQ-11 | 6 | GX | MX | N2 | T2 | Stage IIIA |
| TCGA-L5-A4OR-01 | 0 | GX | MX | N0 | T1 | Stage IA |
| TCGA-L5-A4OR-11 | 0 | GX | MX | N0 | T1 | Stage IA |
| TCGA-L5-A4OS-01 | 1 | G3 | M0 | N1 | T2 | Stage IIB |
| TCGA-L5-A4OT-01 | 6 | GX | M1a | N1 | T3 | Stage IV |
| TCGA-L5-A4OU-01 | 0 | GX | M0 | N0 | T3 | Stage IIA |
| TCGA-L5-A4OW-01 | 2 | GX | M0 | N1 | T2 | Stage IIB |
| TCGA-L5-A4OX-01 | 3 | GX | M0 | N1 | T2 | Stage IIB |
| TCGA-L5-A88S-01 | 0 | GX | MX | N0 | T3 | Stage IB |
| TCGA-L5-A88T-01 | 2 | GX | M0 | N1 | T2 | Stage IIB |
| TCGA-L5-A88V-01 | 1 | GX | MX | N1 | T3 | Stage III |
| TCGA-L5-A88W-01 | 0 | GX | MX | N0 | T3 | Stage IIA |
| TCGA-L5-A88Y-01 | 0 | GX | MX | N0 | T1 |  |
| TCGA-L5-A88Z-01 | 2 | GX | M0 | N1 | T1 | Stage IIA |
| TCGA-L5-A891-01 | 2 | GX | M0 | N1 | T3 |  |
| TCGA-L5-A893-01 | 0 | GX | M0 | N0 | T1 | Stage I |
| TCGA-L5-A8NE-01 | 1 | GX | M0 | N1 | T2 | Stage IIB |
| TCGA-L5-A8NF-01 | 1 | GX | M1a | N0 | T1 | Stage IVA |
| TCGA-L5-A8NG-01 | 1 | GX | M0 | N1 | T3 | Stage III |
| TCGA-L5-A8NH-01 | 1 | GX | M1 | N0 | T1 | Stage IV |
| TCGA-L5-A8NI-01 | 4 | G3 | M0 | N1 | T3 | Stage III |
| TCGA-L5-A8NJ-01 | 4 | GX | M0 | N1 | T3 | Stage III |
| TCGA-L5-A8NK-01 | 0 | G1 | M0 | N0 | T3 | Stage IIA |
| TCGA-L5-A8NL-01 | 4 | GX | M0 | N1 | T3 | Stage III |
| TCGA-L5-A8NM-01 | 1 | GX | M0 | N1 | T2 | Stage IIB |
| TCGA-L5-A8NN-01 | 4 | GX | M0 | N1 | T3 | Stage III |
| TCGA-L5-A8NQ-01 | 0 | G3 | M0 | N0 | T2 | Stage IIA |
| TCGA-L5-A8NR-01 | 7 | GX | M0 | N1 | T3 | Stage III |
| TCGA-L5-A8NS-01 | 0 | GX | M0 | N0 | T3 | Stage IIB |
| TCGA-L5-A8NT-01 | 0 | GX | M0 | N0 | T3 | Stage IIB |
| TCGA-L5-A8NU-01 |  | G3 | M0 | N0 | T2 | Stage IIA |
| TCGA-L5-A8NV-01 | 0 | G3 | M0 | N0 | T3 | Stage IIA |
| TCGA-L5-A8NW-01 | 6 | GX | M0 | N1 | T2 | Stage IB |
| TCGA-L7-A56G-01 |  | G3 |  |  |  |  |
| TCGA-L7-A6VZ-01 | 15 | G2 | MX | N3 | T3 | Stage IIIC |
| TCGA-LN-A49K-01 |  | G2 | M0 | N0 | T3 | Stage IIA |
| TCGA-LN-A49L-01 |  | G2 | M0 | N0 | T2 | Stage IIA |
| TCGA-LN-A49M-01 |  | G1 | M0 | N0 | T2 | Stage IIA |
| TCGA-LN-A49N-01 | 1 | G2 | M0 | N1 | T2 | Stage IIB |
| TCGA-LN-A49O-01 |  | G3 | M0 | N0 | T3 | Stage IIA |
| TCGA-LN-A49P-01 |  | G2 | M0 | N0 | T3 | Stage IIA |
| TCGA-LN-A49R-01 | 1 | G3 | M0 | N1 | T3 | Stage III |
| TCGA-LN-A49S-01 |  | G2 | M0 | N0 | T3 | Stage IIA |
| TCGA-LN-A49U-01 |  | G1 | M0 | N0 | T3 | Stage IIA |
| TCGA-LN-A49V-01 |  | G3 | M0 | N0 | T3 | Stage IIA |
| TCGA-LN-A49W-01 | 2 | G3 | M0 | N1 | T3 | Stage III |
| TCGA-LN-A49X-01 |  | G2 | M0 | N0 | T3 | Stage IIA |
| TCGA-LN-A49Y-01 |  | G2 | M0 | N0 | T3 | Stage IIA |
| TCGA-LN-A4A1-01 |  | G3 | M0 | N0 | T3 | Stage IIA |
| TCGA-LN-A4A2-01 |  | G1 | M0 | N0 | T3 | Stage IIA |
| TCGA-LN-A4A3-01 | 2 | G2 | M0 | N1 | T3 | Stage III |
| TCGA-LN-A4A4-01 | 2 | GX | M0 | N1 | T3 | Stage III |
| TCGA-LN-A4A5-01 |  | G2 | M0 | N0 | T2 | Stage IIA |
| TCGA-LN-A4A6-01 |  | G2 | M0 | N0 | T2 | Stage II |
| TCGA-LN-A4A8-01 |  | G2 | M0 | N0 | T2 | Stage IIA |
| TCGA-LN-A4A9-01 |  | G1 | M0 | N0 | T2 | Stage IIA |
| TCGA-LN-A4MQ-01 | 2 | G3 | M0 | N1 | T3 | Stage III |
| TCGA-LN-A4MR-01 |  | G2 | M0 | N0 | T2 | Stage IIA |
| TCGA-LN-A5U5-01 | 5 | G2 | M1 | N1 | T3 | Stage IV |
| TCGA-LN-A5U6-01 | 1 | G2 | M0 | N1 | T2 | Stage IIB |
| TCGA-LN-A5U7-01 |  | G1 | M0 | N0 | T2 | Stage IIA |
| TCGA-LN-A7HV-01 |  | G1 | M0 | N0 | T2 | Stage IIA |
| TCGA-LN-A7HW-01 |  | G2 | M0 | N0 | T2 | Stage IIA |
| TCGA-LN-A7HX-01 |  | G2 | M0 | N0 | T2 | Stage IIA |
| TCGA-LN-A7HY-01 | 1 | G2 | M0 | N1 | T3 | Stage III |
| TCGA-LN-A7HZ-01 |  | G2 | M0 | N0 | T2 | Stage IIA |
| TCGA-LN-A8HZ-01 |  | G3 | M0 | N0 | T2 | Stage IIA |
| TCGA-LN-A8I0-01 | 0 | G2 | M0 | N0 | T2 | Stage IIA |
| TCGA-LN-A8I1-01 |  | G3 | M0 | N0 | T2 | Stage IIA |
| TCGA-LN-A9FO-01 |  | G2 | M0 | N0 | T2 | Stage IIA |
| TCGA-LN-A9FP-01 |  | G3 | M0 | N0 | T2 | Stage IIA |
| TCGA-LN-A9FQ-01 |  | G3 | M0 | N0 | T3 | Stage IIA |
| TCGA-LN-A9FR-01 | 1 | G2 | M0 | N1 | T2 | Stage IIB |
| TCGA-M9-A5M8-01 | 0 | G2 | M0 | N0 | T3 | Stage IIA |
| TCGA-Q9-A6FU-01 | 4 | G2 | M0 | N2 | T3 | Stage IIIB |
| TCGA-Q9-A6FW-01 | 3 | G2 | M0 | N2 | T3 | Stage IIIB |
| TCGA-R6-A6DN-01 |  | G3 |  |  |  |  |
| TCGA-R6-A6DQ-01 |  | G3 |  |  |  |  |
| TCGA-R6-A6KZ-01 |  | G3 |  |  |  |  |
| TCGA-R6-A6L4-01 |  | G2 |  |  |  |  |
| TCGA-R6-A6L6-01 |  | G3 |  |  |  |  |
| TCGA-R6-A6XG-01 |  | G2 |  |  |  |  |
| TCGA-R6-A6XQ-01 |  | G3 |  |  |  |  |
| TCGA-R6-A6Y0-01 |  | G2 |  |  |  |  |
| TCGA-R6-A6Y2-01 |  | G3 |  |  |  |  |
| TCGA-R6-A8W5-01 |  | G2 | M1a | N1 | T3 | Stage IVA |
| TCGA-R6-A8W8-01 |  | G2 |  |  |  |  |
| TCGA-R6-A8WC-01 |  | G2 |  |  |  |  |
| TCGA-R6-A8WG-01 |  | G3 |  |  |  |  |
| TCGA-RE-A7BO-01 | 2 | G3 | M0 | N1 | T1 | Stage IIB |
| TCGA-S8-A6BV-01 | 2 | G2 | MX | N1 | T3 | Stage IIIA |
| TCGA-S8-A6BW-01 | 0 | G1 | MX | N0 | T2 | Stage IB |
| TCGA-V5-A7RB-01 | 1 | GX | MX | N1 | T0 |  |
| TCGA-V5-A7RC-01 |  | GX |  |  |  |  |
| TCGA-V5-A7RC-06 |  | GX |  |  |  |  |
| TCGA-V5-A7RE-01 | 1 | G2 | M0 | N0 | T1 | Stage IB |
| TCGA-V5-A7RE-11 | 1 | G2 | M0 | N0 | T1 | Stage IB |
| TCGA-V5-AASV-01 |  | G3 | MX | N0 | T3 | Stage IIB |
| TCGA-V5-AASW-01 |  | G3 |  |  |  |  |
| TCGA-V5-AASX-01 |  | G2 |  |  |  |  |
| TCGA-V5-AASX-11 |  | G2 |  |  |  |  |
| TCGA-VR-A8EO-01 | 0 | G3 | M0 | N0 | T3 | Stage IIA |
| TCGA-VR-A8EP-01 | 4 | G3 | M0 | N2 | T3 | Stage IIIB |
| TCGA-VR-A8EQ-01 | 7 | G2 | M0 | N1 | T3 | Stage III |
| TCGA-VR-A8ER-01 | 3 | GX | M0 | N1 | T4 | Stage III |
| TCGA-VR-A8ET-01 | 0 | G2 | M0 | N0 | T2 | Stage IIA |
| TCGA-VR-A8EU-01 | 2 | G2 | M1 | N1 | T1 | Stage IV |
| TCGA-VR-A8EW-01 | 4 | G2 | M0 | N2 | T3 | Stage IIIB |
| TCGA-VR-A8EX-01 | 9 | G2 | M1a | N1 | T1 | Stage IVA |
| TCGA-VR-A8EY-01 | 0 | G1 | M0 | N0 | T3 | Stage IIA |
| TCGA-VR-A8EZ-01 | 7 | G2 | M0 | N3 | T3 | Stage IIIC |
| TCGA-VR-A8Q7-01 | 3 | G3 | M0 | N1 | T3 | Stage IIIA |
| TCGA-VR-AA4D-01 | 6 | G2 | M0 | N1 | T1 | Stage IIB |
| TCGA-VR-AA4G-01 | 1 | G2 | M0 | N2 | T2 | Stage IIIA |
| TCGA-VR-AA7B-01 | 9 | G2 | M1 | N3 | T3 | Stage IV |
| TCGA-VR-AA7D-01 | 7 | G2 | M0 | N3 | T3 | Stage IIIC |
| TCGA-VR-AA7I-01 | 0 | G1 | M0 | N0 | T4 | Stage III |
| TCGA-X8-AAAR-01 | 0 | G1 |  | N1 | T1 |  |
| TCGA-XP-A8T6-01 | 3 | G2 | M0 | N1 | T2 | Stage IIB |
| TCGA-XP-A8T7-01 | 0 | G2 | M0 | N0 | T2 | Stage IIA |
| TCGA-XP-A8T8-01 | 1 | G1 | M0 | N1 | T1 | Stage IIB |
| TCGA-Z6-A8JD-01 | 0 | G2 | M0 | N0 | T3 | Stage IIB |
| TCGA-Z6-A8JE-01 | 1 | G3 | M0 | N1 | T3 | Stage IIIA |
| TCGA-Z6-A9VB-01 |  | G2 | M0 | N1 | T3 | Stage IIIA |
| TCGA-Z6-AAPN-01 | 0 | G1 | M0 | N0 | T3 | Stage IIA |
| TCGA-ZR-A9CJ-01 | 12 | G3 | MX | N3 | T3 | Stage IIIC |

| **sampleID** | **OS** | **OS.time**  **(days)** | **DSS** | **DSS.time**  **(days)** | **DFI** | **DFI.time**  **(days)** | **PFI** | **PFI.time**  **(days)** |
| --- | --- | --- | --- | --- | --- | --- | --- | --- |
| TCGA-2H-A9GF-01 | 1 | 784 | 1 | 784 |  |  | 1 | 172 |
| TCGA-2H-A9GG-01 | 1 | 610 | 1 | 610 |  |  | 1 | 504 |
| TCGA-2H-A9GH-01 | 1 | 951 | 1 | 951 |  |  | 1 | 800 |
| TCGA-2H-A9GI-01 | 1 | 435 | 1 | 435 |  |  | 1 | 400 |
| TCGA-2H-A9GJ-01 | 1 | 1781 | 1 | 1781 |  |  | 1 | 1261 |
| TCGA-2H-A9GK-01 | 1 | 232 | 1 | 232 |  |  | 1 | 113 |
| TCGA-2H-A9GL-01 | 1 | 180 | 1 | 180 |  |  | 1 | 109 |
| TCGA-2H-A9GM-01 | 1 | 424 | 1 | 424 |  |  | 1 | 367 |
| TCGA-2H-A9GN-01 | 1 | 272 | 1 | 272 |  |  | 1 | 242 |
| TCGA-2H-A9GO-01 | 1 | 494 | 1 | 494 |  |  | 1 | 362 |
| TCGA-2H-A9GQ-01 | 1 | 128 | 1 | 128 |  |  | 1 | 112 |
| TCGA-2H-A9GR-01 | 1 | 987 | 1 | 987 |  |  | 1 | 987 |
| TCGA-IC-A6RE-01 | 0 | 234 | 0 | 234 | 0 | 234 | 0 | 234 |
| TCGA-IC-A6RE-11 | 0 | 234 | 0 | 234 | 0 | 234 | 0 | 234 |
| TCGA-IC-A6RF-01 | 0 | 477 | 0 | 477 | 1 | 293 | 1 | 293 |
| TCGA-IC-A6RF-11 | 0 | 477 | 0 | 477 | 1 | 293 | 1 | 293 |
| TCGA-IG-A3I8-01 | 0 | 1012 | 0 | 1012 | 0 | 1012 | 0 | 1012 |
| TCGA-IG-A3I8-11 | 0 | 1012 | 0 | 1012 | 0 | 1012 | 0 | 1012 |
| TCGA-IG-A3QL-01 | 0 | 1071 | 0 | 1071 | 0 | 1071 | 0 | 1071 |
| TCGA-IG-A3Y9-01 | 1 | 26 | 0 | 26 | 0 | 26 | 0 | 26 |
| TCGA-IG-A3YA-01 | 0 | 632 | 0 | 632 | 0 | 632 | 0 | 632 |
| TCGA-IG-A3YB-01 | 0 | 80 | 0 | 80 | 0 | 80 | 0 | 80 |
| TCGA-IG-A3YC-01 | 0 | 612 | 0 | 612 |  |  | 1 | 542 |
| TCGA-IG-A4P3-01 | 1 | 567 | 1 | 567 |  |  | 1 | 567 |
| TCGA-IG-A4QS-01 | 1 | 118 | 0 | 118 | 0 | 118 | 0 | 118 |
| TCGA-IG-A4QT-01 | 1 | 283 | 0 | 283 | 0 | 283 | 0 | 283 |
| TCGA-IG-A50L-01 | 0 | 16 | 0 | 16 |  |  | 0 | 16 |
| TCGA-IG-A51D-01 | 0 | 518 | 0 | 518 |  |  | 0 | 518 |
| TCGA-IG-A5B8-01 | 1 | 24 | 0 | 24 |  |  | 0 | 24 |
| TCGA-IG-A5S3-01 | 0 | 712 | 0 | 712 | 0 | 712 | 0 | 712 |
| TCGA-IG-A625-01 | 1 | 390 | 0 | 390 |  |  | 0 | 390 |
| TCGA-IG-A6QS-01 | 1 | 303 | 0 | 303 | 0 | 303 | 0 | 303 |
| TCGA-IG-A7DP-01 | 0 | 452 | 0 | 452 | 0 | 452 | 0 | 452 |
| TCGA-IG-A8O2-01 | 1 | 142 | 0 | 142 | 0 | 142 | 0 | 142 |
| TCGA-IG-A97H-01 | 0 | 441 | 0 | 441 | 0 | 441 | 0 | 441 |
| TCGA-IG-A97I-01 | 0 | 370 | 0 | 370 | 0 | 370 | 0 | 370 |
| TCGA-JY-A6F8-01 | 0 | 3714 | 0 | 3714 | 0 | 3714 | 0 | 3714 |
| TCGA-JY-A6FA-01 | 1 | 1361 | 1 | 1361 |  |  | 1 | 477 |
| TCGA-JY-A6FB-01 | 0 | 1837 | 0 | 1837 | 0 | 1837 | 0 | 1837 |
| TCGA-JY-A6FD-01 | 0 | 2069 | 0 | 2069 | 0 | 2069 | 0 | 2069 |
| TCGA-JY-A6FE-01 | 1 | 112 | 1 | 112 |  |  | 1 | 55 |
| TCGA-JY-A6FG-01 | 1 | 1263 | 0 | 1263 |  |  | 0 | 1263 |
| TCGA-JY-A6FH-01 | 0 | 1441 | 0 | 1441 |  |  | 1 | 749 |
| TCGA-JY-A938-01 | 0 | 1060 | 0 | 1060 | 1 | 493 | 1 | 493 |
| TCGA-JY-A939-01 | 0 | 660 | 0 | 660 |  |  | 0 | 660 |
| TCGA-JY-A93C-01 | 0 | 705 | 0 | 705 | 1 | 652 | 1 | 652 |
| TCGA-JY-A93D-01 | 1 | 960 | 1 | 960 | 1 | 732 | 1 | 732 |
| TCGA-JY-A93E-01 | 0 | 767 | 0 | 767 |  |  | 0 | 767 |
| TCGA-JY-A93F-01 | 0 | 731 | 0 | 731 | 0 | 731 | 0 | 731 |
| TCGA-KH-A6WC-01 | 0 | 191 | 0 | 191 | 0 | 191 | 0 | 191 |
| TCGA-L5-A43C-01 | 0 | 96 | 0 | 96 |  |  | 0 | 96 |
| TCGA-L5-A43C-11 | 0 | 96 | 0 | 96 |  |  | 0 | 96 |
| TCGA-L5-A43E-01 | 0 | 920 | 0 | 920 | 0 | 920 | 1 | 885 |
| TCGA-L5-A43H-01 | 1 | 9 | 1 | 9 |  |  | 1 | 9 |
| TCGA-L5-A43I-01 | 1 | 556 | 0 | 556 | 0 | 556 | 0 | 556 |
| TCGA-L5-A43J-01 | 1 | 131 | 1 | 131 |  |  | 1 | 98 |
| TCGA-L5-A43M-01 | 0 | 272 | 0 | 272 |  |  | 0 | 272 |
| TCGA-L5-A4OE-01 | 1 | 730 |  | 730 | 0 | 730 | 0 | 730 |
| TCGA-L5-A4OE-11 | 1 | 730 |  | 730 | 0 | 730 | 0 | 730 |
| TCGA-L5-A4OF-01 | 1 | 801 |  | 801 | 0 | 801 | 0 | 801 |
| TCGA-L5-A4OF-11 | 1 | 801 |  | 801 | 0 | 801 | 0 | 801 |
| TCGA-L5-A4OG-01 | 0 | 143 | 0 | 143 |  |  | 0 | 143 |
| TCGA-L5-A4OG-11 | 0 | 143 | 0 | 143 |  |  | 0 | 143 |
| TCGA-L5-A4OH-01 | 0 | 992 | 0 | 992 | 0 | 992 | 0 | 992 |
| TCGA-L5-A4OH-11 | 0 | 992 | 0 | 992 | 0 | 992 | 0 | 992 |
| TCGA-L5-A4OI-01 | 0 | 608 | 0 | 608 |  |  | 1 | 267 |
| TCGA-L5-A4OI-11 | 0 | 608 | 0 | 608 |  |  | 1 | 267 |
| TCGA-L5-A4OJ-01 | 0 | 639 | 0 | 639 | 0 | 639 | 0 | 639 |
| TCGA-L5-A4OJ-11 | 0 | 639 | 0 | 639 | 0 | 639 | 0 | 639 |
| TCGA-L5-A4OM-01 | 1 | 1458 | 0 | 1458 | 0 | 1458 | 0 | 1458 |
| TCGA-L5-A4OM-11 | 1 | 1458 | 0 | 1458 | 0 | 1458 | 0 | 1458 |
| TCGA-L5-A4ON-01 | 1 | 558 | 0 | 558 | 0 | 558 | 0 | 558 |
| TCGA-L5-A4ON-11 | 1 | 558 | 0 | 558 | 0 | 558 | 0 | 558 |
| TCGA-L5-A4OO-01 | 0 | 101 | 0 | 101 |  |  | 0 | 101 |
| TCGA-L5-A4OO-11 | 0 | 101 | 0 | 101 |  |  | 0 | 101 |
| TCGA-L5-A4OP-01 | 0 | 218 | 0 | 218 |  |  | 0 | 218 |
| TCGA-L5-A4OP-11 | 0 | 218 | 0 | 218 |  |  | 0 | 218 |
| TCGA-L5-A4OQ-01 | 1 | 42 | 1 | 42 |  |  | 1 | 29 |
| TCGA-L5-A4OQ-11 | 1 | 42 | 1 | 42 |  |  | 1 | 29 |
| TCGA-L5-A4OR-01 | 1 | 96 | 0 | 96 | 0 | 96 | 0 | 96 |
| TCGA-L5-A4OR-11 | 1 | 96 | 0 | 96 | 0 | 96 | 0 | 96 |
| TCGA-L5-A4OS-01 | 0 | 1782 | 0 | 1782 |  |  | 0 | 1782 |
| TCGA-L5-A4OT-01 | 1 | 149 | 1 | 149 |  |  | 1 | 149 |
| TCGA-L5-A4OU-01 | 0 | 882 | 0 | 882 |  |  | 0 | 882 |
| TCGA-L5-A4OW-01 | 1 | 217 | 0 | 217 | 0 | 217 | 0 | 217 |
| TCGA-L5-A4OX-01 | 1 | 226 | 0 | 226 | 0 | 226 | 0 | 226 |
| TCGA-L5-A88S-01 | 0 | 471 | 0 | 471 | 0 | 471 | 0 | 471 |
| TCGA-L5-A88T-01 | 0 | 694 | 0 | 694 | 0 | 694 | 0 | 694 |
| TCGA-L5-A88V-01 | 0 | 79 | 0 | 79 |  |  | 0 | 79 |
| TCGA-L5-A88W-01 | 1 | 764 | 1 | 764 |  |  | 1 | 315 |
| TCGA-L5-A88Y-01 | 0 | 11 | 0 | 11 |  |  | 0 | 11 |
| TCGA-L5-A88Z-01 | 0 | 225 | 0 | 225 |  |  | 0 | 225 |
| TCGA-L5-A891-01 | 0 | 114 | 0 | 114 |  |  | 0 | 114 |
| TCGA-L5-A893-01 | 0 | 92 | 0 | 92 |  |  | 0 | 92 |
| TCGA-L5-A8NE-01 | 0 | 1688 | 0 | 1688 |  |  | 0 | 1688 |
| TCGA-L5-A8NF-01 | 1 | 81 | 0 | 81 |  |  | 0 | 81 |
| TCGA-L5-A8NG-01 | 0 | 1094 | 0 | 1094 |  |  | 0 | 1094 |
| TCGA-L5-A8NH-01 | 1 | 393 | 0 | 393 |  |  | 0 | 393 |
| TCGA-L5-A8NI-01 | 1 | 410 | 1 | 410 |  |  | 1 | 401 |
| TCGA-L5-A8NJ-01 | 0 | 501 | 0 | 501 |  |  | 1 | 396 |
| TCGA-L5-A8NK-01 | 0 | 412 | 0 | 412 |  |  | 1 | 325 |
| TCGA-L5-A8NL-01 | 0 | 402 | 0 | 402 |  |  | 0 | 402 |
| TCGA-L5-A8NM-01 | 1 | 236 | 0 | 236 |  |  | 0 | 236 |
| TCGA-L5-A8NN-01 | 0 | 167 | 0 | 167 |  |  | 0 | 167 |
| TCGA-L5-A8NQ-01 | 1 | 650 | 1 | 650 |  |  | 1 | 337 |
| TCGA-L5-A8NR-01 | 0 | 265 | 0 | 265 |  |  | 1 | 265 |
| TCGA-L5-A8NS-01 | 0 | 408 | 0 | 408 |  |  | 1 | 300 |
| TCGA-L5-A8NT-01 | 0 | 825 | 0 | 825 |  |  | 1 | 825 |
| TCGA-L5-A8NU-01 | 1 | 2532 | 0 | 2532 |  |  | 0 | 2532 |
| TCGA-L5-A8NV-01 | 1 | 1599 | 1 | 1599 |  |  | 1 | 1252 |
| TCGA-L5-A8NW-01 | 1 | 1402 | 1 | 1402 |  |  | 1 | 1147 |
| TCGA-L7-A56G-01 | 1 | 330 | 1 | 330 |  |  | 1 | 270 |
| TCGA-L7-A6VZ-01 | 0 | 315 | 0 | 315 | 0 | 315 | 0 | 315 |
| TCGA-LN-A49K-01 | 1 | 180 | 0 | 180 | 0 | 180 | 0 | 180 |
| TCGA-LN-A49L-01 | 1 | 318 | 0 | 318 | 0 | 318 | 0 | 318 |
| TCGA-LN-A49M-01 | 0 | 385 | 0 | 385 | 0 | 385 | 0 | 385 |
| TCGA-LN-A49N-01 | 0 | 378 | 0 | 378 | 0 | 378 | 0 | 378 |
| TCGA-LN-A49O-01 | 0 | 408 | 0 | 408 | 1 | 193 | 1 | 193 |
| TCGA-LN-A49P-01 | 0 | 375 | 0 | 375 | 1 | 320 | 1 | 320 |
| TCGA-LN-A49R-01 | 0 | 407 | 0 | 407 | 0 | 407 | 0 | 407 |
| TCGA-LN-A49S-01 | 0 | 400 | 0 | 400 | 1 | 49 | 1 | 49 |
| TCGA-LN-A49U-01 | 0 | 467 | 0 | 467 | 0 | 467 | 0 | 467 |
| TCGA-LN-A49V-01 | 0 | 383 | 0 | 383 | 0 | 383 | 0 | 383 |
| TCGA-LN-A49W-01 | 0 | 403 | 0 | 403 | 0 | 403 | 0 | 403 |
| TCGA-LN-A49X-01 | 0 | 384 | 0 | 384 | 0 | 384 | 0 | 384 |
| TCGA-LN-A49Y-01 | 0 | 379 | 0 | 379 | 1 | 90 | 1 | 90 |
| TCGA-LN-A4A1-01 | 0 | 383 | 0 | 383 | 1 | 205 | 1 | 205 |
| TCGA-LN-A4A2-01 | 0 | 380 | 0 | 380 | 0 | 380 | 0 | 380 |
| TCGA-LN-A4A3-01 | 0 | 388 | 0 | 388 | 0 | 388 | 0 | 388 |
| TCGA-LN-A4A4-01 | 0 | 383 | 0 | 383 | 0 | 383 | 0 | 383 |
| TCGA-LN-A4A5-01 | 1 | 681 | 1 | 681 | 1 | 224 | 1 | 224 |
| TCGA-LN-A4A6-01 | 0 | 391 | 0 | 391 | 1 | 158 | 1 | 158 |
| TCGA-LN-A4A8-01 | 0 | 472 | 0 | 472 | 1 | 250 | 1 | 250 |
| TCGA-LN-A4A9-01 | 1 | 351 | 1 | 351 | 1 | 274 | 1 | 274 |
| TCGA-LN-A4MQ-01 | 0 | 375 | 0 | 375 | 1 | 25 | 1 | 25 |
| TCGA-LN-A4MR-01 | 0 | 402 | 0 | 402 | 1 | 226 | 1 | 226 |
| TCGA-LN-A5U5-01 | 1 | 136 | 1 | 136 |  |  | 1 | 136 |
| TCGA-LN-A5U6-01 | 0 | 375 | 0 | 375 | 0 | 375 | 0 | 375 |
| TCGA-LN-A5U7-01 | 0 | 768 | 0 | 768 | 1 | 535 | 1 | 535 |
| TCGA-LN-A7HV-01 | 0 | 320 | 0 | 320 | 0 | 320 | 0 | 320 |
| TCGA-LN-A7HW-01 | 0 | 365 | 0 | 365 | 1 | 97 | 1 | 97 |
| TCGA-LN-A7HX-01 | 0 | 372 | 0 | 372 | 0 | 372 | 0 | 372 |
| TCGA-LN-A7HY-01 | 0 | 366 | 0 | 366 | 1 | 88 | 1 | 88 |
| TCGA-LN-A7HZ-01 | 0 | 401 | 0 | 401 | 1 | 117 | 1 | 117 |
| TCGA-LN-A8HZ-01 | 0 | 375 | 0 | 375 | 0 | 375 | 0 | 375 |
| TCGA-LN-A8I0-01 | 0 | 407 | 0 | 407 | 1 | 57 | 1 | 57 |
| TCGA-LN-A8I1-01 | 0 | 401 | 0 | 401 | 0 | 401 | 0 | 401 |
| TCGA-LN-A9FO-01 | 0 | 4 | 0 | 4 |  |  | 0 | 4 |
| TCGA-LN-A9FP-01 | 0 | 366 | 0 | 366 | 1 | 290 | 1 | 290 |
| TCGA-LN-A9FQ-01 | 0 | 391 | 0 | 391 | 1 | 82 | 1 | 82 |
| TCGA-LN-A9FR-01 | 0 | 373 | 0 | 373 | 0 | 373 | 0 | 373 |
| TCGA-M9-A5M8-01 | 0 | 1007 | 0 | 1007 | 0 | 1007 | 0 | 1007 |
| TCGA-Q9-A6FU-01 | 1 | 157 | 1 | 157 |  |  | 1 | 99 |
| TCGA-Q9-A6FW-01 | 0 | 238 | 0 | 238 | 0 | 238 | 0 | 238 |
| TCGA-R6-A6DN-01 | 1 | 243 | 1 | 243 |  |  | 1 | 199 |
| TCGA-R6-A6DQ-01 | 1 | 231 | 1 | 231 |  |  | 1 | 231 |
| TCGA-R6-A6KZ-01 | 1 | 154 | 1 | 154 |  |  | 1 | 154 |
| TCGA-R6-A6L4-01 | 1 | 496 | 1 | 496 |  |  | 1 | 160 |
| TCGA-R6-A6L6-01 | 1 | 214 | 1 | 214 |  |  | 1 | 214 |
| TCGA-R6-A6XG-01 | 0 | 1168 | 0 | 1168 | 1 | 572 | 1 | 572 |
| TCGA-R6-A6XQ-01 | 1 | 193 | 1 | 193 |  |  | 1 | 193 |
| TCGA-R6-A6Y0-01 | 0 | 1641 | 0 | 1641 |  |  | 0 | 1641 |
| TCGA-R6-A6Y2-01 | 1 | 283 | 1 | 283 |  |  | 1 | 102 |
| TCGA-R6-A8W5-01 | 1 | 480 | 1 | 480 |  |  | 1 | 336 |
| TCGA-R6-A8W8-01 | 1 | 88 | 1 | 88 |  |  | 1 | 88 |
| TCGA-R6-A8WC-01 | 0 | 70 | 0 | 70 |  |  | 0 | 70 |
| TCGA-R6-A8WG-01 | 1 | 386 | 1 | 386 |  |  | 1 | 107 |
| TCGA-RE-A7BO-01 | 1 | 213 | 0 | 213 |  |  | 0 | 213 |
| TCGA-S8-A6BV-01 | 0 | 609 | 0 | 609 |  |  | 1 | 267 |
| TCGA-S8-A6BW-01 | 0 | 620 | 0 | 620 | 0 | 620 | 0 | 620 |
| TCGA-V5-A7RB-01 | 1 | 161 | 0 | 161 |  |  | 0 | 161 |
| TCGA-V5-A7RC-01 | 1 | 104 | 1 | 104 |  |  | 1 | 88 |
| TCGA-V5-A7RC-06 | 1 | 104 | 1 | 104 |  |  | 1 | 88 |
| TCGA-V5-A7RE-01 | 0 | 500 | 0 | 500 |  |  | 1 | 294 |
| TCGA-V5-A7RE-11 | 0 | 500 | 0 | 500 |  |  | 1 | 294 |
| TCGA-V5-AASV-01 | 0 | 467 | 0 | 467 |  |  | 1 | 322 |
| TCGA-V5-AASW-01 | 0 | 282 | 0 | 282 |  |  | 1 | 282 |
| TCGA-V5-AASX-01 | 0 | 273 | 0 | 273 |  |  | 1 | 273 |
| TCGA-V5-AASX-11 | 0 | 273 | 0 | 273 |  |  | 1 | 273 |
| TCGA-VR-A8EO-01 | 0 | 785 | 0 | 785 | 0 | 785 | 0 | 785 |
| TCGA-VR-A8EP-01 | 0 | 824 | 0 | 824 |  |  | 1 | 242 |
| TCGA-VR-A8EQ-01 | 1 | 694 | 1 | 694 |  |  | 1 | 694 |
| TCGA-VR-A8ER-01 | 1 | 378 | 1 | 378 |  |  | 1 | 378 |
| TCGA-VR-A8ET-01 | 1 | 47 | 0 | 47 |  |  | 0 | 47 |
| TCGA-VR-A8EU-01 | 1 | 557 | 1 | 557 |  |  | 1 | 557 |
| TCGA-VR-A8EW-01 | 1 | 247 | 1 | 247 |  |  | 1 | 247 |
| TCGA-VR-A8EX-01 | 1 | 855 | 1 | 855 |  |  | 1 | 855 |
| TCGA-VR-A8EY-01 | 0 | 1025 | 0 | 1025 | 0 | 1025 | 0 | 1025 |
| TCGA-VR-A8EZ-01 | 1 | 553 | 1 | 553 |  |  | 1 | 379 |
| TCGA-VR-A8Q7-01 | 0 | 1590 | 0 | 1590 | 0 | 1590 | 0 | 1590 |
| TCGA-VR-AA4D-01 | 1 | 1405 | 1 | 1405 |  |  | 1 | 832 |
| TCGA-VR-AA4G-01 | 0 | 549 | 0 | 549 | 0 | 549 | 0 | 549 |
| TCGA-VR-AA7B-01 | 0 | 342 | 0 | 342 |  |  | 1 | 207 |
| TCGA-VR-AA7D-01 | 1 | 279 | 1 | 279 |  |  | 1 | 244 |
| TCGA-VR-AA7I-01 | 1 | 484 | 1 | 484 |  |  | 1 | 484 |
| TCGA-X8-AAAR-01 | 0 | 554 | 0 | 554 | 0 | 554 | 0 | 554 |
| TCGA-XP-A8T6-01 | 1 | 763 | 0 | 763 | 0 | 763 | 0 | 763 |
| TCGA-XP-A8T7-01 | 0 | 1254 | 0 | 1254 | 0 | 1254 | 0 | 1254 |
| TCGA-XP-A8T8-01 | 0 | 437 | 0 | 437 | 0 | 437 | 0 | 437 |
| TCGA-Z6-A8JD-01 | 0 | 104 | 0 | 104 | 0 | 104 | 0 | 104 |
| TCGA-Z6-A8JE-01 | 0 | 64 | 0 | 64 | 0 | 64 | 0 | 64 |
| TCGA-Z6-A9VB-01 | 0 | 40 | 0 | 40 | 0 | 40 | 0 | 40 |
| TCGA-Z6-AAPN-01 | 0 | 81 | 0 | 81 | 0 | 81 | 0 | 81 |
| TCGA-ZR-A9CJ-01 | 1 | 600 | 1 | 600 |  |  | 1 | 163 |

**GSE37200 dataset**

| **Accession_ID** | **Title** | **STAGE** | **DISEASE STATE** |
| --- | --- | --- | --- |
| GSM913527 | BE_01B | NA | Barrett’s esophageal tissues |
| GSM913528 | EAC_02T | III | Esophageal adenocarcinoma |
| GSM913529 | BE_03B | NA | Barrett’s esophageal tissues |
| GSM913530 | EAC_04T | III | Esophageal adenocarcinoma |
| GSM913531 | BE_05B | NA | Barrett’s esophageal tissues |
| GSM913532 | EAC_06T | III | Esophageal adenocarcinoma |
| GSM913533 | BE_07B | NA | Barrett’s esophageal tissues |
| GSM913534 | BE_08B | NA | Barrett’s esophageal tissues |
| GSM913535 | BE_09B | NA | Barrett’s esophageal tissues |
| GSM913536 | BE_10B | NA | Barrett’s esophageal tissues |
| GSM913537 | EAC_11T | I | Esophageal adenocarcinoma |
| GSM913538 | BE_12B | NA | Barrett’s esophageal tissues |
| GSM913539 | EAC_13T | I | Esophageal adenocarcinoma |
| GSM913540 | BE_14B | NA | Barrett’s esophageal tissues |
| GSM913541 | BE_15B | NA | Barrett’s esophageal tissues |
| GSM913542 | BE_16B | NA | Barrett’s esophageal tissues |
| GSM913543 | EAC_17T | III | Esophageal adenocarcinoma |
| GSM913544 | BE_18B | NA | Barrett’s esophageal tissues |
| GSM913545 | EAC_19T | I | Esophageal adenocarcinoma |
| GSM913546 | EAC_20T | IV | Esophageal adenocarcinoma |
| GSM913547 | BE_21B | NA | Barrett’s esophageal tissues |
| GSM913548 | BE_22B | NA | Barrett’s esophageal tissues |
| GSM913549 | BE_23B | NA | Barrett’s esophageal tissues |
| GSM913550 | BE_24B | NA | Barrett’s esophageal tissues |
| GSM913551 | BE_25B | NA | Barrett’s esophageal tissues |
| GSM913552 | BE_26B | NA | Barrett’s esophageal tissues |
| GSM913553 | EAC_27T | IIA | Esophageal adenocarcinoma |
| GSM913554 | BE_28B | NA | Barrett’s esophageal tissues |
| GSM913555 | EAC_29T | III | Esophageal adenocarcinoma |
| GSM913556 | EAC_30T | I | Esophageal adenocarcinoma |
| GSM913557 | BE_31B | NA | Barrett’s esophageal tissues |
| GSM913558 | BE_32B | NA | Barrett’s esophageal tissues |
| GSM913559 | EAC_33T | III | Esophageal adenocarcinoma |
| GSM913560 | BE_34B | NA | Barrett’s esophageal tissues |
| GSM913561 | BE_35B | NA | Barrett’s esophageal tissues |
| GSM913562 | BE_36B | NA | Barrett’s esophageal tissues |
| GSM913563 | EAC_37T | IIA | Esophageal adenocarcinoma |
| GSM913564 | BE_38B | NA | Barrett’s esophageal tissues |
| GSM913565 | BE_39B | NA | Barrett’s esophageal tissues |
| GSM913566 | BE_40B | NA | Barrett’s esophageal tissues |
| GSM913567 | EAC_41T | I | Esophageal adenocarcinoma |
| GSM913568 | BE_42B | NA | Barrett’s esophageal tissues |
| GSM913569 | BE_43B | NA | Barrett’s esophageal tissues |
| GSM913570 | EAC_44T | IIA | Esophageal adenocarcinoma |
| GSM913571 | BE_45B | NA | Barrett’s esophageal tissues |
| GSM913572 | BE_46B | NA | Barrett’s esophageal tissues |

**GSE37201 dataset**

| **Accession_ID** | **Title** | **STAGE** | **DISEASE STATE** |
| --- | --- | --- | --- |
| GSM913573 | EAC_47T | IIB | Esophageal adenocarcinoma |
| GSM913574 | EAC_48T | IIB | Esophageal adenocarcinoma |
| GSM913575 | EAC_49T | III | Esophageal adenocarcinoma |
| GSM913576 | EAC_50T | III | Esophageal adenocarcinoma |
| GSM913577 | EAC_51T | I | Esophageal adenocarcinoma |
| GSM913578 | EAC_52T | III | Esophageal adenocarcinoma |
| GSM913579 | EAC_53T | IIB | Esophageal adenocarcinoma |
| GSM913580 | EAC_54T | III | Esophageal adenocarcinoma |
| GSM913581 | EAC_55T | IIA | Esophageal adenocarcinoma |
| GSM913582 | EAC_56T | IIB | Esophageal adenocarcinoma |
| GSM913583 | EAC_57T | I | Esophageal adenocarcinoma |
| GSM913584 | EAC_58T | IIB | Esophageal adenocarcinoma |
| GSM913585 | EAC_59T | III | Esophageal adenocarcinoma |
| GSM913586 | EAC_60T | IIA | Esophageal adenocarcinoma |
| GSM913587 | EAC_61T | III | Esophageal adenocarcinoma |
| GSM913588 | EAC_62T | III | Esophageal adenocarcinoma |
| GSM913589 | EAC_63T | IIB | Esophageal adenocarcinoma |
| GSM913590 | EAC_64T | IIB | Esophageal adenocarcinoma |
| GSM913591 | EAC_65T | III | Esophageal adenocarcinoma |
| GSM913592 | EAC_66T | IV | Esophageal adenocarcinoma |
| GSM913593 | EAC_67T | III | Esophageal adenocarcinoma |
| GSM913594 | EAC_68T | III | Esophageal adenocarcinoma |

**GSE47404 dataset**

| **Accession_ID** | **Title** | **HISTOLOGY TYPE** |
| --- | --- | --- |
| GSM1148929 | Exp_ESCC_101 | poor |
| GSM1148930 | Exp_ESCC_102 | well |
| GSM1148931 | Exp_ESCC_103 | well |
| GSM1148932 | Exp_ESCC_104 | moderate |
| GSM1148933 | Exp_ESCC_105 | moderate |
| GSM1148934 | Exp_ESCC_106 | well |
| GSM1148935 | Exp_ESCC_107 | well |
| GSM1148936 | Exp_ESCC_108 | moderate |
| GSM1148937 | Exp_ESCC_109 | moderate |
| GSM1148938 | Exp_ESCC_110 | well |
| GSM1148939 | Exp_ESCC_111 | well |
| GSM1148940 | Exp_ESCC_112 | moderate |
| GSM1148941 | Exp_ESCC_113 | moderate |
| GSM1148942 | Exp_ESCC_114 | well |
| GSM1148943 | Exp_ESCC_115 | poor |
| GSM1148944 | Exp_ESCC_116 | poor |
| GSM1148945 | Exp_ESCC_117 | well |
| GSM1148946 | Exp_ESCC_118 | well |
| GSM1148947 | Exp_ESCC_119 | moderate |
| GSM1148948 | Exp_ESCC_120 | well |
| GSM1148949 | Exp_ESCC_121 | well |
| GSM1148950 | Exp_ESCC_122 | NA |
| GSM1148951 | Exp_ESCC_123 | poor |
| GSM1148952 | Exp_ESCC_124 | moderate |
| GSM1148953 | Exp_ESCC_125 | well |
| GSM1148954 | Exp_ESCC_126 | well |
| GSM1148955 | Exp_ESCC_127 | moderate |
| GSM1148956 | Exp_ESCC_128 | moderate |
| GSM1148957 | Exp_ESCC_129 | moderate |
| GSM1148958 | Exp_ESCC_130 | poor |
| GSM1148959 | Exp_ESCC_131 | moderate |
| GSM1148960 | Exp_ESCC_132 | moderate |
| GSM1148961 | Exp_ESCC_133 | well |
| GSM1148962 | Exp_ESCC_134 | moderate |
| GSM1148963 | Exp_ESCC_135 | poor |
| GSM1148964 | Exp_ESCC_136 | moderate |
| GSM1148965 | Exp_ESCC_137 | poor |
| GSM1148966 | Exp_ESCC_138 | moderate |
| GSM1148967 | Exp_ESCC_139 | moderate |
| GSM1148968 | Exp_ESCC_140 | well |
| GSM1148969 | Exp_ESCC_141 | well |
| GSM1148970 | Exp_ESCC_142 | well |
| GSM1148971 | Exp_ESCC_143 | moderate |
| GSM1148972 | Exp_ESCC_144 | moderate |
| GSM1148973 | Exp_ESCC_145 | moderate |
| GSM1148974 | Exp_ESCC_146 | poor |
| GSM1148975 | Exp_ESCC_147 | well |
| GSM1148976 | Exp_ESCC_148 | well |
| GSM1148977 | Exp_ESCC_149 | moderate |
| GSM1148978 | Exp_ESCC_150 | well |
| GSM1148979 | Exp_ESCC_151 | NA |
| GSM1148980 | Exp_ESCC_152 | moderate |
| GSM1148981 | Exp_ESCC_153 | poor |
| GSM1148982 | Exp_ESCC_154 | moderate |
| GSM1148983 | Exp_ESCC_155 | poor |
| GSM1148984 | Exp_ESCC_156 | well |
| GSM1148985 | Exp_ESCC_157 | moderate |
| GSM1148986 | Exp_ESCC_158 | well |
| GSM1148987 | Exp_ESCC_159 | well |
| GSM1148988 | Exp_ESCC_160 | moderate |
| GSM1148989 | Exp_ESCC_161 | NA |
| GSM1148990 | Exp_ESCC_162 | moderate |
| GSM1148991 | Exp_ESCC_163 | moderate |
| GSM1148992 | Exp_ESCC_164 | moderate |
| GSM1148993 | Exp_ESCC_165 | moderate |
| GSM1148994 | Exp_ESCC_166 | moderate |
| GSM1148995 | Exp_ESCC_167 | well |
| GSM1148996 | Exp_ESCC_168 | moderate |
| GSM1148997 | Exp_ESCC_169 | moderate |
| GSM1148998 | Exp_ESCC_170 | moderate |
| GSM1148999 | Exp_ESCC_171 | poor |

**GSE19417 dataset**

| **Accession_ID** | **TUMOR DIFFERENTIATION** | **TUMOR HISTOLOGY** |
| --- | --- | --- |
| GSM482796 | Moderate | Oesophageal adenocarcinoma |
| GSM482797 | Moderate | Oesophageal adenocarcinoma |
| GSM482798 | Moderate | Oesophageal adenocarcinoma |
| GSM482799 | Well | Oesophageal adenocarcinoma |
| GSM482800 | Well | Oesophageal adenocarcinoma |
| GSM482801 | Moderate | Oesophageal adenocarcinoma |
| GSM482802 | Moderate | Oesophageal adenocarcinoma |
| GSM482803 | Poor | Oesophageal adenocarcinoma |
| GSM482804 | Moderate | Oesophageal adenocarcinoma |
| GSM482805 | Poor | Oesophageal adenocarcinoma |
| GSM482806 | Poor | Oesophageal adenocarcinoma |
| GSM482807 | Poor | Oesophageal adenocarcinoma |
| GSM482808 | Poor | Oesophageal adenocarcinoma |
| GSM482809 | Moderate | Gastric adenocarcinoma |
| GSM482810 | Poor | Oesophageal adenocarcinoma |
| GSM482811 | Poor | Oesophageal adenocarcinoma |
| GSM482812 | Moderate | Oesophageal adenocarcinoma |
| GSM482813 | Poor | Oesophageal adenocarcinoma |
| GSM482814 | Poor | Oesophageal adenocarcinoma |
| GSM482815 | Moderate | Oesophageal adenocarcinoma |
| GSM482816 | Poor | Oesophageal adenocarcinoma |
| GSM482817 | Poor | Oesophageal adenocarcinoma |
| GSM482818 | Moderate | Squamous cell carcinoma |
| GSM482819 | Poor | Oesophageal adenocarcinoma |
| GSM482820 | Poor | Squamous cell carcinoma |
| GSM482821 | Well | Squamous cell carcinoma |
| GSM482822 | Moderate | Squamous cell carcinoma |
| GSM482823 | Moderate | Squamous cell carcinoma |
| GSM482824 | Moderate | Squamous cell carcinoma |
| GSM482825 | Poor | Squamous cell carcinoma |
| GSM482826 | Poor | Squamous cell carcinoma |
| GSM482827 | Poor | Oesophageal adenocarcinoma |
| GSM482828 | Moderate | Oesophageal adenocarcinoma |
| GSM482829 | Moderate | Oesophageal adenocarcinoma |
| GSM482830 | Well | Gastric adenocarcinoma |
| GSM482831 | Poor | Gastric adenocarcinoma |
| GSM482832 | Poor | Oesophageal adenocarcinoma |
| GSM482833 | Poor | Gastric adenocarcinoma |
| GSM482834 | Moderate | Oesophageal adenocarcinoma |
| GSM482835 | Moderate | Oesophageal adenocarcinoma |
| GSM482836 | Moderate | Oesophageal adenocarcinoma |
| GSM482837 | Moderate | Oesophageal adenocarcinoma |
| GSM482838 | Well | Oesophageal adenocarcinoma |
| GSM482839 | Moderate | Gastric adenocarcinoma |
| GSM482840 | Moderate | Gastric adenocarcinoma |
| GSM482841 | Poor | Oesophageal adenocarcinoma |
| GSM482842 | Moderate | Oesophageal adenocarcinoma |
| GSM482843 | Poor | Oesophageal adenocarcinoma |
| GSM482844 | Poor | Oesophageal adenocarcinoma |
| GSM482845 | Well | Oesophageal adenocarcinoma |
| GSM482846 | Moderate | Oesophageal adenocarcinoma |
| GSM482847 | Moderate | Oesophageal adenocarcinoma |
| GSM482848 | Poor | Oesophageal adenocarcinoma |
| GSM482849 | Moderate | Gastric adenocarcinoma |
| GSM482850 | Moderate | Gastric adenocarcinoma |
| GSM482851 | Poor | Gastric adenocarcinoma |
| GSM482852 | Poor | Oesophageal adenocarcinoma |
| GSM482853 | Poor | Oesophageal adenocarcinoma |
| GSM482854 | Poor | Oesophageal adenocarcinoma |
| GSM482855 | Poor | Oesophageal adenocarcinoma |
| GSM482856 | Moderate | Gastric adenocarcinoma |
| GSM482857 | Moderate | Oesophageal adenocarcinoma |
| GSM482858 | Well | Oesophageal adenocarcinoma |
| GSM482859 | Poor | Gastric adenocarcinoma |
| GSM482860 | Poor | Gastric adenocarcinoma |
| GSM482861 | Moderate | Oesophageal adenocarcinoma |
| GSM482862 | Poor | Gastric adenocarcinoma |
| GSM482863 | Moderate | Oesophageal adenocarcinoma |
| GSM482864 | Poor | Oesophageal adenocarcinoma |
| GSM482865 | Poor | Oesophageal adenocarcinoma |
| GSM482866 | Moderate | Gastric adenocarcinoma |
| GSM482867 | Poor | Gastric adenocarcinoma |
| GSM482868 | Poor | Oesophageal adenocarcinoma |
| GSM482869 | Poor | Gastric adenocarcinoma |
| GSM482870 | Poor | Oesophageal adenocarcinoma |
| GSM482871 | Poor | Oesophageal adenocarcinoma |

**Liu2021 dataset**

| **Patient NO.** | **Tumour grade** | **N stage** | **Death at follow-up** | **Overall survival (days)** | **Disease free status** | **Disease free survival (days)** |
| --- | --- | --- | --- | --- | --- | --- |
| 1 | Well differentiated | N1 | No | 1853 | No | 1853 |
| 2 | Moderately differentiated | N0 | No | 1974 | No | 1974 |
| 3 | Moderately differentiated | N0 | Yes | 438 | Yes | 405 |
| 4 | Poorly differentiated | N0 | Yes | 648 | Yes | 121 |
| 5 | NA | N0 | No | 1904 | No | 1904 |
| 6 | Moderately differentiated | N0 | Yes | 276 | Yes | 276 |
| 7 | Moderately differentiated | N0 | No | 1892 | No | 1892 |
| 8 | Moderately differentiated | N0 | Yes | 507 | Yes | 232 |
| 9 | NA | N0 | No | 2546 | No | 2546 |
| 10 | Well differentiated | N0 | No | 2634 | No | 2634 |
| 11 | Well differentiated | N0 | No | 2151 | No | 2151 |
| 12 | Moderately differentiated | N0 | No | 2616 | No | 2616 |
| 13 | Moderately differentiated | N0 | No | 2672 | No | 2672 |
| 14 | Moderately differentiated | N0 | No | 1832 | No | 1832 |
| 15 | Well differentiated | N0 | Yes | 350 | Yes | 338 |
| 16 | NA | N0 | Yes | 1397 | Yes | 1397 |
| 17 | Poorly differentiated | N0 | No | 2701 | No | 2701 |
| 18 | NA | N1 | Yes | 260 | Yes | 260 |
| 19 | Moderately differentiated | N0 | Yes | 1841 | Yes | 1841 |
| 20 | Moderately differentiated | N0 | No | 1293 | No | 1293 |
| 21 | Moderately differentiated | N0 | No | 1943 | No | 1943 |
| 22 | Moderately differentiated | N0 | Yes | 1017 | Yes | 436 |
| 23 | Moderately differentiated | N1 | No | 2192 | Yes | 1666 |
| 24 | Well differentiated | N1 | No | 2583 | No | 2583 |
| 25 | Moderately differentiated | N1 | No | 483 | No | 483 |
| 26 | Moderately differentiated | N1 | No | 2237 | No | 2237 |
| 27 | Moderately differentiated | N1 | Yes | 911 | Yes | 383 |
| 28 | Well differentiated | N1 | Yes | 807 | Yes | 331 |
| 29 | Moderately differentiated | N1 | Yes | 616 | Yes | 616 |
| 30 | Moderately differentiated | N1 | Yes | 1060 | Yes | 1060 |
| 31 | Poorly differentiated | N2 | No | 2062 | No | 2062 |
| 32 | Moderately differentiated | N1 | Yes | 349 | Yes | 316 |
| 33 | Moderately differentiated | N1 | No | 2120 | No | 2120 |
| 34 | Moderately differentiated | N1 | Yes | 1338 | Yes | 1296 |
| 35 | Poorly differentiated | N0 | Yes | 1957 | Yes | 1957 |
| 36 | Well differentiated | N0 | Yes | 744 | Yes | 809 |
| 37 | Moderately differentiated | N0 | No | 2669 | No | 2669 |
| 38 | Well differentiated | N0 | No | 2689 | No | 2689 |
| 39 | Well differentiated | N0 | No | 2699 | Yes | 1235 |
| 40 | Poorly differentiated | N0 | Yes | 409 | Yes | 409 |
| 41 | Moderately differentiated | N0 | Yes | 634 | Yes | 443 |
| 42 | Poorly differentiated | N0 | Yes | 1529 | Yes | 1172 |
| 43 | Moderately differentiated | N0 | No | 2035 | No | 2035 |
| 44 | Moderately differentiated | N0 | No | 2055 | No | 2055 |
| 45 | Moderately differentiated | N0 | No | 2146 | No | 2146 |
| 46 | Well differentiated | N0 | Yes | 254 | Yes | 112 |
| 47 | Moderately differentiated | N0 | Yes | 533 | Yes | 533 |
| 48 | Moderately differentiated | N0 | No | 2249 | No | 2249 |
| 49 | Well differentiated | N0 | No | 2256 | No | 2256 |
| 50 | Moderately differentiated | N0 | Yes | 535 | Yes | 535 |
| 51 | Moderately differentiated | N0 | No | 2249 | No | 2249 |
| 52 | Moderately differentiated | N0 | No | 1891 | No | 1891 |
| 53 | Moderately differentiated | N0 | No | 1916 | No | 1916 |
| 54 | Moderately differentiated | N0 | Yes | 1291 | No | 1291 |
| 55 | Moderately differentiated | N0 | Yes | 500 | Yes | 351 |
| 56 | Moderately differentiated | N0 | Yes | 2146 | Yes | 630 |
| 57 | Moderately differentiated | N0 | No | 2096 | No | 2096 |
| 58 | Moderately differentiated | N0 | Yes | 1884 | No | 1884 |
| 59 | Moderately differentiated | N1 | Yes | 245 | Yes | 245 |
| 60 | Poorly differentiated | N0 | Yes | 622 | Yes | 503 |
| 61 | Moderately differentiated | N0 | Yes | 920 | Yes | 662 |
| 62 | Moderately differentiated | N0 | No | 1540 | No | 1540 |
| 63 | Moderately differentiated | N0 | No | 2480 | Yes | 1119 |
| 64 | Poorly differentiated | N0 | Yes | 286 | Yes | 286 |
| 65 | Moderately differentiated | N0 | No | 2597 | No | 2597 |
| 66 | Moderately differentiated | N0 | No | 2613 | No | 2613 |
| 67 | Poorly differentiated | N0 | Yes | 310 | Yes | 225 |
| 68 | Moderately differentiated | N0 | Yes | 364 | Yes | 300 |
| 69 | Moderately differentiated | N0 | No | 2621 | No | 2621 |
| 70 | Moderately differentiated | N1 | No | 1872 | No | 1872 |
| 71 | Moderately differentiated | N1 | Yes | 1099 | Yes | 1099 |
| 72 | Moderately differentiated | N1 | No | 655 | No | 655 |
| 73 | Moderately differentiated | N1 | Yes | 246 | Yes | 246 |
| 74 | Poorly differentiated | N2 | Yes | 588 | Yes | 254 |
| 75 | Moderately differentiated | N1 | No | 2053 | No | 2053 |
| 76 | Poorly differentiated | N1 | No | 2075 | Yes | 1244 |
| 77 | Moderately differentiated | N1 | Yes | 1315 | Yes | 1215 |
| 78 | Poorly differentiated | N1 | Yes | 606 | Yes | 469 |
| 79 | Poorly differentiated | N1 | No | 2237 | No | 2237 |
| 80 | Moderately differentiated | N1 | Yes | 934 | Yes | 363 |
| 81 | Moderately differentiated | N1 | No | 2258 | Yes | 787 |
| 82 | Moderately differentiated | N1 | No | 2370 | No | 2370 |
| 83 | Moderately differentiated | N1 | Yes | 346 | Yes | 168 |
| 84 | Moderately differentiated | N1 | Yes | 338 | Yes | 338 |
| 85 | Moderately differentiated | N1 | No | 116 | No | 116 |
| 86 | Well differentiated | N1 | No | 2578 | No | 2578 |
| 87 | Moderately differentiated | N1 | Yes | 341 | Yes | 341 |
| 88 | Moderately differentiated | N2 | Yes | 460 | Yes | 462 |
| 89 | Moderately differentiated | N2 | Yes | 240 | Yes | 240 |
| 90 | Poorly differentiated | N2 | Yes | 1747 | Yes | 656 |
| 91 | Moderately differentiated | N2 | Yes | 48 | Yes | 48 |
| 92 | Moderately differentiated | N2 | Yes | 494 | Yes | 357 |
| 93 | Moderately differentiated | N2 | Yes | 735 | Yes | 735 |
| 94 | Moderately differentiated | N2 | Yes | 572 | Yes | 464 |
| 95 | Moderately differentiated | N2 | Yes | 157 | Yes | 157 |
| 96 | Well differentiated | N2 | Yes | 189 | Yes | 189 |
| 97 | Moderately differentiated | N2 | Yes | 253 | Yes | 127 |
| 98 | Poorly differentiated | N2 | Yes | 440 | Yes | 29 |
| 99 | Moderately differentiated | N2 | Yes | 260 | Yes | 260 |
| 100 | Well differentiated | N2 | Yes | 373 | Yes | 373 |
| 101 | Moderately differentiated | N2 | Yes | 813 | Yes | 815 |
| 102 | Moderately differentiated | N2 | Yes | 606 | Yes | 572 |
| 103 | Moderately differentiated | N2 | No | 2418 | Yes | 1538 |
| 104 | Poorly differentiated | N2 | Yes | 493 | Yes | 493 |
| 105 | Well differentiated | N2 | Yes | 572 | Yes | 367 |
| 106 | Well differentiated | N0 | No | 2056 | No | 2056 |
| 107 | Well differentiated | N0 | No | 1974 | No | 1974 |
| 108 | Moderately differentiated | N0 | Yes | 579 | Yes | 579 |
| 109 | NA | N0 | Yes | 298 | Yes | 278 |
| 110 | Poorly differentiated | N0 | No | 2642 | Yes | 369 |
| 111 | Moderately differentiated | N0 | Yes | 771 | Yes | 771 |
| 112 | Moderately differentiated | N0 | Yes | 1129 | Yes | 1129 |
| 113 | Poorly differentiated | N1 | Yes | 540 | Yes | 389 |
| 114 | Moderately differentiated | N1 | Yes | 1931 | Yes | 1523 |
| 115 | Moderately differentiated | N1 | No | 2689 | No | 2689 |
| 116 | Moderately differentiated | N2 | Yes | 600 | Yes | 600 |
| 117 | Moderately differentiated | N3 | Yes | 1420 | Yes | 563 |
| 118 | Moderately differentiated | N3 | Yes | 289 | Yes | 289 |
| 119 | Moderately differentiated | N3 | Yes | 774 | Yes | 393 |
| 120 | Moderately differentiated | N3 | Yes | 357 | Yes | 55 |
| 121 | NA | N3 | Yes | 280 | Yes | 280 |
| 122 | Moderately differentiated | N3 | Yes | 213 | Yes | 213 |
| 123 | Poorly differentiated | N3 | Yes | 302 | Yes | 102 |
| 124 | Moderately differentiated | N2 | Yes | 646 | Yes | 510 |
