# Supplementary material for: TRIM29 hypermethylation drives esophageal cancer progression via suppression of ZNF750
Source: Cell Death Discov. 2023 Jun 26;9:191. doi: 10.1038/s41420-023-01491-1 (PMC10293201; doi:10.1038/s41420-023-01491-1)

**Original western blots：**

**Figure 2V**


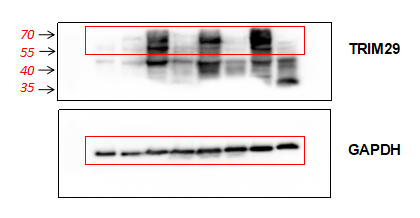


**Figure 4J**


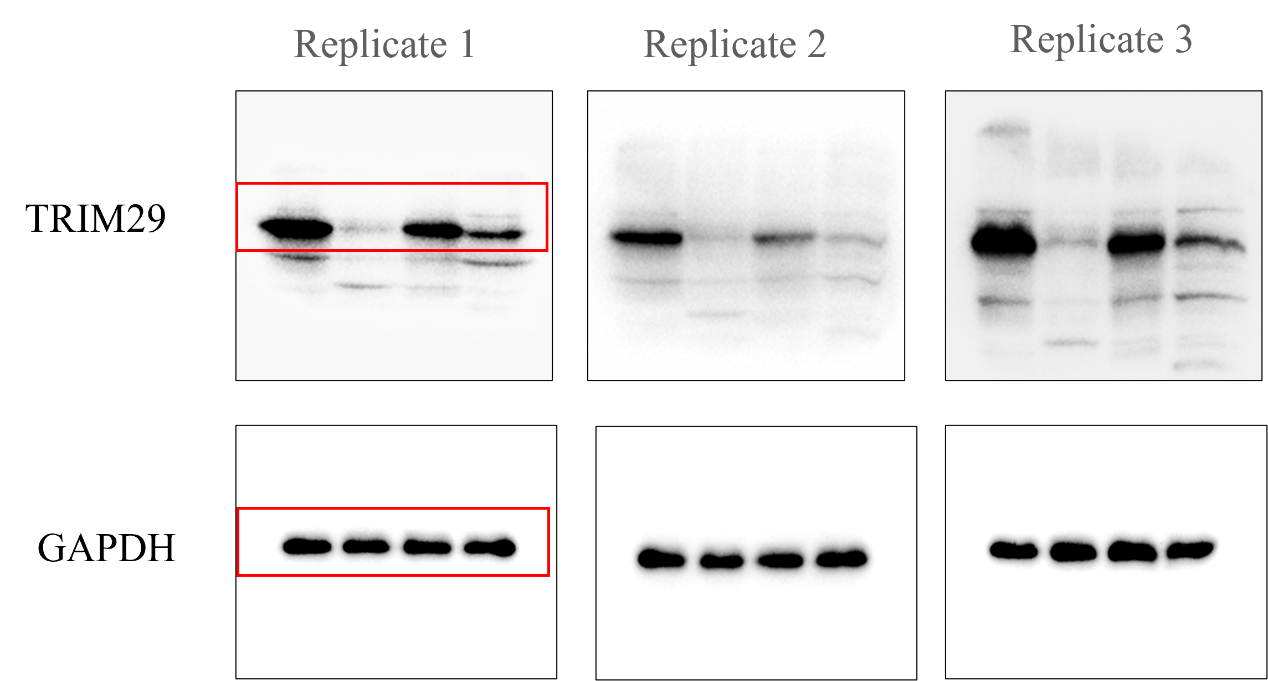


**Figure 4L**


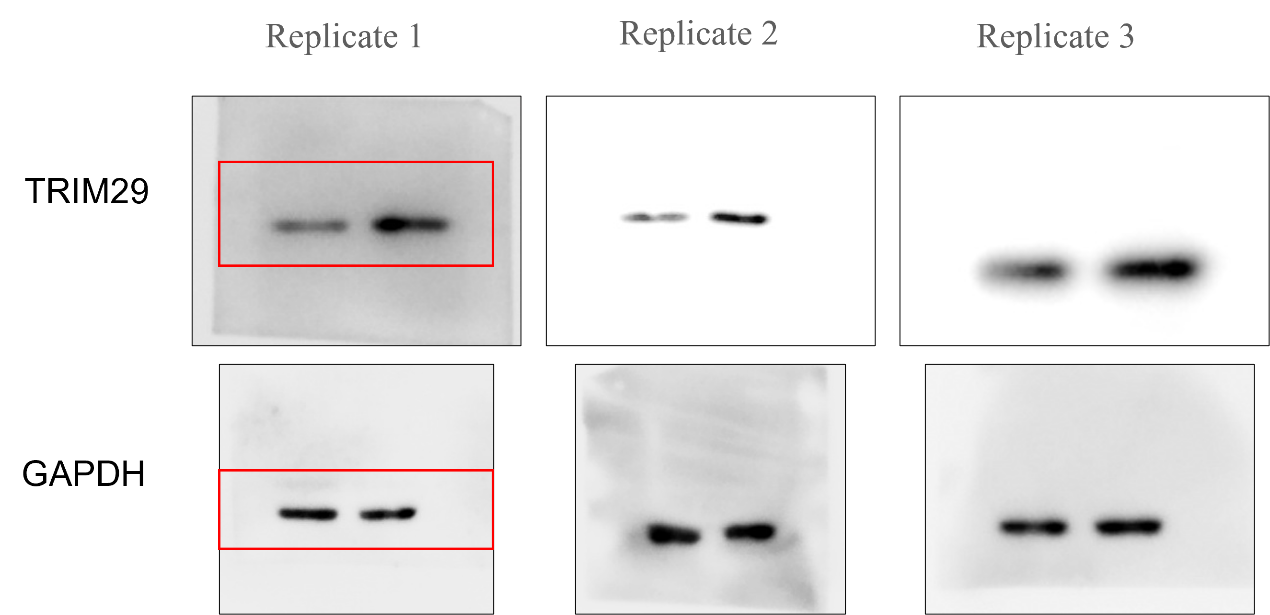


**Figure 5H**

**KYSE30**


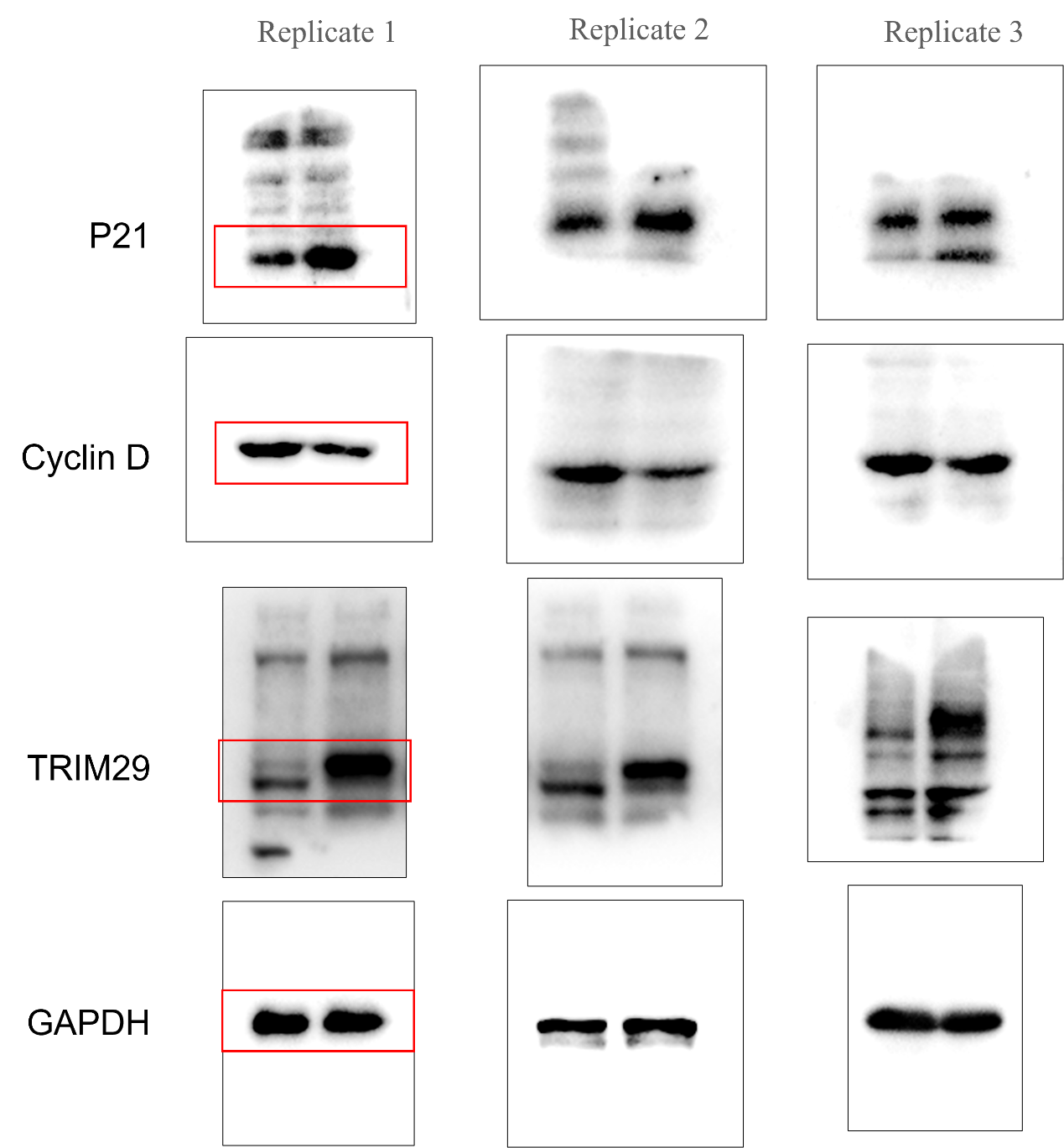


**Figure 5H**

**KYSE410**


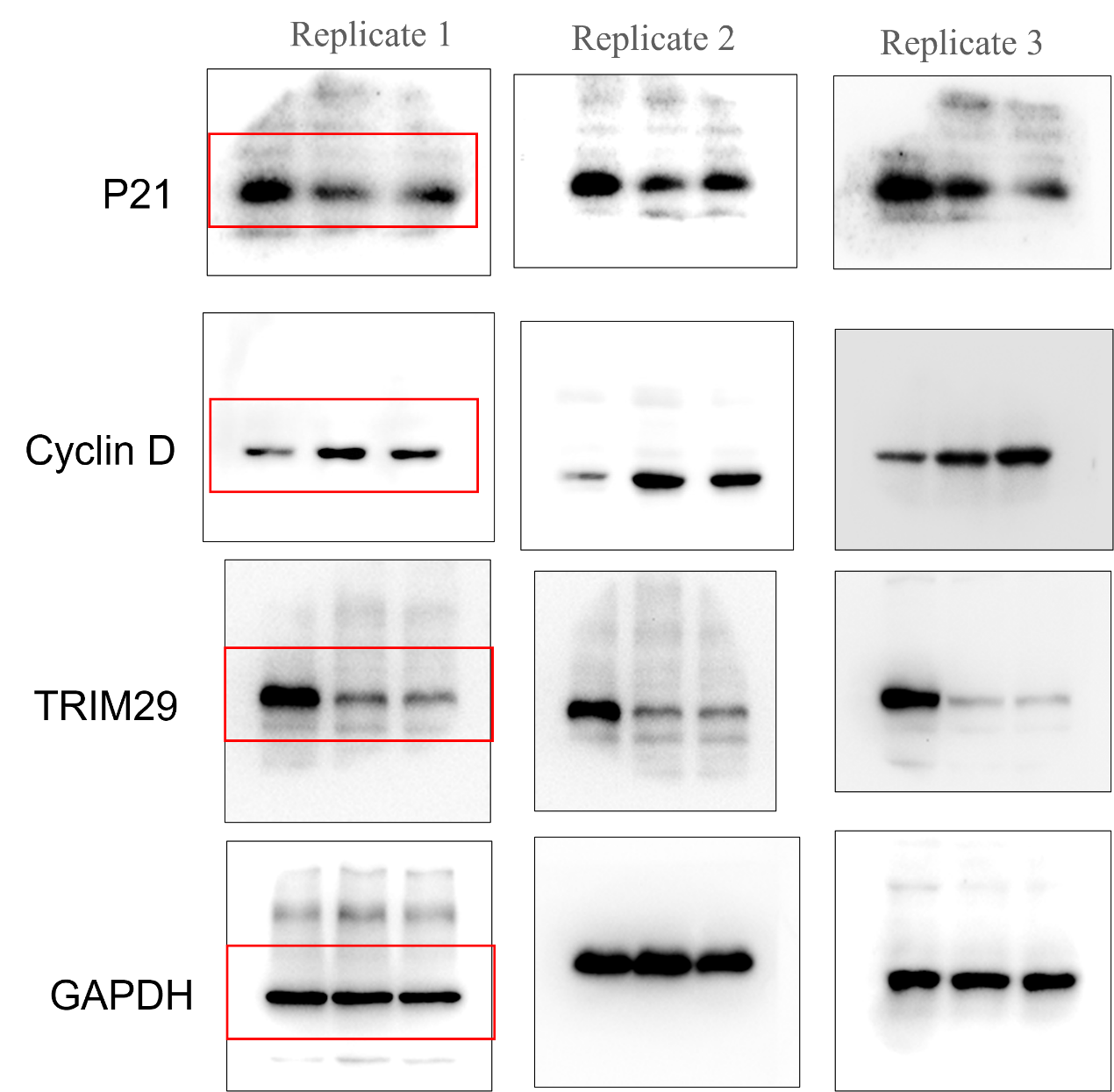


**Figure 5H**

**KYSE450**


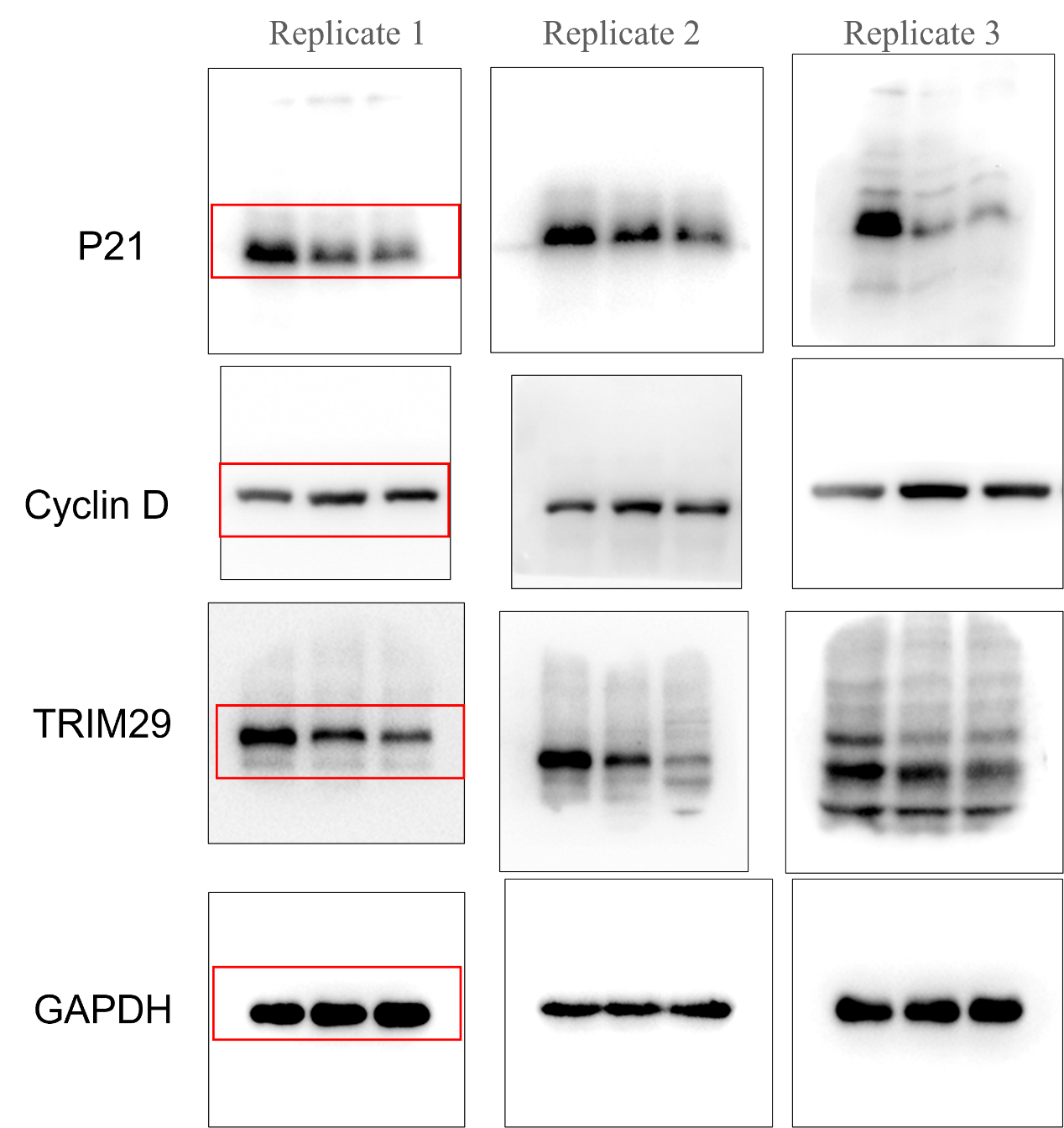


**Figure 6C**

**K30**


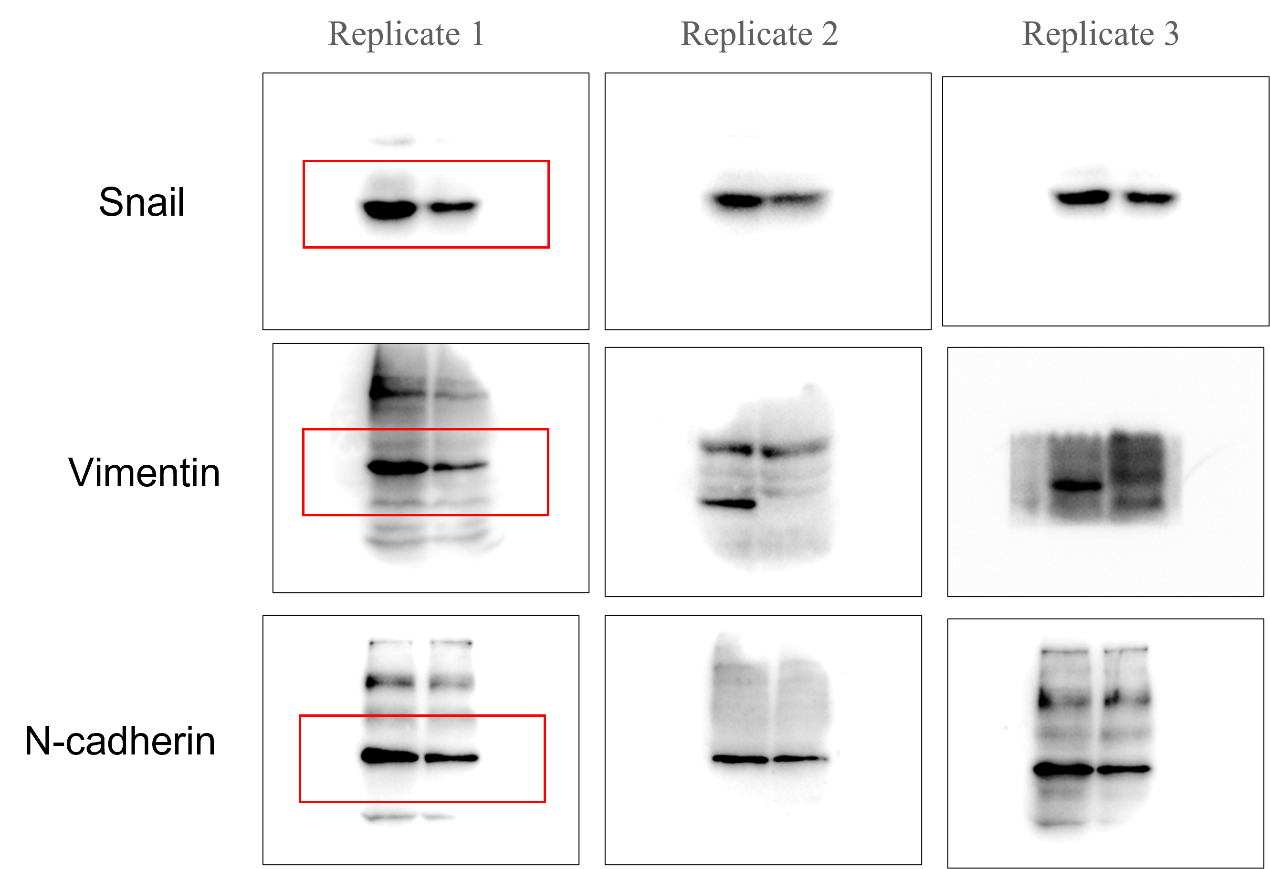


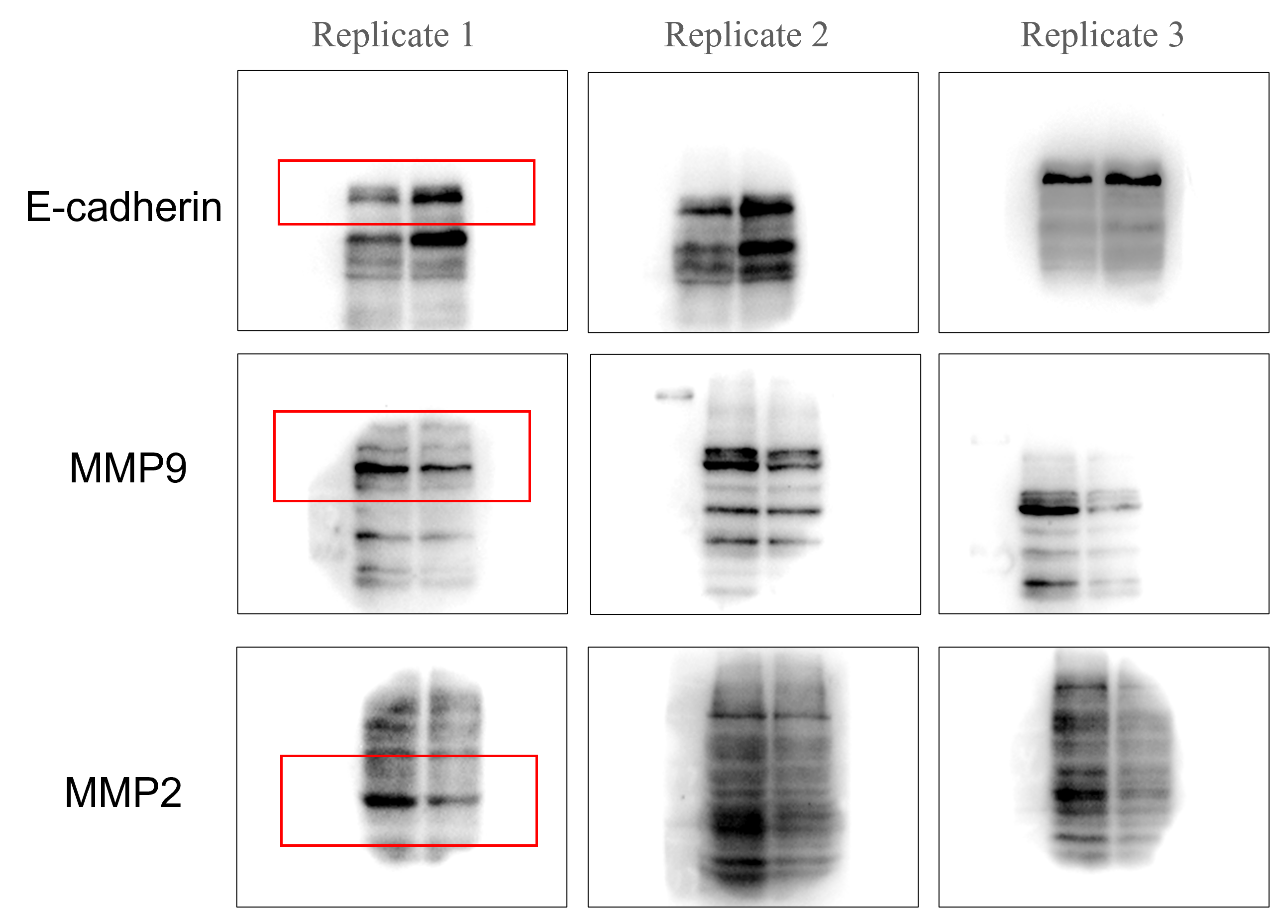


**Figure 6C**

**K410**


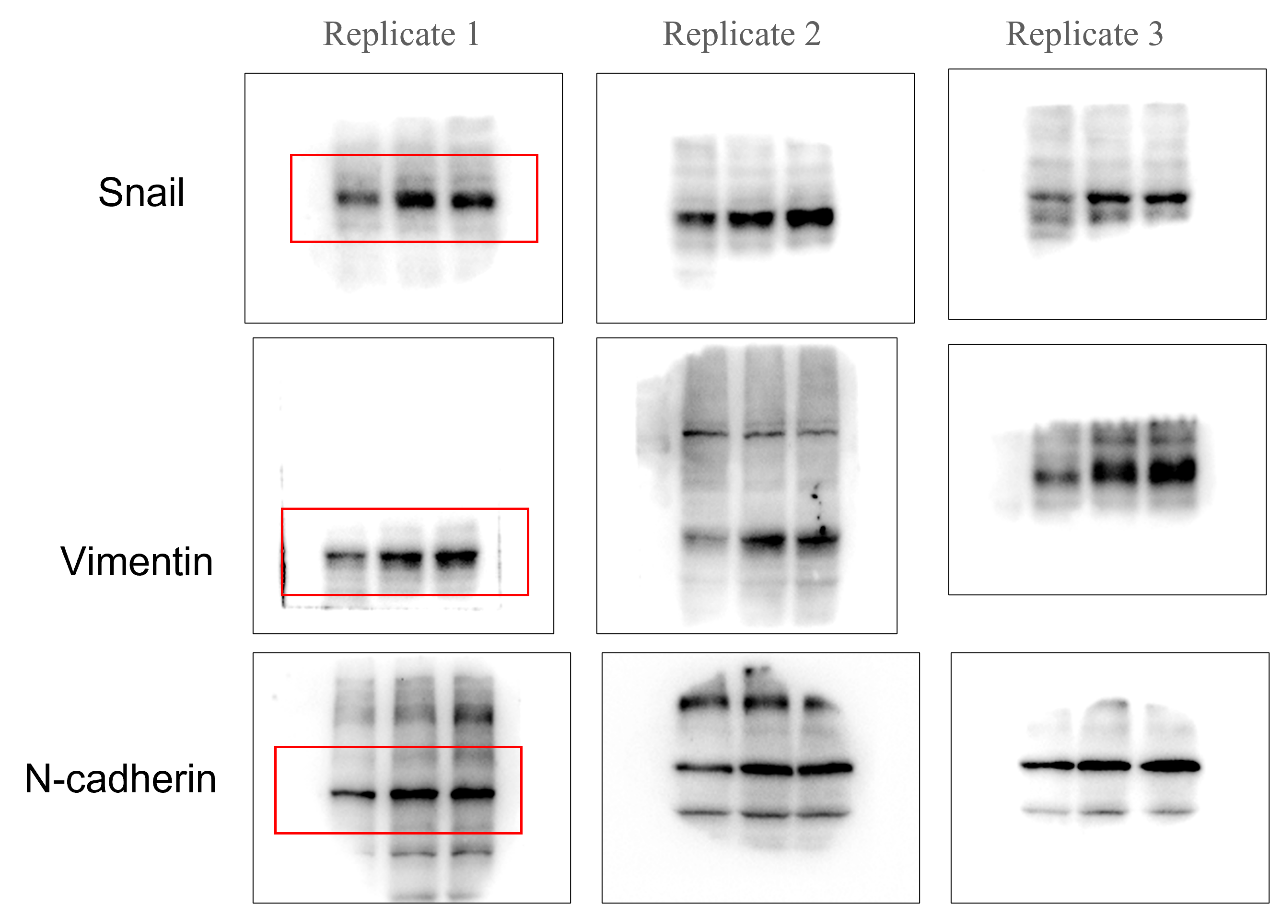


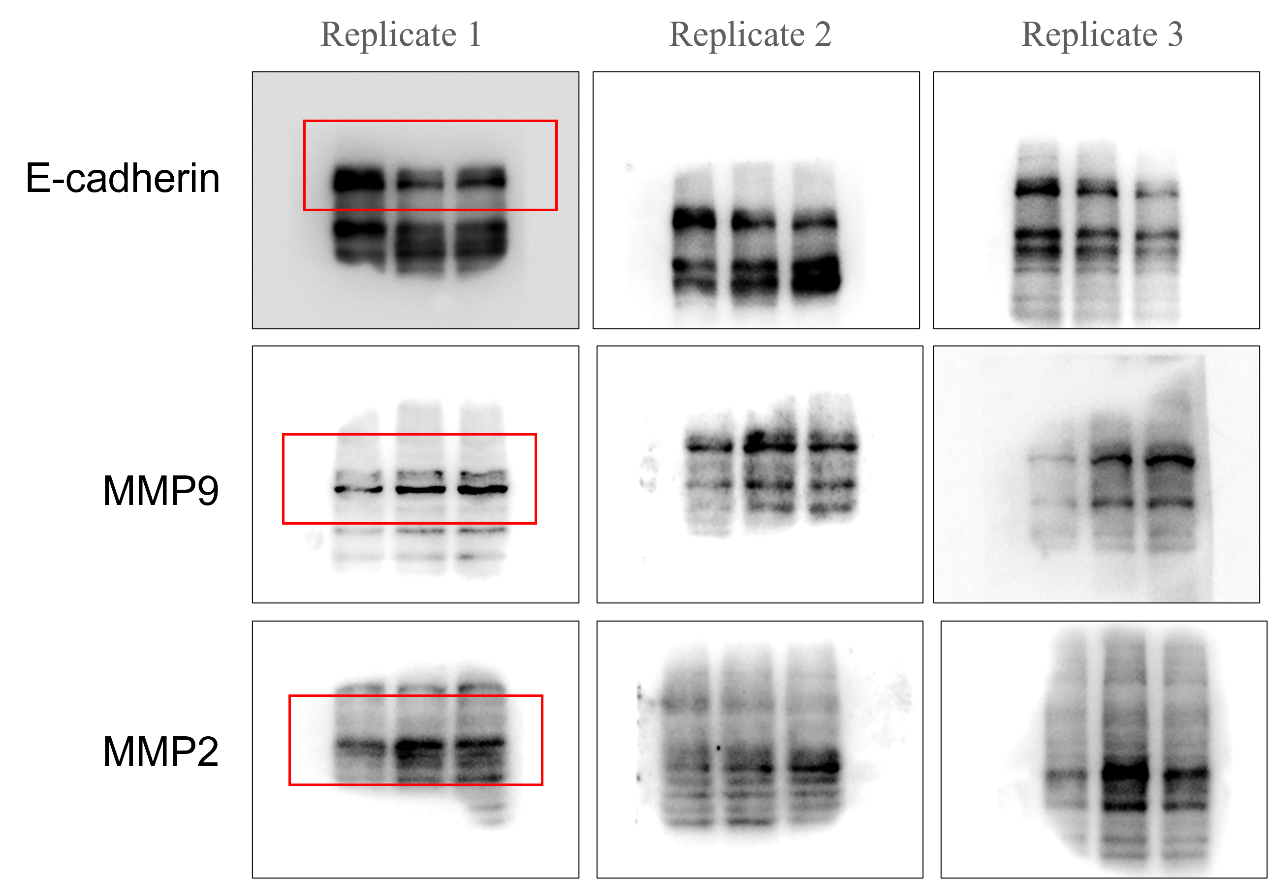


**Figure 6C**

**K450**


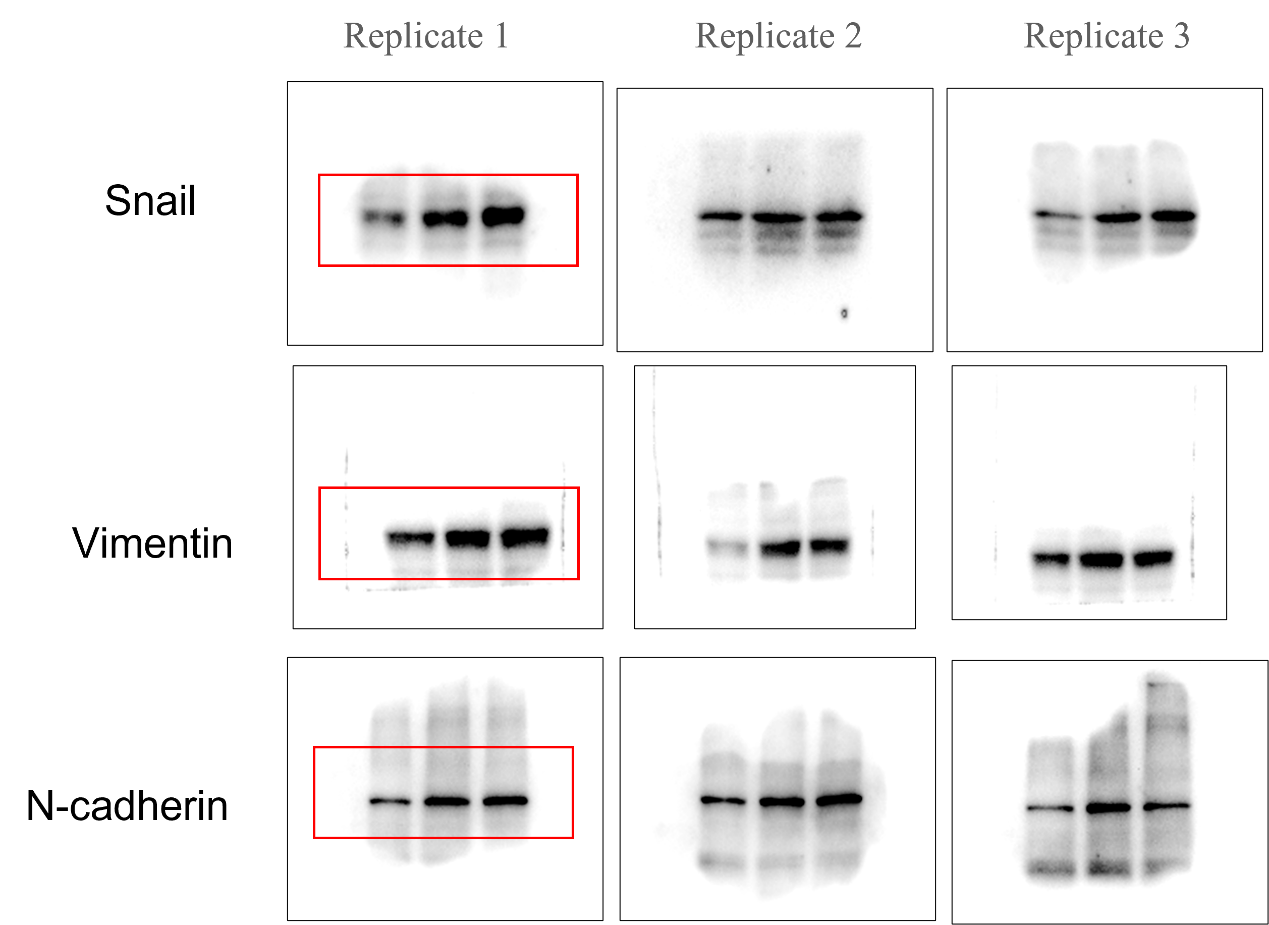


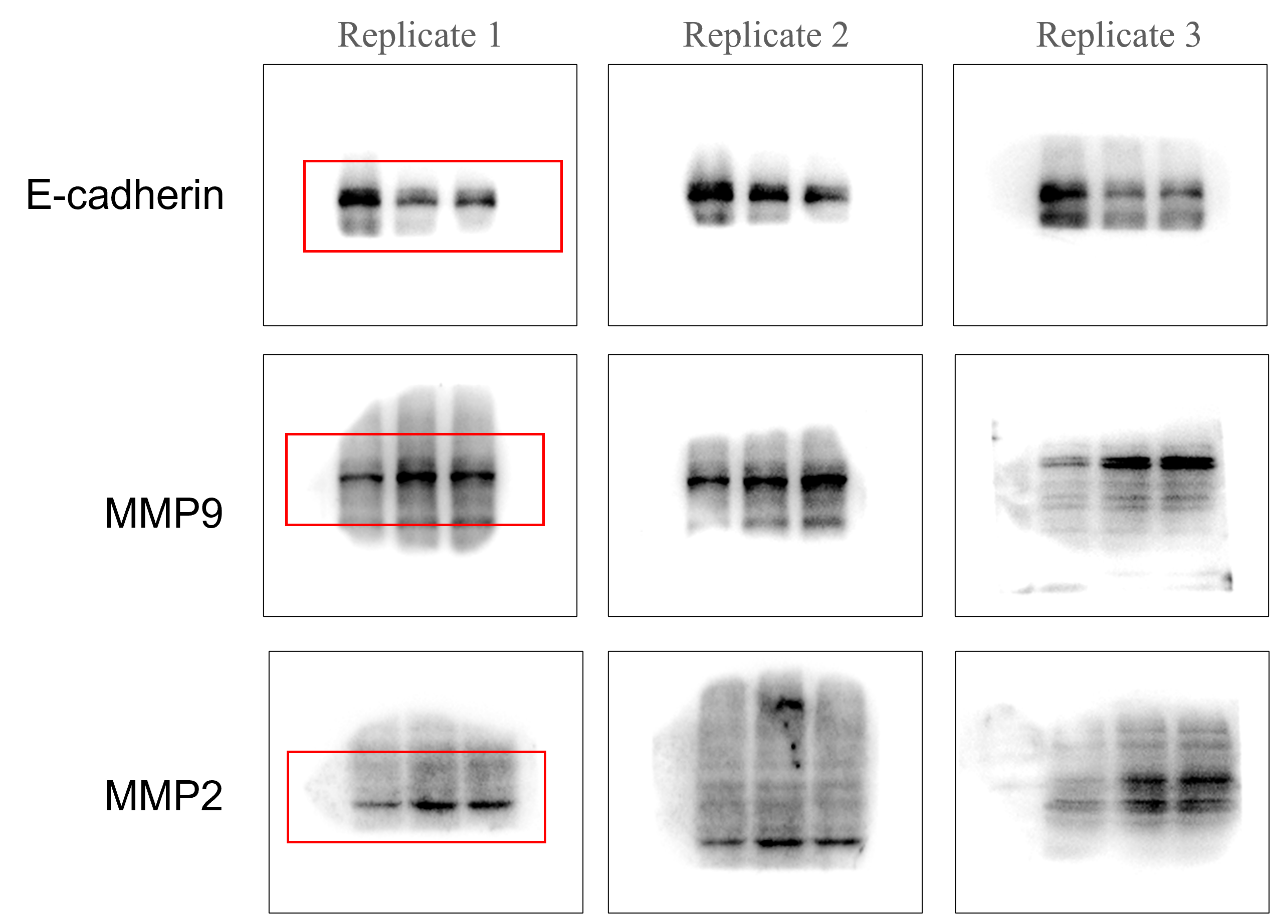


**Figure 7A**

**K30**


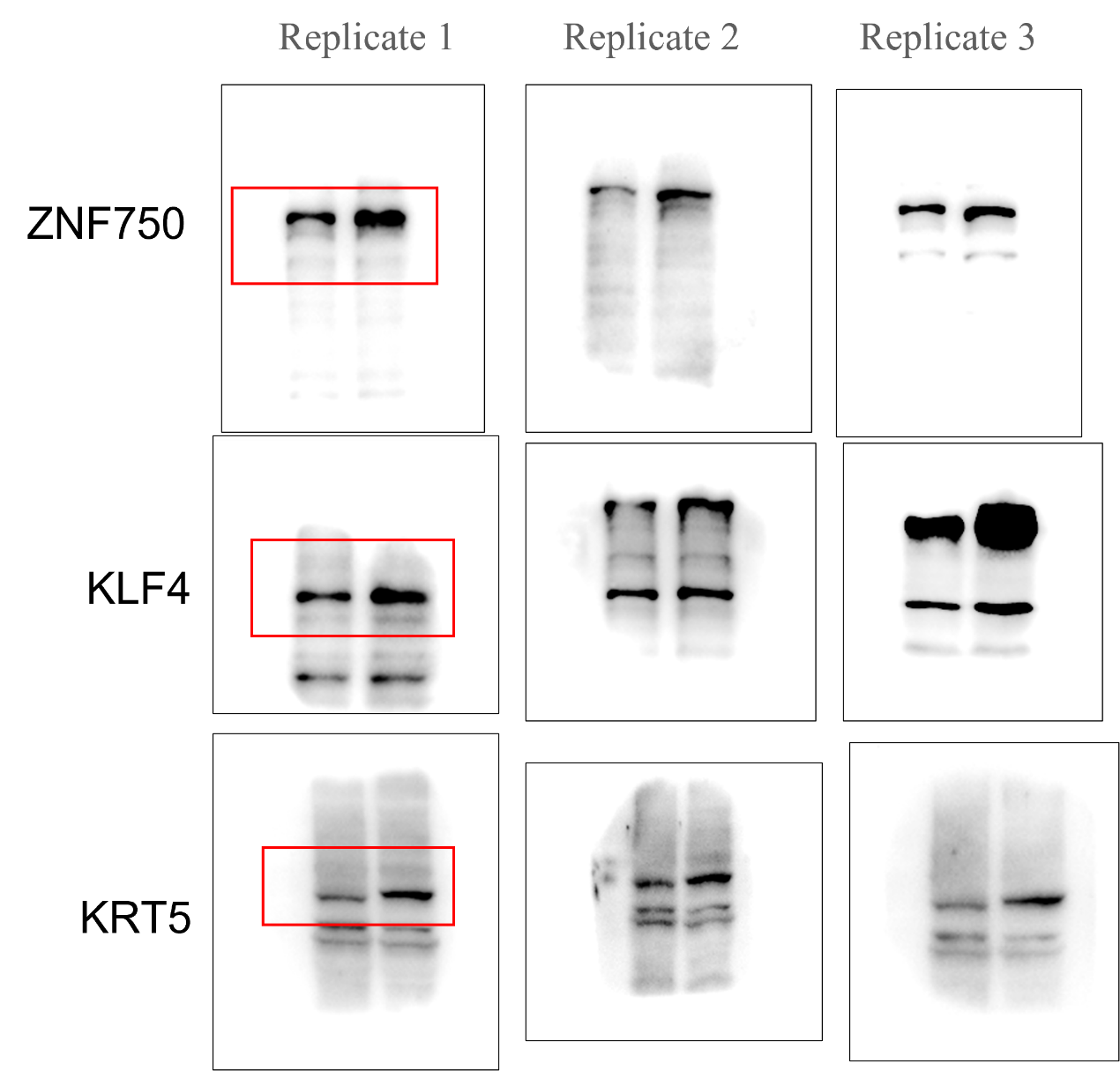


**Figure 7A**

**K410**


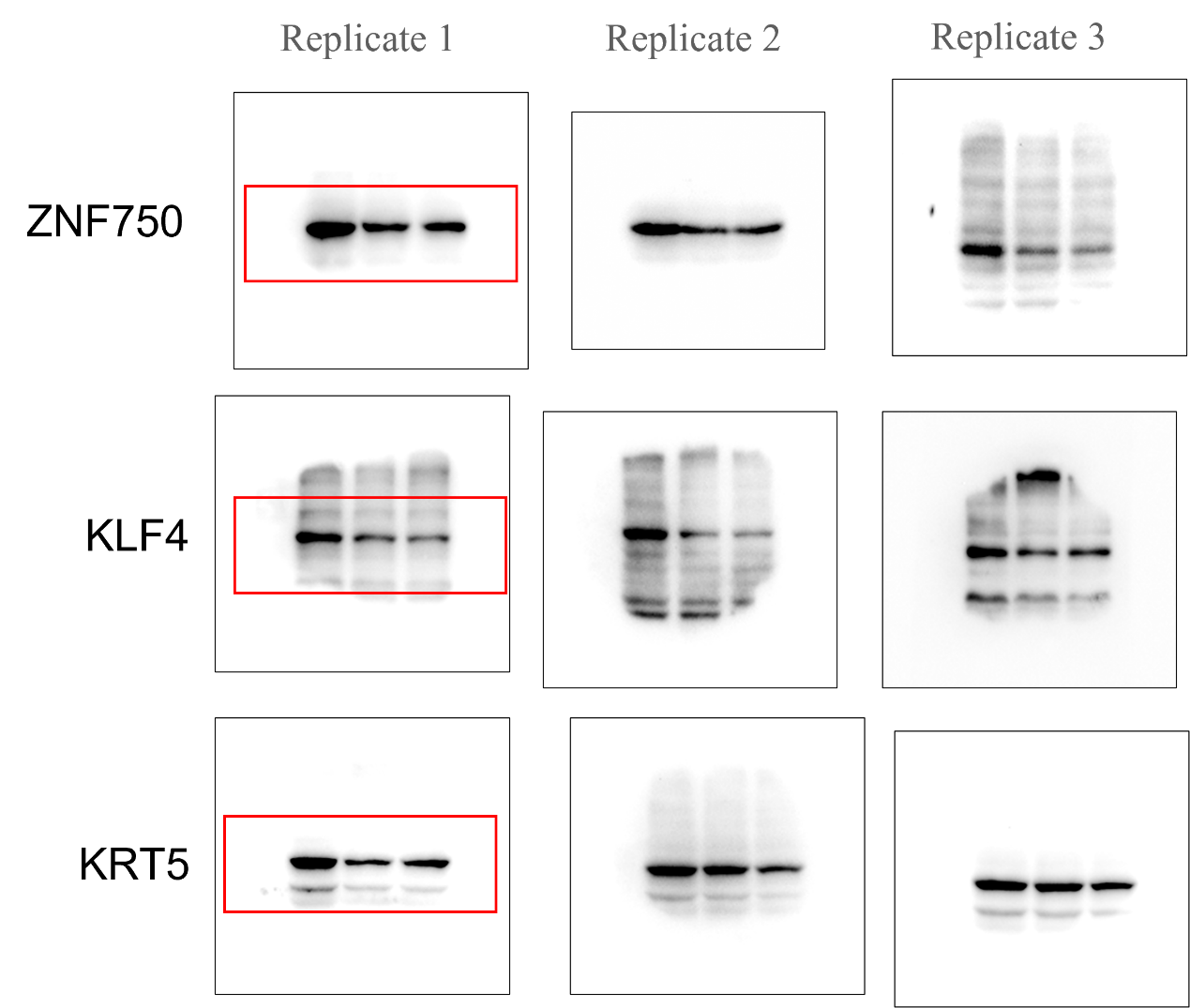


**Figure 7A**

**K450**


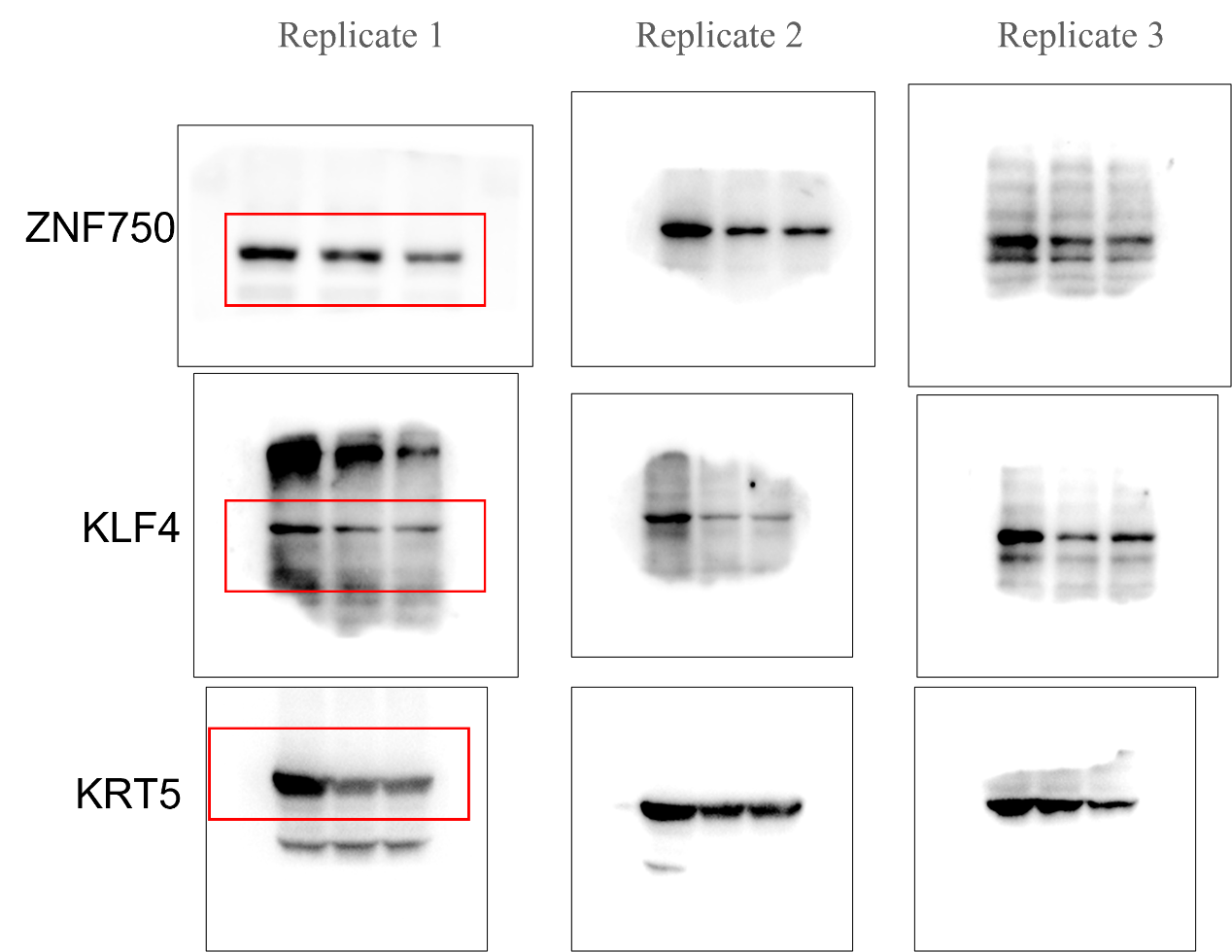


**Figure 7E**


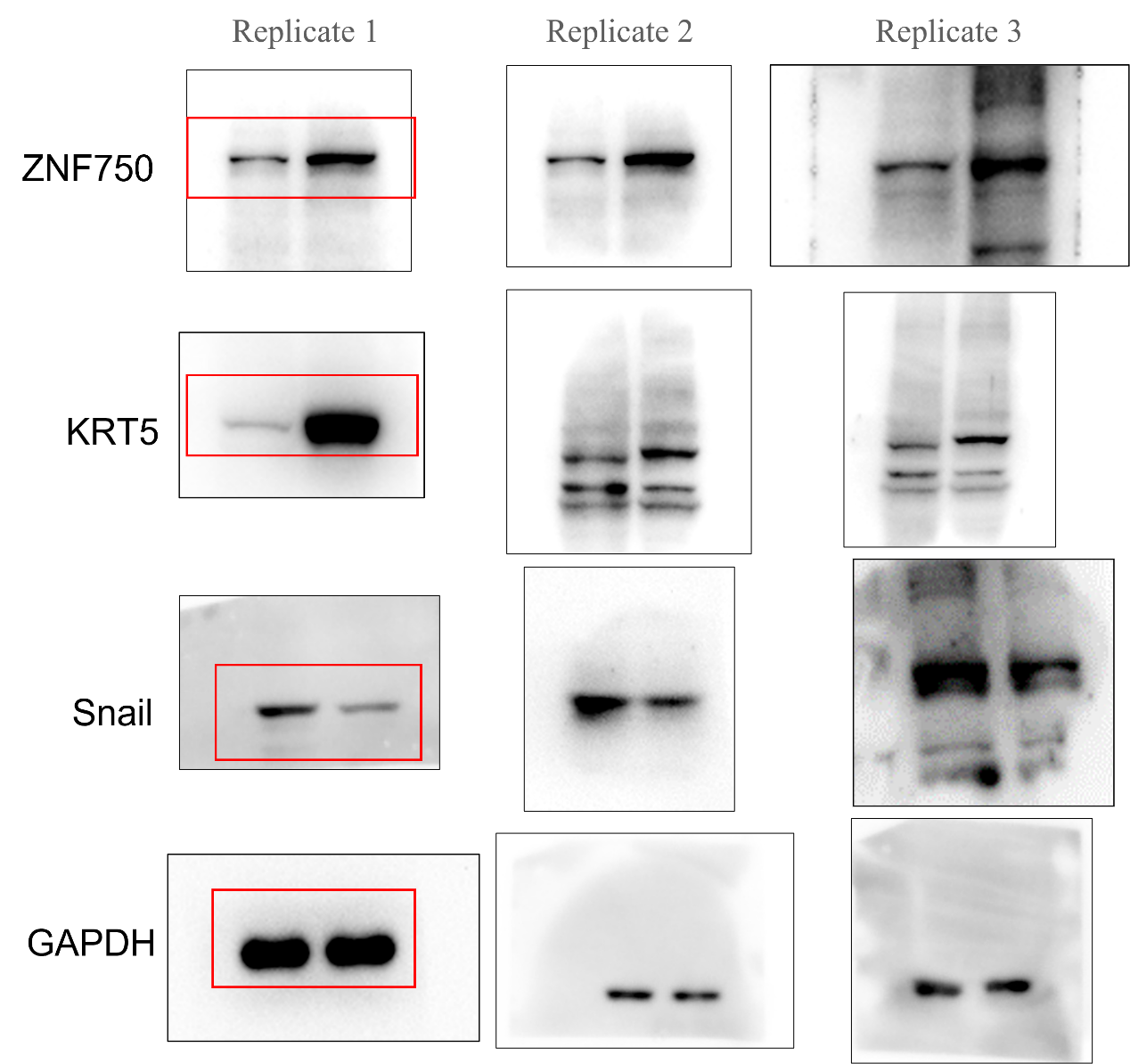


**Figure 8E**

**K30**


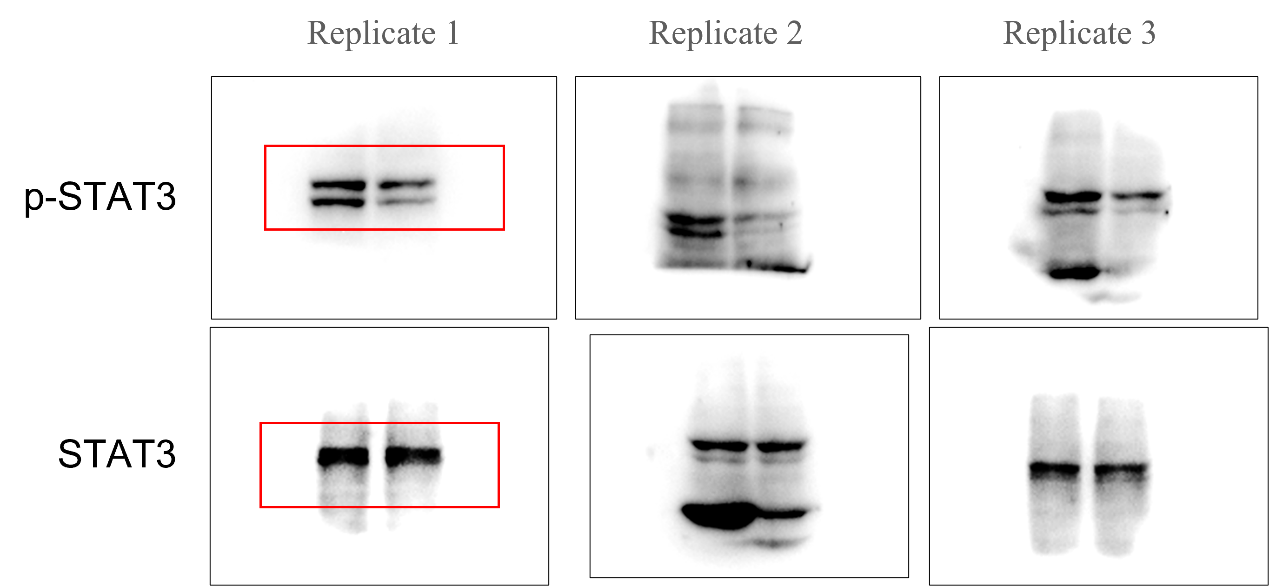


**Figure 8E**

**K410**


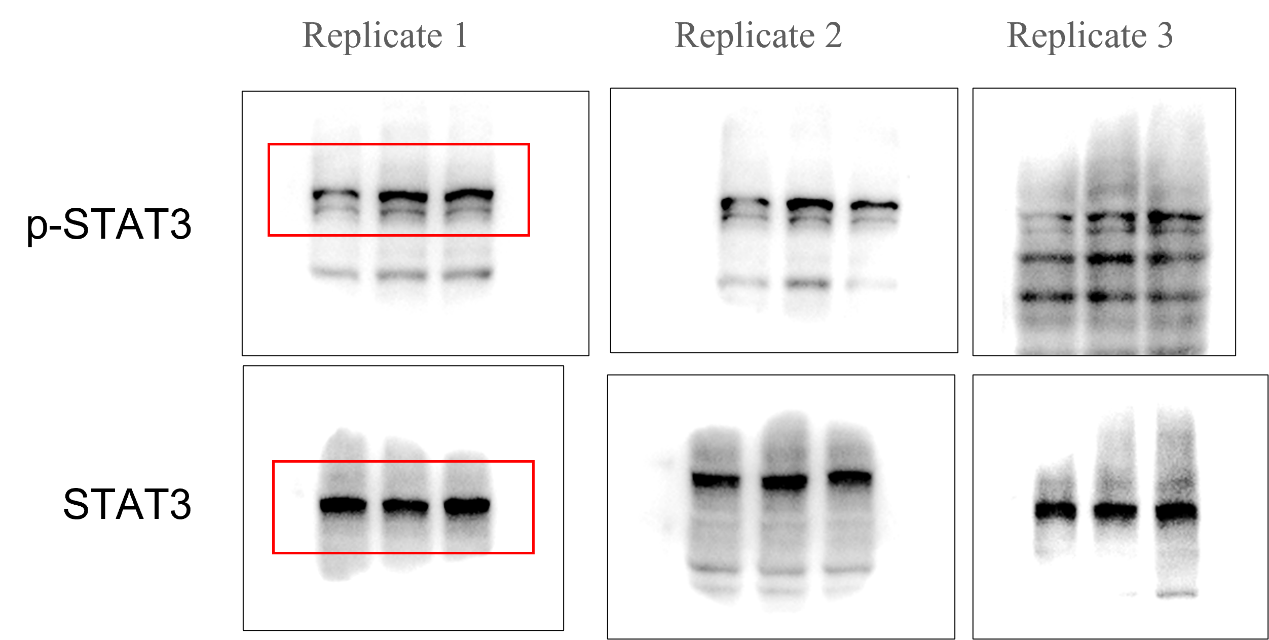


**Figure 8E**

**K450**


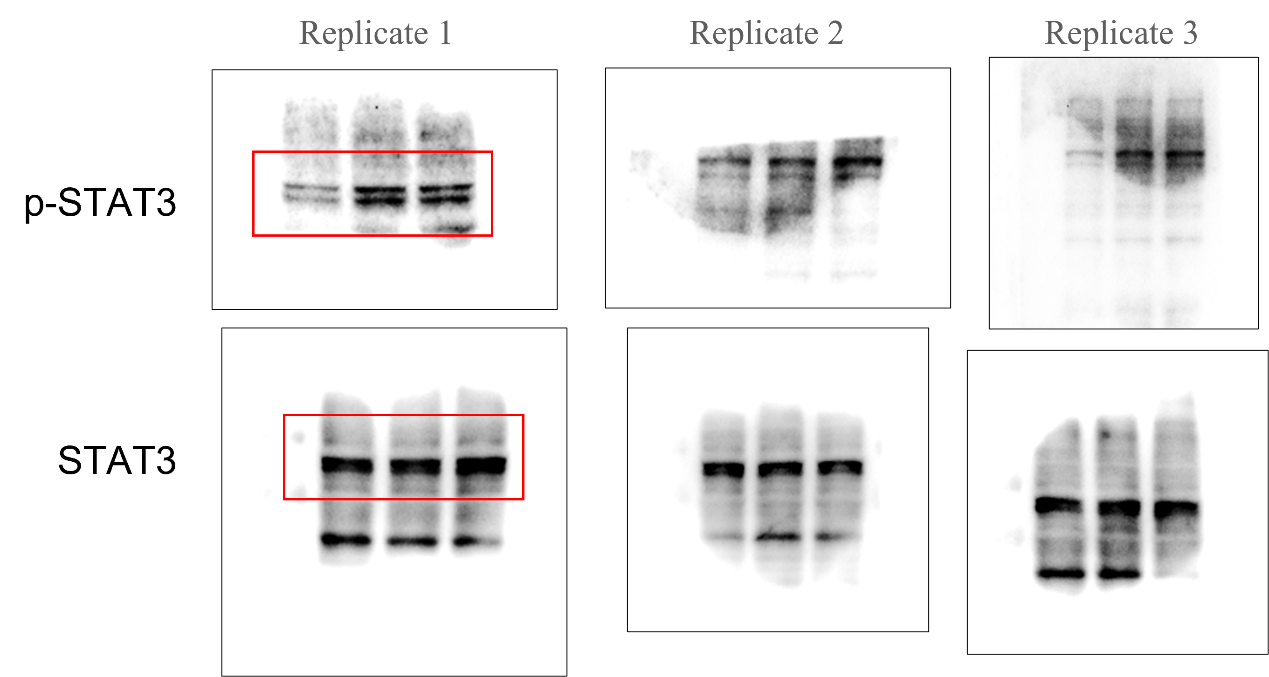


**Figure 8F**

**K410**


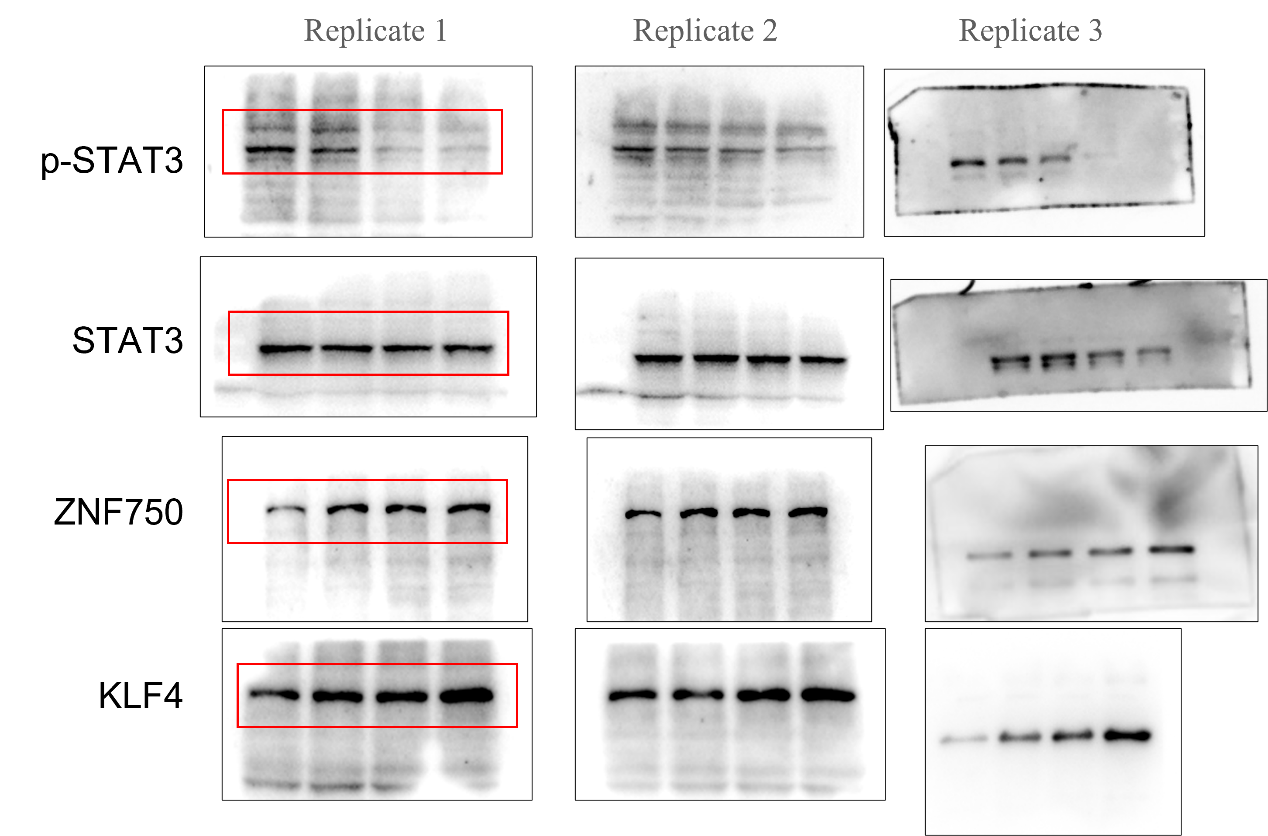


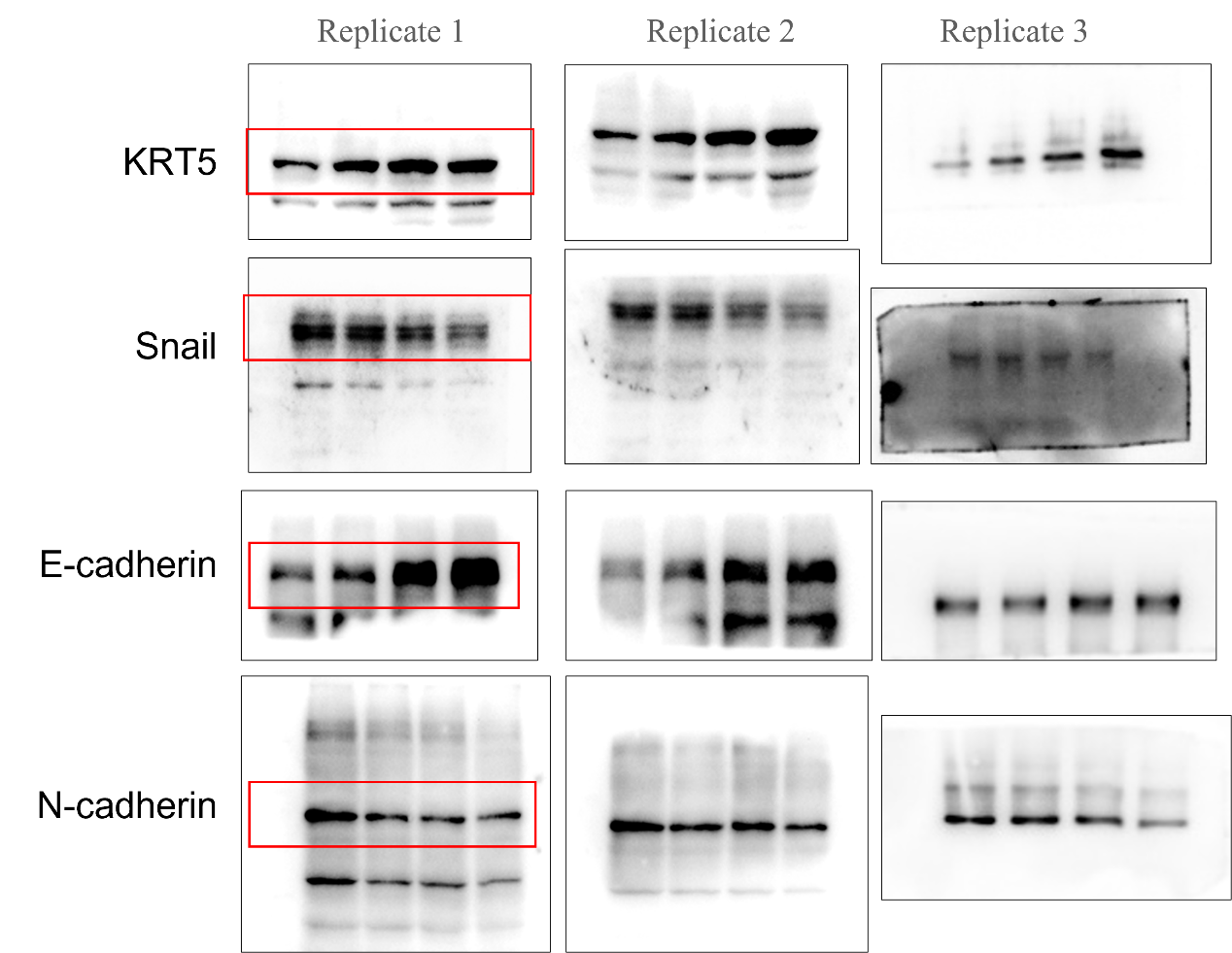


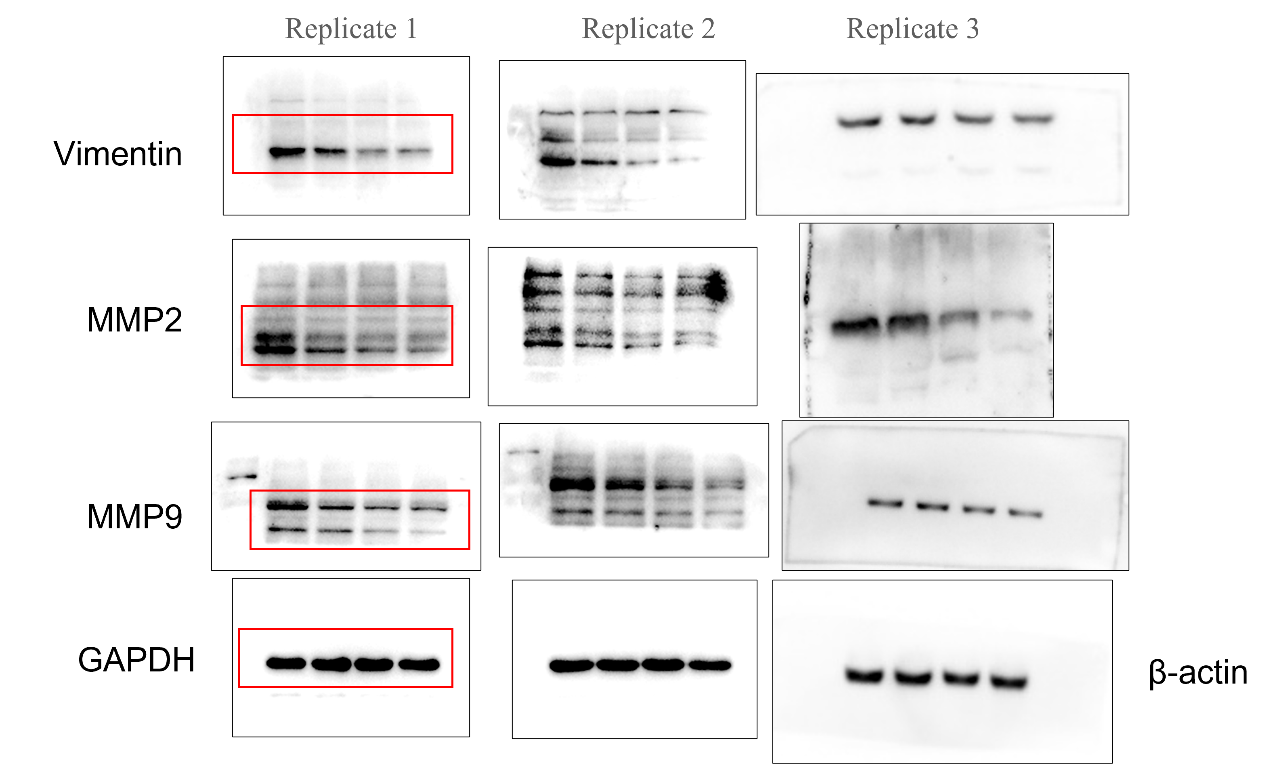


**Figure 8F**

**K450**


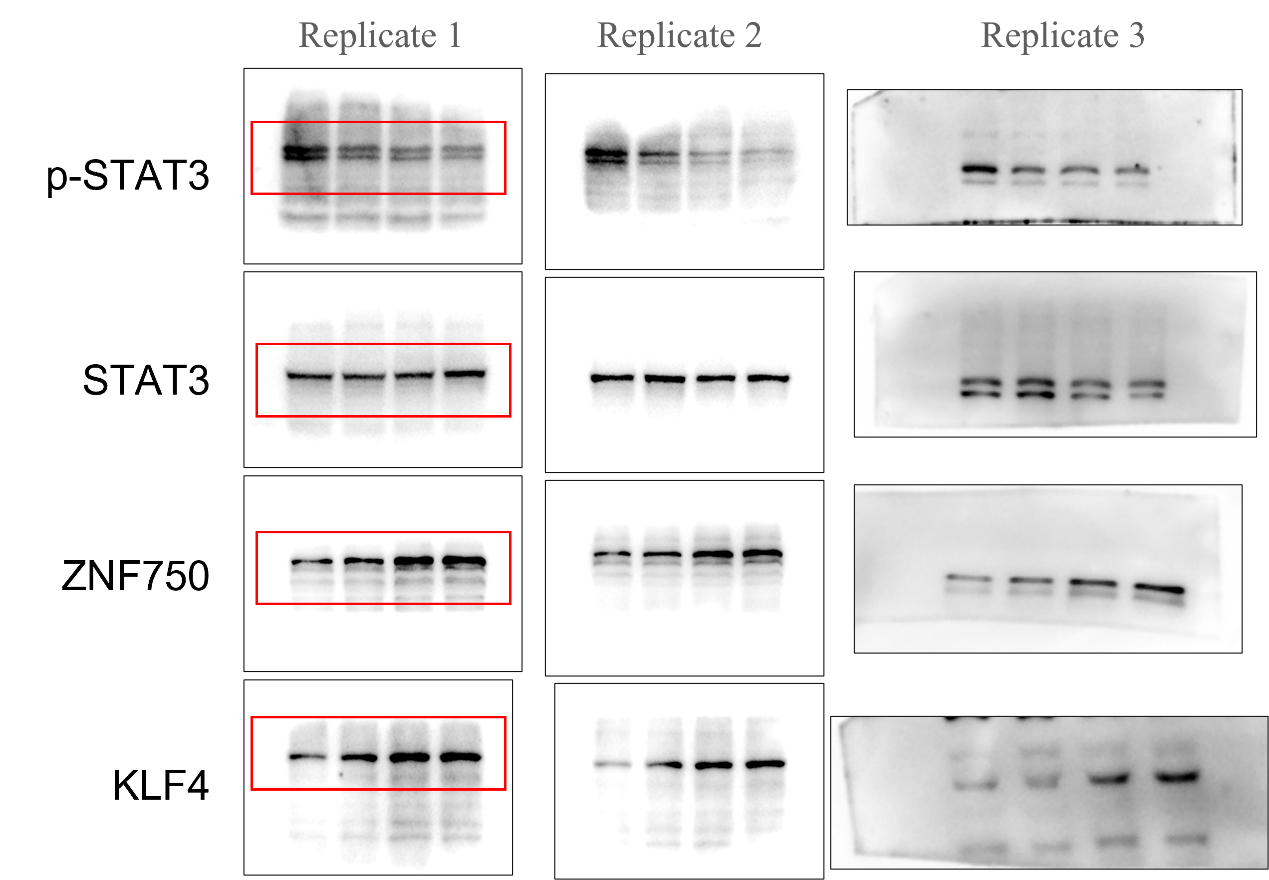


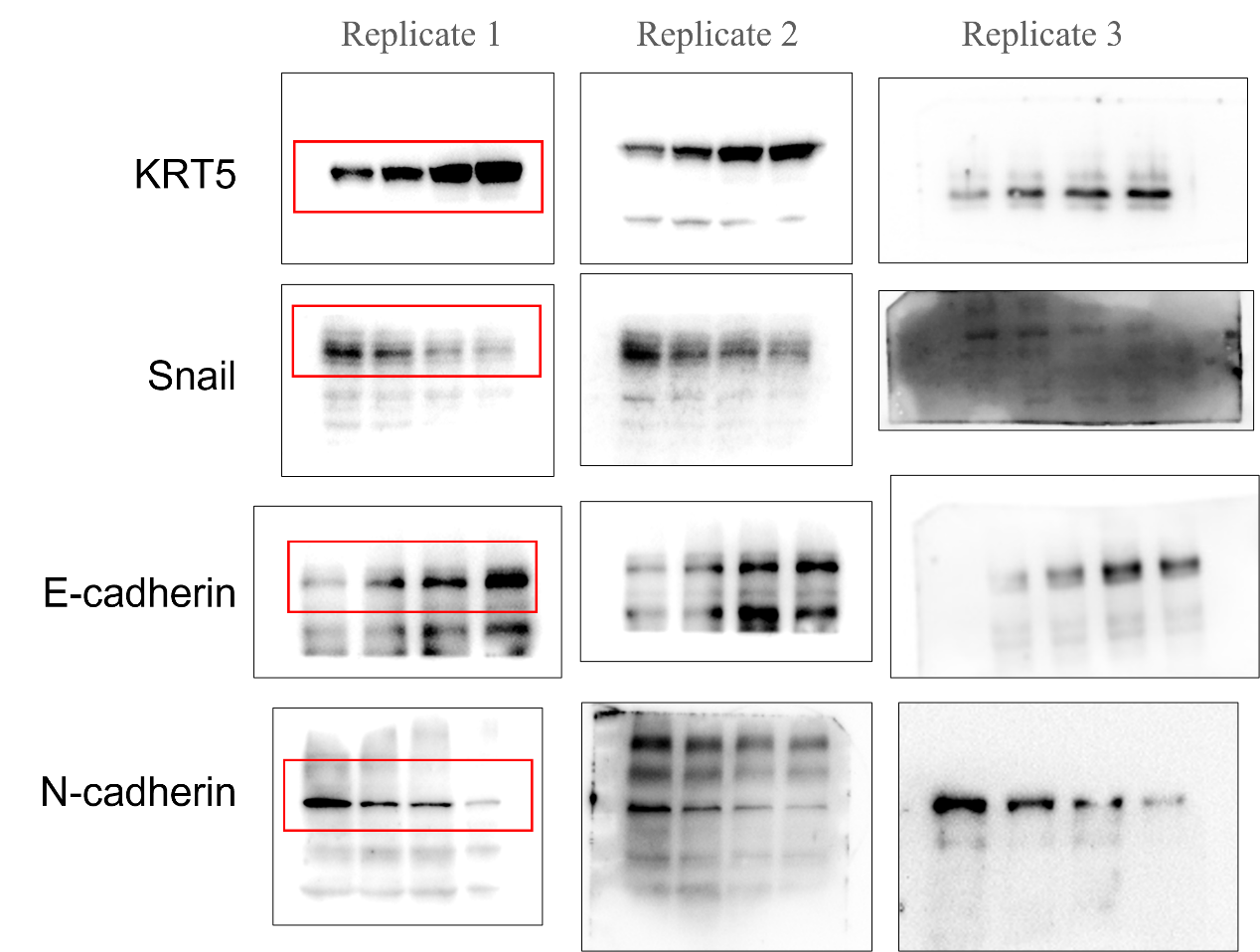


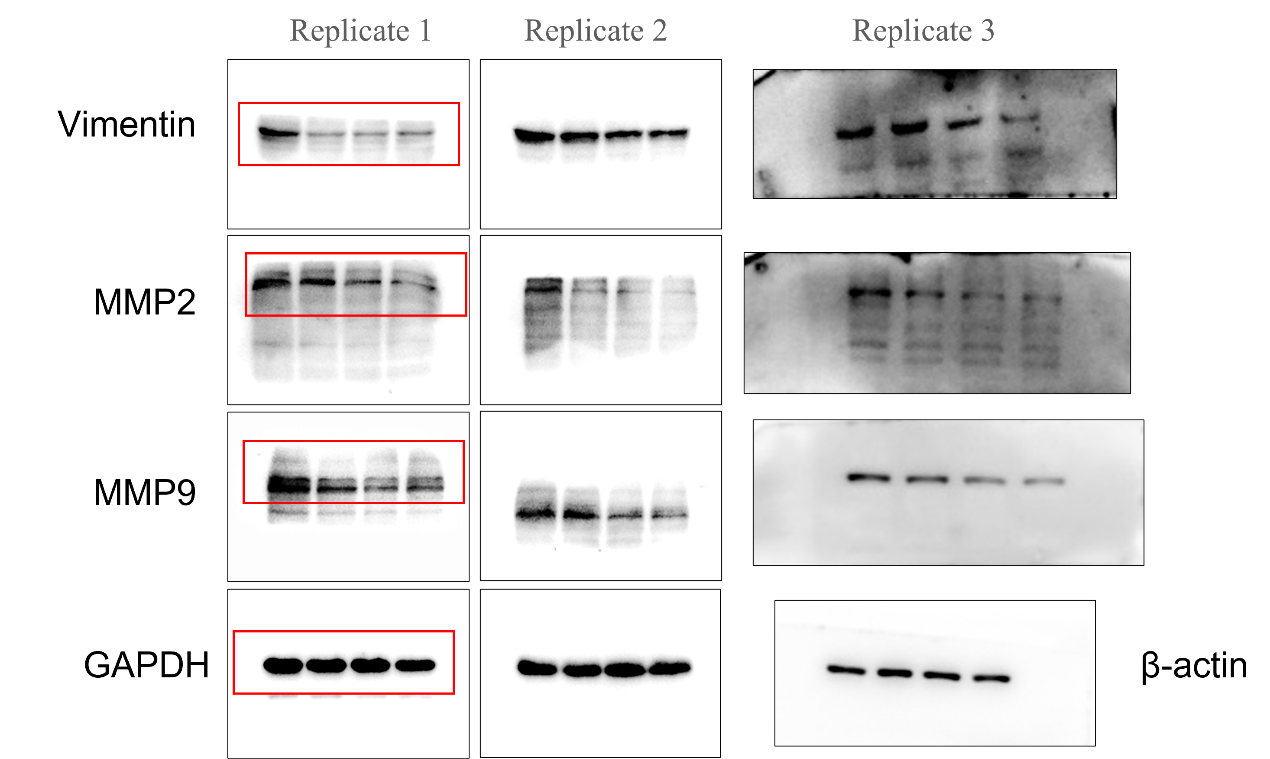

Supplement: Supplementary file 10 — Original Data File [file 41420_2023_1491_MOESM10_ESM.docx]
